# Supplementary material for: Systematic review of endovascular stent grafting versus open surgical repair for the elective treatment of arch/descending thoracic aortic aneurysms
Source: BMJ Open. 2021 Mar 4;11(3):e043323. doi: 10.1136/bmjopen-2020-043323 (PMC7934769; doi:10.1136/bmjopen-2020-043323)
Supplement: Supplementary data [file bmjopen-2020-043323supp001.pdf]

Appendix 1: Search Strategy

Medline/Central/HTA

1. (TEVAR or EVAR).ti,ab.
2. (endovascular adj1 (stent* or repair* or treat* or surg* or (aneurysm? adj1 repair*))).ti,ab.
3. (open adj2 (surg* or repair*)).ti,ab.
4. endoprosthe*.mp.
5. *Aortic Aneurysm, Thoracic/su [Surgery]
6. *Aortic Thoracic/su [Surgery]
7. Thoracic Surgical Procedures/
8. or/1-7
9. exp Aortic Aneurysm, Thoracic/
10. (thorac* adj2 aneurysm*).ti,ab.
11. (descending adj1 aort* adj1 aneurysm*).ti,ab
12. TAA*.ti,ab.
13. (arch adj3 aneurysm*).ti.ab.
14. or/9-13
15. morbid*.mp.
16. mortalit*.mp.
17. outcome?.mp.
18. complication*.mp.
19. (rupture? or endoleak?).mp.
20. device migration.mp.
21. (ae or co or de).fs

22. (safe or safety or side effect* or undesirable effect* or treatment emergent or tolerability or toxicity or adrs or (adverse adj2 (effect or effects or reaction or reactions or event or events to outcome or outcomes))).ti,ab.
23. ((technical or surg*) adj2 (advance* or improve*)).mp.
24. (quality adj2 life).mp.
25. Mortality/
26. Mortbidity/
27. Postoperative complications/
28. Intraoperative complications/
29. Aortic Aneurysm, Thoracic/co
30. Conversion to Open Surgery/
31. "Quality of Life"
32. or/15-31
33. 8 and 14 and 32
34. ("clinical trial" or "clinical trial, phase I" or "clinical trial, phase ii" or clinical trial phase iii or clinical trial, phase iv or controlled clinical trial or "multicentre study" or "randomized controlled trial").pt. or double-blind method/ or clinical trials, phase iii as topic/ or clinical trials, phase iv as topic/ or controlled clinical trials as topic/ or randomized controlled trials as topic/ or early termination of clincal trials as topic/ or multicenter studies as topic/ or ((randomi?ed adj7 trial*) or (controlled adj3 trial*) or (clinical adj2 trial*) or ((single or doubl* or tripl* or treb*) and (blind* or mask*))).ti,ab,kw. or ("4 arm" or "four arm").ti,ab,kw.
35. cohort studies/ or longitudinal studies/ or follow-up studies/ or prospective studies/ or retrospective studies/ or cohort.ti,ab. or longitudinal.ti,ab. or prospective.ti,ab. or retrospective.ti,ab. or quasi-experiment.ti,ab.

36. Clinical Trial.pt. or random*.tw. or Clinical Trials/ or clinical trial.tw. or Follow-up Studies/ or prospectiv*.tw. or control*.tw.
37. or/34-36
38. 33 and 37

EMBASE

1. (TEVAR or EVAR).ti,ab.
2. (endovascular adj1 (stent* or repair* or treat* or surg* or (aneurysm? adj1 repair*))).ti,ab.
3. (open adj2 (surg* or repair*)).ti,ab.
4. endoprosthe*.mp.
5. Thoracic Aorta Aneruysm/su [Surgery]
6. Thoracoabdominal aorta aneurysm/su [Surgery]
7. Thoracic Aorta/su [Surgery]
8. exp Thoracic aorta surgery/
9. or/1-8
10. exp Thoracic Aorta Aneurysm/
11. exp Thoracoabdominal aorta aneurysm/
12. Thoracic Aorta/
13. (thorac* adj2 aneurysm*).ti,ab.
14. (descending adj1 aort* adj1 aneurysm*).ti,ab.
15. TAA*.ti,ab.
16. (arch adj3 aneurysm*).ti,ab.
17. or/10-16
18. (outcome? or mortalit*).mp.
19. complication*.mp.
20. ((technical or surg*) adj2 (advance* or improve*)).mp.

21. (quality adj2 life).mp.
22. Mortality/
23. Postoperative complication/
24. peroperative complication/
25. Thoracic Aorta Aneurysm/co
26. “Quality of Life”/
27. or/18-26
28. (Randomised Controlled Trial or Pragmatic Clinical Trial).pt or exp Randomized Controlled Trials as Topic/ or “Randomized Controlled Trial (topic)”/ or Randomized Controlled Trial/ or Randomization/ or Random Allocation/ or Double-Blind Method/ or Double Blind Procedure/ or Double-Blind Studies/ or Single-Blind Method/ or Single Blind Procedure/ or Single-Blind Studies/ or Placebos/ or Placebo/ or (random* or sham or placebo*).ti,ab,hw,kf,kw. or ((singl* or doubl*) adj (blind* or dumm* or mask*)).ti,ab,hw,,kw. or ((tripl* or trebl*) adj (blind* or dumm* or mask*)).ti,ab,hw,,kw.
29. random:.tw. or placebo:.mp. or double-blind:.mp.
30. controlled clinical trial/ or clinical trial/ or exp controlled study/ or randomized controlled trial/ or exp quasi experimental study/
31. or/28-30
32. 9 and 17 and 27 and 31
33. limit 32 to (human and yr=”1994-Current”)
34. exp cohort analysis/ or exp longitudinal study/ or exp prospective study/ or exp follow up/ or cohort*.tw.
35. limit 34 to (human and yr=”1994-Current”)
36. 9 and 17 and 27 and 35
37. 36 not 33
38. 33 or 37

Appendix 2: Data extraction table

Study	**Von Allmen et al 2014**	**Goodney et al 2011** (2 analyses*)	**Gore TAG**	**Hughes et al 2014**	**Piffaretti et al 2007**
**METHODS**					
**Study Design**	Comparative cohort	Comparative cohort	Comparative cohort	comparative cohort	Comparative cohort
**Method of matching**	None	1. None<br>2. propensity matched	ESG patients judged eligible for OSR	None	OSR patients judged eligible for ESG
**Inclusion Criteria**	Hospital diagnosis code 171.2 (TAA without mention of rupture) from ICD-10 score linked to	Patients diagnosed with a thoracic aneurysm according to ICD9 diagnosis code:	Fusiform descending thoracic aortic aneurysm at least twice the size of the normal adjacent aorta or saccular	Nationwide inpatient Sample database of patients >18 years old who had	Elective, Chronic fusiform or saccular aneurysm of the descending thoracic aorta, OSR patients

	operations code according to OPCS-4, linked with all-cause mortality from ONS and ICD-10, only OPCS-4 codes: L18.2, L19.2, L20.2, L21.2	Intact thoracic aneurysms 441.2<br>Patients had undergone an open or TEVAR procedure according to ICD9 procedure codes:<br>Open 38.35, 38.45<br>TEVAR 39.73, 39.79	aneurysm, Life expectancy >2 years<br>Surgical candidate<br>Male or infertile female >21 years old<br>Descending aorta must be clampable distal to the left carotid artery, and distal anastomosis must be performed proximal to the celiac axis<br>Inner aortic diameter of 23-37mm adjacent to aneurysm<br>Lack of significant thrombus of calcification in landing zones	undergone an open or endovascular repair of non-ruptured thoracic aortic aneurysm. Patients selected using ICD-9 procedures codes 39.73 and 38.45.	would have been a candidates for ESG due to morphological criteria, Selected on surgeon's opinion of lower risk of ESG

			Minimum of 2cm of normal thoracic aorta proximal and distal to aneurysm<br>Aortic taper of no more than 4mm or ability to treat with more than one graft		
**Exclusion Criteria**	Dissection, Abdominal aneurysms, Non-identified area aneurysm, Ascending aneurysm, Suprarenal aneurysm, Non-	Patients with ICD 9 diagnosis codes indicating ascending thoracic aneurysms or cardiopulmonary bypass occurring with circulatory arrest. Thoracoabdominal aneurysm, thoracic aortic	Mycotic aneurysm, Hemodynamically unstable ruptured aneurysm , Major operation (other than planned subclavian to carotid transposition or bypass) within 30 days, MI or CVA within 6 weeks, Creatinine >2.0mg/dL, Connective tissue	NA	Traumatic, Mycotic thoracic aneurysms, Dissections, Aneurysms of the ascending aorta, Aneurysms of the aortic arch, Aortic ulcers

	aortic procedures, age <50yrs	dissection, other aortic pathology. IC9 procedure codes indicating debranching or other procedures to extend endovascular landing zones (39.24, 39.25,	disorder, Acute or chronic aortic dissection, Planned occlusion of carotid or celiac arteries, Documented drug abuse within 6 months, Participation in another investigational device or drug study within 1 year		
**Definition of the short-term**	30 days post-procedure	latest occurring of discharge or 30 days post-intervention	latest occurring of discharge or 30 days post-intervention	post-procedure hospital stay	30-days post-intervention
**PARTICIPANTS**					
**Country**	UK	USA	USA	USA	Italy

**Setting**	Multi-centre – Hospital episode statistics	Multi-centre – Medicare data	Multi-centre	Multi-centre	Single-centre
**Time frame**	2006 to 2011	1. 1998-2007<br>2. 2003-2007	ESG 1999- 2001, OSR pre 1999 (n=44) & 1999-2001 (n=50)	1998-2007	ESG 2000-2007,<br>OSR 1996-2000
**Recruitment**	Total 618<br>ESG 354<br>OSR 264	1. Total 13,998 (ESG 2433, OSR 11,565)<br>2. Total 1100 (ESG 550, OSR 550)	Total 234<br>ESG 140<br>OSR 94	Total 8967<br>ESG 712<br>OSR 8255	Total 28<br>ESG 17<br>OSR 11
**Age summary**	median (IQR)<br>ESG 73(66,78), OSR 71 (63-76)	mean (95% C.I.)<br>1. ESG 75.9 (75.6, 76.1), OSR 73.8 (73.7, 73.9)<br>2.<br>ESG 71.1 (70.8, 71.44),<br>OSR 70.7 (70.7, 71.1)	mean(SD)<br>ESG 75.9 (10.4), OSR 68.2 (10.2)	median (IQR)<br>ESG 72 (63- 78),<br>OSR 63 (52, 72)	mean (SD)<br>ESG 66 (10),<br>OSR 61 (13)

**Proportion male**	ESG 232 (65.5%), OSR 137 (51.9%)	% (95% C.I.): 1. ESG 58.7 (56.7, 60.7), OSR 55.4 (54.8, 56.3) 2. ESG 64.0 (59.9, 68.0), OSR 68.1% (64.2, 72.0)	ESG 90(57%), OSR 48 (51%)	5415 (61.3%) ESG, 437 (65.6%) OSR	ESG 14 (82%), OSR 8 (73%)
**Black race**	NR	% (95% C.I.): 1. ESG 7.5 (6.4, 8.5), OSR 3.8 (3.4, 4.5) (p=0.001) 2. ESG 2.7 (1.4, 4.1), OSR 2.7 (1.4, 4.0) (p=0.99)	NR	NR	NR
**Charlson comorbidity score**	NR	% (95% C.I.): 1. ESG 1.46 (1.38, 1.54), OSR 0.83 (0.80, 0.86) (p=0.00001)	NR	NR	NR

		2. ESG 0.5 (0.41, 0.60), OSR 0.44 (0.35, 0.53) (p=0.34)			
**Diabetes**	NR	% (95% C.I.):<br>1. ESG 8.00 (6.7, 8.8), OSR 4.10 (3.7, 4.5) (p=0.001)<br>2. ESG 4.90 (3.0, 6.7), OSR 2.90 (1.5, 4.3) (p=0.09)	NR	684 (13.2%) ESG, 94 (8.3%) OSR (p<0.001)	NR
**Myocardial infarction**	NR	% (95% C.I.):<br>1. ESG 7.00 (6.00, 8.1), OSR 5.10 (4.6, 5.4) (p=0.001)<br>2. ESG 2.50 (1.2, 3.8), OSR 3.40 (1.9, 4.9) (p=0.38)	-NR	NR	NR
**Congestive heart failure**	NR	% (95% C.I.):	ESG 13 (9%), OSR 9 (10%) (p=1.00)	NR	NR

		1. ESG 10.85% (9.6%- 12.0%), OSR 10.60% (10.1%- 11.2%) (p=0.16)<br>2. ESG 3.00 (1.6, 4.5), OSR 4.70 (2.9, 6.5) (p=0.16)			
**Cerebrovascular disease**	NR	% (95% C.I.):<br>1. ESG 5.09% (4.2%-5.9%), OSR 3.20% (2.9%- 3.5%) (p=0.65)<br>2. ESG 2.00 (0.5, 2.6), OSR 2.00 (0.8, 3.1) (p=0.65)	NR	NR	4 (23%) ESG, 1 (9%) OSR (p=NS)
**COPD**	NR	% (95% C.I.):<br>1. ESG 22.04% (20.3%- 23.6%), OSR 10.65% (10.0%-11.2%) (p=0.47)	ESG 56 (40%), OSR 36 (38%) (p=0.89)	247 (34.7%) ESG, 1483 (17.9%) (P<0.001)	10 (59%) ESG, 6 (54%) OSR (p=NS)

		2. ESG 4.00 (2.3, 5.6), OSR 4.90 (3.0, 6.7) (p=0.47)			
**Chronic renal failure**	NR	% (95% C.I.):<br>1. ESG 4.9% (4.0%- 5.7%), OSR 0.88% (0.07%- 0.10%) (p=0.70)<br>2. ESG 0.01 (0, 1.4), OSR 0.50 (0, 1.1) (p=0.70)	NR	90 (12.6%) ESG, 126 (1.5%) OSR (p<0.001)	4 (23%) ESG, 1 (9%) OSR (p=NS)
**History of malignancy**	NR	% (95% C.I.):<br>1. ESG 5.71% (4.7%-6.6%), OSR 2.91% (2.6%- 3.2%) (p=0.82)<br>2. ESG 1.80 (0.6, 2.9), OSR 1.60 (0.5, 2.6) (p=0.82)	NR	NR	NR
**History of smoking (current or past)**	NR	NR	ESG 117 (84%), OSR 77 (82%) (p=0.86)	NR	NR

**BMI (kg/m2)**	NR	NR	mean (SD): ESG 26.4 (4.7), OSR 26.9 (5) (p=0.44)	NR	NR
**Coronary artery disease**	NR	NR	ESG 69 (49%), OSR 34 (36%) (p=0.60)	NR	NR
**Stroke**	NR	NR	ESG 14 (10%), OSR 9 (9%) (p=1.00)	NR	NR
**Peripheral arterial disease**	NR	NR	ESG 22 (16%), OSR 10 (11%) (p=0.33)	NR	NR
**Symptomatic aneurysm**	NR	NR	ESG 30 (21%), OSR 36 (38%) (p=0.007)	NR	NR
**Other concomitant aneurysm:**	NR	NR	ESG 39 (28%), OSR 26 (28%) (p=1.00)	NR	NR
**Renal dialysis**	NR	NR	ESG 2 (1%), OSR 0 (0%) (p=0.52)	NR	NR

**Paraplegia**	NR	NR	ESG 1 (1%), OSR 0 (0%) (p=1.00)	NR	NR
**Cancer**	NR	NR	ESG 27 (19%), OSR 12 (13%) (p=0.21)	NR	NR
**Prior major vascular surgery**	NR	NR	ESG 63 (45%), OSR 52 (55%) (p=0.14)	NR	NR
**Cardiac comorbidities**	NR	NR	NR	265 (37.2%) ESG, 3535 (42.8%) (p<0.001)	NR
**Hypertension**	NR	NR	NR	NR	16 (94%) ESG, 10 (91%) OSR (p=NS)
**Peripheral obstructive disease**	NR	NR	NR	NR	5 (29%) ESG, 2 (18%) OSR (p=NS)
**Ischemic heart disease**	NR	NR	NR	NR	6 (35%) ESG, 5 (45%) OSR (p=NS)

**Cardiac Surgery**	NR	NR	NR	NR	(18%) ESG, 0 (0%) OSR (p=NS)
**SHORT- TERM OUTCOMES**					
**All-cause mortality**	ESG 21 (5.9%) OSR 4 (1.5%) Adjusted OR: 0.79	1. ESG 6.1% (95%CI 5.1%, 7.0%) OSR 7.1% (95%CI 6.7%, 7.6%) 2. ESG 4.2% (95% C.I. 2.5%-5.8%) OSR 4.5% (95% C.I. 2.8%-6.2%)	ESG 3 (2.1%) OSR 11 (11.7%) Adjusted OR: 0.54	ESG 25 OSR 379	ESG 1 (6%) OSR 2 (20%)
**Re-intervention**	ESG 21 (5.9%) OSR 4 (1.5%) Adjusted OR: 2.97 (1.09, 8.10)	NR	NR	NR	NR

**Paraplegia/ Paraperisis**	NR	NR	ESG 4 (3%)<br>OSR 13 (14%)	NR	NR
**Cerebral Vascular Accident**	NR	NR	ESG 5 (4%)<br>OSR 5 (4%)	NR	ESG 2 (9%)<br>OSR 1 (12%)
**Neurological-unspecified type**	NR	NR	NR	ESG 20 (2.8%)<br>OSR 273 (3.3%)<br>Adjusted OR: 0.38	NR
**Respiratory failure**	NR	NR	ESG 5 (4%)<br>OSR 19 (20%)	NR	NR
**Pneumonia**	NR	NR	NR	NR	ESG 2 (12%)<br>OSR 3 (27%)
**Pulmonary-unspecified type**	NR	NR	NR	ESG 17 (2.4%)<br>OSR 462 (5.6%)<br>Adjusted OR: 0.48	NR
**Myocardial Infarction**	NR	NR	ESG 0 (0%)<br>OSR 1(1%)	NR	ESG 0 (0%)<br>OSR 1 (9%)

**Cardiac-unspecified type**	NR	NR	NR	ESG 28 (2.9%)<br>OSR 1252 (15.2%)<br>Adjusted OR: 0.24	NR
**Peripheral vascular disease**	NR	NR	ESG 20 (14%)<br>OSR 4 (4%)	NR	NR
**Renal failure**	NR	NR	ESG 2 (1%)<br>OSR 12 (13%)	NR	NR
**Wound infection/dehiscence**	NR	NR	ESG 5 (4%)<br>OSR 10 (11%)	NR	NR
**GI complication**	NR	NR	ESG 3 (2%)<br>OSR 6 (6%)	NR	NR
**Post-implant syndrome**	NR	NR	NR	NR	ESG 4(18%)<br>OSR 0(0%)
**LONG-TERM OUTCOMES**					

All-cause survival	1 year adjusted OR: 1.1 95%CI [0.70-1.73], p=0.667 5 year adjusted HR: 1.45 95%CI [1.08-1.94], p=0.013	NR	NR	NR	NR
**aortic-related survival**	5 year adjusted HR: 1.27 95%CI [0.81-1.98], p=0.298	NR	NR	NR	NR
**survival from aortic-related re-intervention**	5 year adjusted HR: 1.70 95%CI [1.11-2.60], p=0.014	NR	NR	NR	NR
* 1. Unadjusted cohort, 2. Propensity matched cohort					

Appendix 3: Risk of Bias assessment

Risk of Bias Results using the ROBINS-I risk of bias tool

Domain – source of bias	Goodney et al 2011 *(2 analyses*)*	Gore TAG	Hughes et al 2014	Piffaretti et al 2007	Von Allmen et al 2014
**Confounding**	1. Severe<br>2. Moderate	Severe	Severe	Severe	Severe
**Selection of participants into the study**	1. Low<br>2. Low	Low	Low	Low	Low
**Classification of interventions**	1. Low<br>2. Low	Low	Low	Low	Low
**Deviations from intended interventions**	1. Low<br>2. Low	Low	Low	Low	Low
**Missing data**	1. Low<br>2. Low	Low	Low	Low	Low
**Measurement of outcomes**	1. Low<br>2. Low	Low	Low	Low	Low
**Bias in selection of the reported result**	1. Moderate<br>2. Moderate	Moderate	Moderate	Moderate	Moderate
**Overall**	1. Severe<br>2. Moderate	Severe	Severe	Severe	Severe
* 1. Unadjusted cohort, 2. Propensity matched cohort					
