# Supplementary material for: Systematic review of endovascular stent grafting versus open surgical repair for the elective treatment of arch/descending thoracic aortic aneurysms
Source: BMJ Open. 2021 Mar 4;11(3):e043323. doi: 10.1136/bmjopen-2020-043323 (PMC7934769; doi:10.1136/bmjopen-2020-043323)
Supplement: Author's manuscript [file bmjopen-2020-043323.draft_revisions.pdf]

BMJ Open

BMJ Open is committed to open peer review. As part of this commitment we make the peer review history of every article we publish publicly available.

When an article is published we post the peer reviewers' comments and the authors' responses online. We also post the versions of the paper that were used during peer review. These are the versions that the peer review comments apply to.

The versions of the paper that follow are the versions that were submitted during the peer review process. They are not the versions of record or the final published versions. They should not be cited or distributed as the published version of this manuscript.

BMJ Open is an open access journal and the full, final, typeset and author-corrected version of record of the manuscript is available on our site with no access controls, subscription charges or pay-per-view fees (http://bmjopen.bmj.com).

If you have any questions on BMJ Open's open peer review process please email info.bmjopen@bmj.com

BMJ Open

# A Systematic Review of Endovascular Stent Grafting Versus Open Surgical Repair for the Treatment of Arch/Descending Thoracic Aortic Aneurysms

| | |
|---|---|
| Journal: | *BMJ Open* |
| Manuscript ID | bmjopen-2020-043323 |
| Article Type: | Original research |
| Date Submitted by the Author: | 11-Aug-2020 |
| Complete List of Authors: | McCarthy, Andrew; Northumbria University, Faculty of Health and Life Sciences<br>Gray, Joanne; Northumbria University, Health and Life Sciences<br>Sastry, Priya; John Radcliffe Hospital, Cardiothoracic Surgery<br>Sharples, Linda; London School of Hygiene and Tropical Medicine, Medical Statistics<br>Vale, Luke; Newcastle University, Health Economics Group, Population Health Sciences Institute<br>Cook, Andrew; University of Southampton, Wessex Institute; University of Southampton, Southampton Clinical Trials Unit<br>Mcmeekin, Peter; Northumbria University, Faculty of Health and Life Sciences<br>Freeman, Carol; Papworth Hospital NHS Foundation Trust, Papworth Trials Unit Collaboration<br>Catarino, Pedro; Papworth Hospital NHS Foundation Trust<br>Large, Stephen; Papworth Hospital NHS Foundation Trust, Cardiac Surgery |
| Keywords: | Cardiothoracic surgery < SURGERY, Vascular surgery < SURGERY, Thoracic surgery < SURGERY |
| | |

SCHOLARONE™ Manuscripts

*I, the Submitting Author has the right to grant and does grant on behalf of all authors of the Work (as defined in the below author licence), an exclusive licence and/or a non-exclusive licence for contributions from authors who are: i) UK Crown employees; ii) where BMJ has agreed a CC-BY licence shall apply, and/or iii) in accordance with the terms applicable for US Federal Government officers or employees acting as part of their official duties; on a worldwide, perpetual, irrevocable, royalty-free basis to BMJ Publishing Group Ltd ("BMJ") its licensees and where the relevant Journal is co-owned by BMJ to the co-owners of the Journal, to publish the Work in this journal and any other BMJ products and to exploit all rights, as set out in our licence.*

*The Submitting Author accepts and understands that any supply made under these terms is made by BMJ to the Submitting Author unless you are acting as an employee on behalf of your employer or a postgraduate student of an affiliated institution which is paying any applicable article publishing charge ("APC") for Open Access articles. Where the Submitting Author wishes to make the Work available on an Open Access basis (and intends to pay the relevant APC), the terms of reuse of such Open Access shall be governed by a Creative Commons licence – details of these licences and which Creative Commons licence will apply to this Work are set out in our licence referred to above.*

*Other than as permitted in any relevant BMJ Author's Self Archiving Policies, I confirm this Work has not been accepted for publication elsewhere, is not being considered for publication elsewhere and does not duplicate material already published. I confirm all authors consent to publication of this Work and authorise the granting of this licence.*

For peer review only

# A Systematic Review of Endovascular Stent Grafting Versus Open Surgical Repair for the Treatment of Arch/Descending Thoracic Aortic Aneurysms

Andrew McCarthy [1], Joanne Gray [1], Priya Sastry [2], Linda Sharples [3], Luke Vale [4], Andrew Cook [5,6,7]; Peter McMeekin [1], Carol Freeman [8], Pedro Catarino [9], Stephen Large [9]

[1] Faculty of Health and Life Sciences, Northumbria University, Newcastle Upon Tyne, UK

[2]John Radcliffe Hospital, Oxford University Hospitals, UK

[3]Department of Medical Statistics, London School of Hygiene and Tropical Medicine, London, UK

[4]Health Economics Group, Population Health Sciences Institute, Newcastle University, Newcastle Upon Tyne, UK

[5] Wessex Institute, University of Southampton, UK
[6] Southampton Clinical Trials Unit, University of Southampton, UK

[7] University Hospital Southampton NHS Foundation Trust, UK

[8] Papworth Trials Unit Collaboration, Royal Papworth Hospital NHS Foundation Trust, Cambridge

[9]Royal Papworth Hospital NHS Foundation Trust, Cambridge

**Corresponding Author:**

**Andrew McCarthy-** Coach Lane Campus West, Northumbria University, Newcastle upon Tyne, United Kingdom, NE7 7XA

andrew2.mccarthy@northumbira.ac.uk

Tele: 0191 215 6718



For peer review only - http://bmjopen.bmj.com/site/about/guidelines.xhtml

## ABSTRACT

### Aims

A thoracic aortic aneurysm (TAA) is an abnormal dilatation of the aorta at any part of its course from the heart to the diaphragm. If untreated, aneurysms will continue to expand with consequent increased risk of serious complications like dissection, rupture, or death. Our objective was to review comparisons of the effectiveness of endovascular stent grafting (ESG) against open surgical repair (OSR) for treatment of chronic arch or descending thoracic aortic aneurysms (TAA).

### Methods and Results

Studies comparing ESG and OSR were sought; including randomised, quasi-randomised and non-randomised controlled trials, cohort studies and case-control studies matched on key outcomes. Patients had to have received elective treatment for arch/descending thoracic aortic aneurysms. MEDLINE, EMBASE, CENTRAL, WHO International Clinical Trials Routine data collection, current controlled trials, clinical trials, and the NIHR portfolio were searched.

Five comparative cohort studies met inclusion criteria, reporting a total of 3955 ESG and 21,197 OSR patients. Meta-analysis of unadjusted short-term all-cause mortality favoured ESG (odds ratio: 0.75; 95 %CI (0.55-0.1.03)). Heterogeneity was identified between the larger and smaller studies. Meta-analysis of adjusted short-term all-cause mortality favoured ESG odds ratio 0.71, 95%CI (0.51, 0.98), no heterogeneity present. Long-term survival from all-cause mortality favoured OSR in larger studies and ESG in smaller studies. Freedom from re-interventions in the long-term favoured OSR. Studies reporting short-term non-fatal complications suggest fewer events following ESG although limited data prevented meta-analysis.

### Conclusion

There is limited and increasingly dated evidence on the comparison of ESG and OSR for treatment of arch/descending thoracic aortic aneurysms.




For peer review only - http://bmjopen.bmj.com/site/about/guidelines.xhtml

**KEY WORDS**

Systematic Review, Meta-analysis, Thoracic Aortic Aneurysm, Open Surgical Repair, Endovascular Stent Graft

**STRENGTHS AND LIMITATIONS OF THIS STUDY**

Strength:

- To the authors knowledge this is the first systematic review to investigate two-armed studies looking only at ESG versus OSR for treatment of chronic arch or descending thoracic aortic aneurysms. Other reviews include dissections, ruptures and treatment for other conditions.
- This systematic review also considers evidence from both randomized and non-randomized study designs.

Limitation:

- Statistical analysis of the non-fatal complications was not possible due to the limited and inconsistent reporting of such complications.

For peer review only



For peer review only - http://bmjopen.bmj.com/site/about/guidelines.xhtml

## INTRODUCTION

### Rationale

A thoracic aortic aneurysm (TAA) is an abnormal expansion of the aorta at any part of its course from the heart to the diaphragm. Aneurysms manifest with acute symptoms or they may be chronic. If untreated, TAA can continue to expand and the annual risk of aneurysm rupture increases from 2% in aneurysms smaller than 5cm in diameter to 7% in aneurysms over 6cm in diameter[1]. The majority of aneurysms (and therefore studies) centre on the ascending or abdominal segments of the aorta, arch and descending aneurysms are relatively under-studied. Therefore this review focuses on chronic aneurysms affecting the arch or descending thoracic aorta.

There are currently two main methods of repair for arch/descending TAA: Open Surgical Repair (OSR) and Endovascular Stent Grafting (ESG). OSR involves replacing the aneurysmal segment of aorta with a surgical graft during open surgery using heart-lung bypass. As the arch and descending aorta supply blood to the brain and spinal cord, OSR is high risk, with reported mortality and paraplegia rates of approximately 5% and 10% [2] respectively. In ESG a stent is placed across the aneurysm, providing a new conduit through which the blood flows. ESG is most often selected for patients who are deemed at high risk of death or permanent disability from OSR. However, some patients may not have a suitable anatomy/morphology for ESG procedures [2]. Concerns have also been raised about long-term durability and safety of the implanted devices [3 4] due to complications such as stent migration or endoleaks, with the incidence of late endoleaks (leaks around or through the stent graft that occur more than year after insertion) reported as being between 2.5% and 27.8% [5]. The need for re-intervention due to technical failures of the stent following ESG is also high (23% at 5 years) [6 7]. With each re-intervention there is an added risk of complication either due to the increased complexity of the procedure or deteriorating health of the patient [2].



For peer review only - http://bmjopen.bmj.com/site/about/guidelines.xhtml

For peer review only

One previous systematic review comparing only elective OSR and ESG for descending and arch TAAs has been reported [8]. This systematic review [8] was restricted to data from randomized control trials in which patients were randomly assigned to ESG and OSR, but no eligible trials were identified when searching relevant data based up to Jan 2016. There are other systematic reviews [9 10] that have included non-randomized studies; however, these reviews also included emergency cases and other thoracic aortic pathologies (e.g. dissection or rupture). As such, they may not represent a true comparison of elective ESG and OSR procedures for descending thoracic aortic aneurysms. Given the lack of RCT evidence, this systematic review includes non-randomized comparative studies of the relative effectiveness of OSR or ESG.

The decision to operate requires a favourable balance between the risks and benefits of that intervention compared to those attached to the expected outcomes of the disease. This balance is in turn influenced by a number of factors. Aneurysm size correlates to the risk of death (if left untreated) and so patients are usually referred once the aneurysm crosses a chosen threshold. At that point, patient fitness (*and life expectancy*), the reality or possibility of an intrinsic weakness affecting the whole aorta, the technical feasibility of the proposed intervention and patient wishes, come into play. This systematic review takes a step back from these issues and examines only the outcomes for patients in whom a decision to intervene has been made. This review is an essential starting point for the analysis of effective treatments for thoracic aortic aneurysms.

**Objectives**

The aim of this systematic review was to assess the effectiveness of endovascular stent grafting (ESG) compared to open surgical repair (OSR) as a treatment for chronic arch or descending TAA.



For peer review only - http://bmjopen.bmj.com/site/about/guidelines.xhtml

## METHODS

### Protocol and registration

The review protocol was registered in the PROSPERO database (CRD42017054565)[11]

### Eligibility criteria

### Study designs

All identified studies that directly compared ESG and OSR, including RCTs, quasi-randomised and non-randomised controlled trials, comparative cohort studies and case-control studies matched on main outcomes were sought [12].

Non-randomised studies that controlled for selection bias were included – irrespective of study size. Acceptable 'anti-selection bias" methods included matching patients at baseline for important covariates, matching patients using propensity scores or adjustment for important covariates during analysis. Non-randomised studies that did not use these methods but included larger sample sizes (more than 250 patients) were also included as they have the potential to reduce sampling variation relative to smaller unmatched studies[13].

Non-English language papers were noted and, where possible, data were extracted from the abstract. No translation of full text articles was possible.

### Study Participants

Study participants had to receive elective treatments for arch and, or descending thoracic aortic aneurysms. Where emergency interventions or other thoracic aortic conditions (such as intramural haematoma, rupture, and dissection) were reported, studies were excluded, unless results for patients receiving elective treatment for arch/descending TAA were reported separately. Studies that reported secondary (re-intervention) procedures for arch/descending TAAs were included only if the original, primary procedure for each participant could be identified.



For peer review only - http://bmjopen.bmj.com/site/about/guidelines.xhtml

**Study interventions**

Open Surgical Repair was defined as replacement of the aneurysmal aorta with a prosthetic conduit via a sternotomy or thoracotomy with circulatory support. Endovascular stent grafting was defined as transluminal introduction of a stent-graft under X-ray guidance.

**Outcome measures**

This review assessed short-term and long-term clinical effectiveness. Short-term was defined as either within 30-days of the intervention and/or time until discharge following the intervention. Since most studies did not report long-term outcomes conditional on surviving the short-term period, long-term analyses covered the time from intervention until the end of follow-up. Primary outcomes were all-cause mortality and aneurysm-related mortality. Secondary outcomes were re-interventions and non-fatal complications. The latter were sub-classified as vascular, neurological, cardiac, respiratory and 'other' complications.

**Information sources**

MEDLINE, EMBASE, CENTRAL, WHO International Clinical Trials Routine data collection, Current Controlled Trials, Clinical Trials, and the UK National Institute for Health Research portfolio were searched. The search strategy (Appendix 1) was adapted as appropriate for each database (for example to allow for variations in controlled vocabulary terms and syntax [14]). Electronic searches identified publications between 1994, date of first reported case of ESG for descending and arch TAA[15], and March 2020.

Websites of regulatory bodies and Health Technology Assessment agencies were screened for unpublished relevant reports. A citation search of all included studies was conducted via Web of Science. Studies from systematic reviews that satisfied the inclusion criteria of this review were screened for eligibility.

**Study selection**



For peer review only - http://bmjopen.bmj.com/site/about/guidelines.xhtml

For peer review only

All titles and abstracts were screened by one reviewer (AM). A second investigator (JG) reviewed 10% of all titles and abstracts classed as eligible or not eligible and all titles and abstracts classified as unclear by the first reviewer. The full text of selected studies was assessed by one investigator (AM) and classified as relevant, not relevant or unclear according to the inclusion criteria. A second investigator (JG) reviewed 10% of all papers, as well as all studies deemed unclear. Disagreements at either stage were resolved by discussion and where necessary a third reviewer (PM) arbitrated. Where required, a clinical expert (PS) was asked to screen papers.

**Data collection process**

A data extraction form was developed based on The Cochrane Collaboration data collection form [16]. Data were extracted by one reviewer (AM) and checked by a second reviewer (JG) for accuracy. Disagreements were resolved by discussion or arbitration by a third reviewer (PM).

**Data items**

Reported short-term and longer-term all-cause mortality, as well as short-term and longer-term aneurysm-related mortality were collected. All reported complications were recorded and grouped according to expert clinical guidance as described above as neurological, cardiac, respiratory or 'other' complications, with endoleaks following ESG recorded separately.

**Risk of bias in individual studies**

The risk of bias was assessed using the ROBINS-I tool [17] designed for non-randomised studies. The ROBINS-I tool classifies the risk of bias in seven domains (*confounding; selection of participants; classification of interventions; deviations from intended interventions; missing data; measurement of outcomes; selection of the reported results*) and overall as either critical, severe, moderate, low, or unclear.

**Synthesis of results**



For peer review only - http://bmjopen.bmj.com/site/about/guidelines.xhtml

Point estimates and precision of the incidence of short and long-term events and unadjusted and adjusted comparisons (odds ratios (OR) hazard ratios (HR)) were recorded. Meta-analysis of short-term event-comparisons was based on the OR with 95% confidence interval (CI) and conducted using RevMan [18]. An overall treatment effect was calculated for the log (OR) of each outcome using a (normal) random effects model. Where comparisons could not be made, data were tabulated and reported narratively.

Heterogeneity between studies was assessed according to Cochrane guidelines, on clinical criteria, by visual inspection of plots of the data, from the chi-square test for heterogeneity, and the I-squared statistic. An $I^2$ statistic of 25-50% indicated low heterogeneity; 50-75% indicated moderate heterogeneity, and over 75% indicating significant heterogeneity.

## RESULTS

### Study selection

The search identified 5163 unique studies. Of these, 5078 studies were not eligible. Ineligibility was primarily because of irrelevant anatomy (ascending, abdominal, or peripheral aneurysms), irrelevant pathology (acute dissection or other acute aortic syndromes), or because the study pooled data from a mixture of anatomical and pathological situations such that data on chronic arch and DTA aneurysms could not be extracted with confidence. Thus 85 papers underwent full text screening. 75 papers were rejected at this stage leaving 10 papers to be included in the full review. Six papers reported data from the Gore TAG Trial [19-24], leaving five unique comparative studies that fulfilled inclusion criteria. Further details of the selection process are shown in the PRISMA diagram (Figure 1).

Brief summaries of the identified studies are given in Table 1, with detail provided in Appendix 2. Overall, the five studies included data on 3955 ESG and 21,197 OSR patients and all studies were conducted in Europe or the USA. However, two studies had non-overlapping or only partially



For peer review only - http://bmjopen.bmj.com/site/about/guidelines.xhtml

overlapping recruitment periods between the ESG and OSR intervention groups within their respective study (Gore Tag and Piffaretti et al 2007[19 25]). There were clear differences in patient characteristics between the two treatment groups. ESG patients were consistently older, with average differences ranging from 2 to 9 years in all but one analysis (Goodney et al 2011 the propensity matched cohort). Similarly, in most studies the proportion of men was greater in the ESG arm.

For peer review only



For peer review only - http://bmjopen.bmj.com/site/about/guidelines.xhtml

*Table 1: Study characteristics*

| Study | **Von Allmen et al 2014** | **Andrassy et al 2011** | **Goodney et al 2011** | **Goodney et al 2011 (propensity matched cohort)** | **Gore TAG** | **Hughes et al 2014** | **Piffaretti et al 2007** |
|---|---|---|---|---|---|---|---|
| **METHODS** | | | | | | | |
| **Study Design** | Comparative cohort | Comparative cohort | Comparative cohort | Comparative cohort | Comparative cohort | Comparative cohort | Comparative cohort |
| Method of matching | None | None | None | Stratified propensity score | ESG patients judged eligible for OSR | None | OSR patients judged eligible for ESG |
| **PARTICIPANTS** | | | | | | | |
| **Country** | UK | Germany | USA | USA | USA | USA | Italy |
| **Setting** | Multi-centre – Hospital episode statistics | Single centre | Multi-centre – Medicare data | Multi-centre – Medicare data | Multi-centre | Multi-centre | Single-centre |
| **Time frame** | 2006 to 2011 | ESG 1997-2008, | 1998-2007 | 2003-2007 | ESG 1999- 2001, | 1998-2007 | ESG 2000-2007, |



For peer review only - http://bmjopen.bmj.com/site/about/guidelines.xhtml

| | | | | | | | |
|---|---|---|---|---|---|---|---|
| | | OSR 1992-2002 | | | OSR pre 1999 (n=44) & 1999-2001 (n=50) | | OSR 1996-2000 |
| **Recruitment** | Total 618<br>ESG 354<br>OSR 264 OSR | Total 77<br>ESG 53<br>OSR 24 | Total 13,998<br>ESG 2433<br>OSR 11,565 | Total 1100<br>ESG 550<br>OSR 550 | Total 234<br>ESG 140<br>OSR 94 | Total 8967<br>ESG 712<br>OSR 8255 | Total 28<br>ESG 17<br>OSR 11 |
| **Age summary** | median (IQR)<br>ESG 73(66,78),<br>OSR 71 (63-76) | mean (SD)<br>ESG 70(10),<br>OSR 67(12) | mean (95% C.I.)<br>ESG 75.9 (75.6, 76.1), OSR 73.8 (73.7, 73.9) | mean (95% C.I.)<br>ESG 71.1 (70.8, 71.44), OSR 70.7 (70.7, 71.1) | mean(SD)<br>ESG 75.9 (10.4),<br>OSR 68.2 (10.2) | median (IQR)<br>ESG 72 (63- 78),<br>OSR 63 (52, 72) | mean (SD)<br>ESG 66 (10),<br>OSR 61 (13) |
| **Proportion male** | ESG 232 (65.5%),<br>OSR 137 (51.9%) | ESG 32 (60%),<br>OSR 13 (54%) | % (95% C.I.):<br>ESG 58.7 (56.7, 60.7), OSR 55.4 (54.8, 56.3) | % (95% C.I.):<br>ESG 64.0 (59.9, 68.0), OSR 68.1% (64.2, 72.0) | ESG 90(57%),<br>OSR 48 (51%) | 5415 (61.3%) ESG, 437 (65.6%) OSR | ESG 14 (82%),<br>OSR 8 (73%) |
| **OUTCOMES** | | | | | | | |
| **Short-term** | Death from any | Death from any | Death from any | Death from any | Death from any | Death from any | Death from any |

For peer review only

| | | | | | | | |
|---|---|---|---|---|---|---|---|
| | cause<br>Re-intervention<br>All-cause mortality (unadjusted | cause<br>Re-intervention<br>Complications | cause | cause | cause<br>Complications | cause<br>Complications | cause<br>Complications |
| **Long-term** | Time to death from any cause<br>Time to aortic-related death<br>Time to aortic-related re-intervention | Time to death from any cause | Time to death from any cause | Time to death from any cause | Time to death from any cause<br>Time to aneurysm-related death<br>aneurysm-related mortality<br>Time to re-intervention<br>Time to major adverse event | | Time to death from any cause |

For peer review only

**Risk of bias within studies**

One study (Goodney et al 2011) [26] was judged to be of overall moderate risk of bias and four studies were at severe risk of bias due to the potential for confounding (Appendix 3).

Two large cohorts derived from UK and USA administrative databases (Von Allmen et al 2014 and Hughes et al 2014) [27 28] did not attempt to address selection bias at the design stage, but did adjust for some confounders in the analysis. A third study (Goodney et al 2011), using USA administrative data, included both risk-factor-adjusted models and analysis of a subset of the data defined by propensity score matching. Propensity score methods rely on all of the important confounders being available and included[29]. As this study did not include all confounders considered important, such as aneurysm size, this study was judged to have moderate risk of bias due to confounding.

One study (Gore TAG)[19] included only ESG patients who would also have been eligible for OSR and the another study (Piffaretti et al 2007) [25] included only OSR patients who would be suitable for ESG, both according to clinician judgement. Since suitability was decided retrospectively, such matching may not be robust.

A further potential bias with each study was selective reporting. Although the measurements and analyses of the outcomes were clearly defined within each study, no evidence of a pre-study analysis plan was identified.

**Synthesis of results**

**Short-term death from any cause (30-day or in hospital)**

*Unadjusted Mortality:*

All five studies [19 25-28] reported unadjusted 30 day or in-hospital mortality (figure 2). Overall, the odds of short-term mortality were lower in the ESG group than the OSR group (OR: 0.75; 95 %CI. (0.55-1.03)). Moderate heterogeneity was present in the overall analysis ($I^2$= 40%), mainly due to



For peer review only - http://bmjopen.bmj.com/site/about/guidelines.xhtml

differences between the two smaller studies [19 25] and the three larger studies [26-28], with a large $I^2$ for subgroup differences between the larger studies subgroup and the smaller studies subgroup of 84.5%.

For the three larger studies[26-28] (3798 ESG and 21,092 OSR patients), the treatment effect for death was smaller (OR: 0.84 95%CI (0.72, 0.98)) than that for the two smaller studies [19 25](157 ESG and 105 OSR patients, combined OR: 0.18 95%CI (0.06, 0.59)). There was no evidence of heterogeneity in results between these subgroups ($I^2$=0).

*Adjusted Mortality:*

The three larger studies [26-28] included adjusted analysis of short-term mortality (Figure 3). One study used stratified propensity matching. Another study [27] adjusted for preoperative comorbidities, independent predictors of mortality and post-operative complications and the third study adjusted for age and sex. The OR for ESG, relative to OSR, for these three studies combined was 0.71, 95%CI (0.51, 0.98). No evidence of heterogeneity was identified in this meta-analysis ($I^2$=0%)

**All-cause mortality (following hospital discharge or after 30 days)**

Meta-analysis of the longer-term all-cause mortality was not possible as follow-up varied between the four studies that reported this outcome (Gore Tag, Goodney et al 2011, Piffaretti et al 2007 and Von Allmen 2014) [19 25 26 28] . The results in the larger studies (Goodney et al 2011, Von Allmen 2014)[26 28] differed from those in the smaller studies (Gore Tag and Piffaretti et al 2007)[25 30]. We note that the analyses included the short-term deaths as well as those occurring beyond hospital discharge or after 30 days.

Von Allmen et al 2014 reported time to death from any cause up to five years, throughout which OSR had higher overall survival. Adjusting for age and sex, ESG had higher odds of death at one year



For peer review only - http://bmjopen.bmj.com/site/about/guidelines.xhtml

(OR: 1.10 95%CI (0.70, 1.73); p=0.667). Additionally, the hazard ratio for ESG relative to OSR up to five years, adjusting for age and sex, was HR=1.45 (95%CI [1.08, 1.94], p=0.013).

Goodney et al 2011 also reported time to death from any cause up to five years. Adjusting for age, sex, race, era of procedure and Charlson comorbidity score, five-year survival for OSR was higher than for ESG (89% versus 79%, log-rank p<0.0001). In the propensity-matched cohorts, five-year life-table analysis reported higher survival in the OSR group (81% 95%CI (77%-85%)) than for the ESG group (73% 95%CI (68%, 76%)) (log-rank p=0.007).

Time to death from any cause in the Gore TAG study did not differ over five years between the ESG and OSR groups with survival probability at five years in both groups was 63% (log rank P value= 0.625). Piffaretti et al 2007 reported survival overall in the ESG group up to five years and the OSR group up to 13 years. For the five years in which survival is reported in both groups, ESG had a higher survival compared to OSR although this was based on only 4 deaths, and the follow-up periods differed substantially (log rank p>0.05).

**Aneurysm/Aortic-related mortality**

Meta-analysis of Aneurysm/aortic-related mortality could not be conducted. No studies reported aneurysm-related mortality during hospitalization or up to 30 days separately to the long-term. Von Allmen et al 2014 reported aortic-related mortality up to five years. Time to aortic-related death was did not differ between OSR group than the ESG group during the five-year follow-up (log-rank p=0.270). This study[28] also reported adjusted (for age and sex) five year HR of 1.27 (0.81 to 1.98; P =0.298) for ESG patients relative to OSR patients. Gore TAG[19] reported aneurysm-related mortality up to five years, with longer time to aneurysm-related death in the ESG group over this period (log-rank p= 0.012).



For peer review only - http://bmjopen.bmj.com/site/about/guidelines.xhtml

**Short-term re-interventions**

One study reported this outcome (Von Allmen et al 2014[28]). This study reported aortic-related re-interventions in 21 (5.9%) ESG patients and 4 (1.5%) OSR patients, an adjusted OR of 2.97 (95%CI [1.09, 8.10], p=0.033) for ESG versus OSR.

**Long-term re-interventions**

Two studies reported this outcome (Von Allmen et al 2014[28] and Gore TAG [19]). Von Allmen et al 2014[28] reported time to aortic-related re-interventions up to five years. Time to re-intervention was longer in the OSR group compared to the ESG group (log-rank p=0.007), with adjusted (for age and sex) hazard ratio at five years of 1·70 (95%CI [1·11 to 2·60], p =0·014). In Gore TAG[19], time to re-intervention over five years was longer in the ESG group (log-rank P=0.011). We note that these analyses included the short-term re-interventions.

**Complications**

Table 2 summarises the short-term complications reported in all five studies[19 25-28]. Most studies reported a small number of neurological, pulmonary, cardiac and other complications. However, one larger study (Hughes et al 2014)[27] reported lower odds for ESG patients for neurological complications, OR of 0.48 (95%CI (0.26, 0.86), p=0.015), pulmonary complications, OR: 0.38 (95%CI (0.21, 0.67), p=0.001) and cardiac complications, OR: 0.24 (95%CI (0.15, 0.37), p<0.001). The Gore TAG study[19] reported endoleak rates for ESG patients of 8.5%.



For peer review only - http://bmjopen.bmj.com/site/about/guidelines.xhtml

*Table 2: Short-term complications*

| Study | Gore TAG | | Piffaretti et al 2007 | | Hughes et al 2011 | |
|---|---|---|---|---|---|---|
| **Arm (participants)** | **ESG (n=140)** | **OSR (n=94)** | **ESG (n=17)** | **OSR (n=11)** | **ESG (n=712)** | **OSR (n=8255)** |
| **NEUROLOGICAL** | | | | | | |
| **Paraplegia** | NR | NR | NR | NR | NR | NR |
| **Paraplegia/ Paraperisis** | 4 (3%) | 13 (14%) | NR | NR | NR | NR |
| **Cerebral Vascular Accident** | 5 (4%) | 5 (4%) | 2 (9%) | 1 (12%) | NR | NR |
| **Neurological-unspecified type** | NR | NR | NR | NR | 20 (2.8%) | 273 (3.3%) |
| **RESPIRATORY** | | | | | | |
| **Respiratory failure** | 5 (4%) | 19 (20%) | NR | NR | NR | NR |
| **Pneumonia** | NR | NR | 2 (12%) | 3 (27%) | NR | NR |
| **Pulmonary-unspecified type** | NR | NR | NR | NR | 17 (2.4%) | 462 (5.6%) |
| **CARDIAC** | | | | | | |
| **Myocardial Infarction** | 0 (0%) | 1(1%) | 0 (0%) | 1 (9%) | NR | NR |
| **Cardiac-unspecified type** | NR | NR | NR | NR | 28 (2.9%) | 1252 (15.2%) |
| **ENDOLEAKS** | | | | | | |
| **Endoleaks** | 12 (8.5%) | NR | NR | NR | NR | NR |
| **OTHER** | | | | | | |
| **Peripheral vascular disease** | 20 (14%) | 4 (4%) | NR | NR | NR | NR |



For peer review only - http://bmjopen.bmj.com/site/about/guidelines.xhtml

| | | | | | | |
|---|---|---|---|---|---|---|
| **Renal failure** | 2 (1%) | 12 (13%) | NR | NR | NR | NR |
| **Wound infection/dehiscence** | 5 (4%) | 10 (11%) | NR | NR | NR | NR |
| **GI complication** | 3 (2%) | 6 (6%) | NR | NR | NR | NR |
| **Post-implant syndrome** | NR | NR | 4(18%) | 0(0%) | NR | NR |
| *Note: Goodney et al 2011 and Von Allmen et al 2014 did not report short-term complications* | | | | | | |

For peer review only

**DISCUSSION**

This is the first systematic review that reports results from non-randomized comparative studies regarding the effectiveness of elective ESG and OSR for arch and or descending TAA. Previous reviews have either included single arm studies only[31] or were comparative reviews that included non-elective procedures[9 32], or mixed pathologies such as dissections[10 33]. The results of these reviews may not be applicable to patients undergoing elective procedures for descending or arch TAA pathology.

In this review only five comparative cohort studies were identified. The lack of RCTs comparing ESG and OSR for elective arch/descending thoracic aortic aneurysms may reflect the lack of equipoise from practitioners and patients. Well-designed non-randomized trials can demonstrate a high degree of concordance with the results of well-designed RCTs, provided that all confounders are adequately accommodated in the design and/or analysis[34]. However, the studies identified in this review had moderate to serious risk of bias, mainly due to potential for confounding.

Although, complications in the short-term also appear to favour ESG, evidence regarding short-term complications is either lacking or inconsistently reported. All five studies[19 25-28] suggested that the less invasive nature of ESG confers lower short-term mortality and fewer short-term non-fatal complications than OSR. The two small unadjusted studies[19 25] had a much larger effect than larger studies[26-28]. These larger studies used more robust methods for addressing confounding; this may



For peer review only - http://bmjopen.bmj.com/site/about/guidelines.xhtml

indicate the presence of publication bias where only smaller studies with larger effect sizes may have been published.

Longer-term results are difficult to interpret since most results fail to separate early risk from longer term survival. The two smaller studies[19 25] reported continuing survival benefit for ESG patients in the long-term but are limited by small numbers, different recruitment periods, and failure to exclude early risk. The survival advantage associated with ESG in the Gore TAG study disappears by year five, which suggests that the hazards are lower for OSR patients that survive the initial procedure. Conversely, the two larger studies (Von Allmen et al 2014 and Goodney et al 2011[26 28]) report that the short-term survival advantage of ESG are reversed by twelve months, and that the OSR group has an increasing survival advantage thereafter. Similarly, time to re-intervention favoured ESG in the smaller Gore TAG study whilst time to aortic-related re-interventions favoured OSR in the larger study Von Allmen et al 2014[28].

The evidence regarding mortality is inconsistent across the studies with more recent, larger studies[26-28] reporting a more modest survival benefit for ESG in the short-term and a survival benefit for OSR in the long-term. The two[19 25], smaller studies suggest a large reduction in the risk of mortality and short term complications in the short run and a survival benefit in the long-run for ESG.

Inconsistency of results across studies may be due to heterogeneity of the study populations, both within and across studies. Differences in recruitment periods may reflect changes in technology and practice in both groups. The larger studies [26-28] were based on patients recruited up to twenty years later than the smaller studies. Outcomes may have improved differentially with OSR patients receiving proportionally better outcomes due to general improvements in surgical practice and patient selection over time[35]. A potential reason for the reported long-term survival benefit of OSR in more recent, larger studies may be differences in patient risk in each intervention group. Open



For peer review only - http://bmjopen.bmj.com/site/about/guidelines.xhtml

surgical repair is advocated for younger, lower-risk patients, and EVAR is preferred for older, higher-risk patients[36]. This factor may not have been adequately accommodated in adjusted analyses.

The three large studies[26-28] were based on administrative health records with case ascertainment that depends on accurate coding, which can vary across and within providers[37]. Furthermore, one of the small studies, Piffaretti et al 2007[25], recruited patients from one centre which may have compromised external validity as they may for example, depend on the expertise within a centre and the nature of the patients that centre treats[30 38]. Additionally, the Gore TAG study[19] utilised a more restricted inclusion criteria for ESG patients compared to those who received OSR, potentially introducing heterogeneity between the two groups.

The headline conclusion of this review is that whilst there is a degree of variability, OSR is better for long-term survival and avoidance of re-intervention, and that ESG is better for early outcomes. This conclusion is based on non-randomised comparisons in which the influence of important confounders for which patients have ESG or OSR has not been adjusted for. Deeper exploration is required, but randomised data may be difficult to acquire as there are well-established guidelines about which patients are favoured for OSR and which for ESG. Thus, to compare ESG and OSR it is first necessary to establish an understanding of the reasons driving clinical management, for example by a structured consensus analysis such as a Delphi study. Thereafter, unless there is clinical equipoise about which patients are best served by OSR or ESG, the best quality evidence to establish the relative merits of ESG and OSR for arch/ DTA aneurysms is likely to be a systematic, prospective, observational, comparative study.

**Limitations**

The findings in this review are limited by the number and potential for confounding of the five studies[19 25-28] identified, so that definitive conclusions are not possible. The adjustment for confounders is likely to be only partial and some bias in the estimates of differences in mortality and complications may persist, despite attempts to reduce bias in large studies. Statistical analysis of the



For peer review only - http://bmjopen.bmj.com/site/about/guidelines.xhtml

non-fatal complications was not possible due to the limited and inconsistent reporting of such complications.

## CONCLUSIONS

This systematic review is the first to consider evidence from non-randomised studies that directly compare ESG and OSR for the treatment of elective arch/descending thoracic aortic aneurysms. Other reviews conducted in this area have been complicated by the inclusion of treatments for emergency cases or other thoracic aortic pathologies (e.g. thoracic aortic ulcer, or dissection).

There is limited and increasingly dated evidence addressing the comparison of ESG and OSR in the management of elective arch and or descending thoracic aortic aneurysms. Further, high quality, evidence is required. Although large RCTs may not be possible in this relatively rare condition, future studies should use strong methodological study design in order to control for potential confounders and reduce potential biases. Additionally, given the current conflicting evidence, it is important that comparisons of OSR and ESG are conducted in both the short-term and the long-term.

## FUNDING

"This report is independent research funded by the National Institute for Health Research **(Health Technology Assessment, 11/147/03 - Effective Treatments for Thoracic Aortic Aneurysms (ETT AA study): A prospective cohort study).** The views expressed in this publication are those of the author(s) and not necessarily those of the NHS, the National Institute for Health Research or the Department of Health."

## CONFLICT OF INTEREST

Andrew McCarthy, Joanne Gray, Linda Sharples, Priya. Sastry, Peter McMeekin, Andrew Cook have nothing to disclose. Luke Vale reports grants from NIHR Health Technology Assessment Programme, during the conduct of the study; and being a member of the NIHR Health Technology Assessment Programme Clinical Trials and Evaluation Panel from 2015-2018. Carol Freeman reports grants from



For peer review only - http://bmjopen.bmj.com/site/about/guidelines.xhtml

DoH NIHR HTA Programme, during the conduct of the study. Pedro Catarino reports grants from NIHR, outside the submitted work. Stephen. Large reports grants from NIHR, during the conduct of the study.

## ACKNOWLEDGMENTS

The authors would like to thank our Funders and the ETTAA Working Group for their support with this study.

## AUTHOR CONTRIBUTIONS

Author contributions according to the CrediT (contributor roles taxonomy). Conceptualization- AM, JG, PS, LS, LV, AC, PM, SL; Data curation- AM ;Formal Analysis- AM, JG, PM; Funding acquisition- JG, AC, LV, CF, PS, LS, SL; Investigation - AM, JG, PM, LV, PS; Methodology - AM, JG, PM, LV, AC, CF, PS, LS, SL; Supervision - JG, PM, LV; Validation – AM, JG, PM, PS, LS, PC, SL; Writing – original draft - AM, JG, PM; Writing – review & editing - AM, JG, PM, AC, LV, CF, PS, LS, PC, SL.

## DATA SHARING AGREEMENT

Data was extracted from the studies included in this Systematic Review and referenced below.

## PUBLIC PATIENT INVOLVEMENT

No patient involved

## REFERENCES

1. Akin I, Kische S, Rehders TC, et al. Endovascular repair of thoracic aortic aneurysm. *Archives of medical science : AMS* 2010;6(5):646-52. doi: 10.5114/aoms.2010.17075 [published Online First: 2010/10/26]
2. Sastry P, Hughes V, Hayes P, et al. The ETTAA study protocol: a UK-wide observational study of 'Effective Treatments for Thoracic Aortic Aneurysm'. *BMJ Open* 2015;5(6) doi: 10.1136/bmjopen-2015-008147
3. Kim LG, Sweeting MJ, Epstein D, et al. Optimizing Surveillance and Re-intervention Strategy Following Elective Endovascular Repair of Abdominal Aortic Aneurysms. *Annals of Surgery* 2019;Publish Ahead of Print doi: 10.1097/sla.0000000000003625
4. Patel R, Sweeting MJ, Powell JT, et al. Endovascular versus open repair of abdominal aortic aneurysm in 15-years' follow-up of the UK endovascular aneurysm repair trial 1 (EVAR trial 1): a randomised controlled trial. *The Lancet* 2016;388(10058):2366-74. doi: 10.1016/S0140-6736(16)31135-7



For peer review only - http://bmjopen.bmj.com/site/about/guidelines.xhtml

5. Ricotta JJ. Endoleak management and postoperative surveillance following endovascular repair of thoracic aortic aneurysms. *Journal of Vascular Surgery* 2010;52(4, Supplement):91S-99S. doi: https://doi.org/10.1016/j.jvs.2010.06.149
6. Kalteis M, Haller F, Artmann A, et al. Experience and Outcomes After a Decade of Endovascular Abdominal Aortic Aneurysm Repair: A Retrospective Study From a Community-Based Single Center. *Annals of Vascular Surgery* 2012;26(3):330-37. doi: https://doi.org/10.1016/j.avsg.2011.06.012
7. Mertens J, Houthoofd S, Daenens K, et al. Long-term results after endovascular abdominal aortic aneurysm repair using the Cook Zenith endograft. *Journal of Vascular Surgery* 2011;54(1):48-57.e2. doi: https://doi.org/10.1016/j.jvs.2010.12.068
8. Abraha I, Romagnoli C, Montedori A, et al. Thoracic stent graft versus surgery for thoracic aneurysm. *Cochrane Database of Systematic Reviews* 2016(6) doi: 10.1002/14651858.CD006796.pub4
9. Health Quality O. Endovascular Repair of Descending Thoracic Aortic Aneurysm: An Evidence-Based Analysis. *Ontario Health Technology Assessment Series* 2005;5(18):1-59.
10. Walsh SR, Tang TY, Sadat U, et al. Endovascular stenting versus open surgery for thoracic aortic disease: Systematic review and meta-analysis of perioperative results. *Journal of Vascular Surgery* 2008;47(5):1094-98.e3. doi: https://doi.org/10.1016/j.jvs.2007.09.062
11. Gray J, McCarthy A, McMeekin P, et al. Effective Treatments for Thoracic Aortic Aneurysms (ETTAA study): protocol for a systematic review. *PROSPERO* 2016(CRD420170545565)
12. Reeves BC, Deeks JJ, Higgins JP, et al. Including Non-Randomized Studies. In: Higgins JP, Green S, eds. Cochrane Handbook for Systematic Reviews of Interventions2008.
13. Deeks J, Dinnes J, D'Amico R, et al. Evaluating non-randomised intervention studies. *Health Technology Assessment* 2003;7(27)
14. Texas Uo. Search Filters for Various Databases: PubMed 2016 [updated 3 October 2017. Available from: https://libguides.sph.uth.tmc.edu/search_filters accessed 14 July 2016.
15. Dake MD, Miller DC, Semba CP, et al. Transluminal Placement of Endovascular Stent-Grafts for the Treatment of Descending Thoracic Aortic Aneurysms. *New England Journal of Medicine* 1994;331(26):1729-34. doi: 10.1056/nejm199412293312601
16. Higgins JP, Green S. Guide to the Contents of a Cochrane Protocol and Review. Cochrane Handbook for Systematic Reviews of Interventions2008.
17. Sterne JA, Hernán MA, Reeves BC, et al. ROBINS-I: a tool for assessing risk of bias in non-randomised studies of interventions. *BMJ* 2016;355 doi: 10.1136/bmj.i4919
18. Review Manager (RevMan) [program]. Version 5.3 version. The Nordic Cochrane Centre: The Cochrane Collaboration, 2014.
19. Bavaria JE, Appoo JJ, Makaroun MS, et al. Endovascular stent grafting versus open surgical repair of descending thoracic aortic aneurysms in low-risk patients: a multicenter comparative trial. *Journal of Thoracic & Cardiovascular Surgery* 2007;133(2):369-77.
20. Cho JS, Haider SE, Makaroun MS. Endovascular therapy of thoracic aneurysms: Gore TAG trial results. *Seminars in Vascular Surgery* 2006;19(1):18-24.
21. Cho JS, Haider SE, Makaroun MS. US multicenter trials of endoprostheses for the endovascular treatment of descending thoracic aneurysms. *Journal of Vascular Surgery* 2006;43 Suppl A:12A-19A.
22. Go MR, Cho J-S, Makaroun MS. Mid-term Results of a Multicenter Study of Thoracic Endovascular Aneurysm Repair Versus Open Repair. *Perspectives in Vascular Surgery and Endovascular Therapy* 2007;19(2):124-30. doi: 10.1177/1531003507303430
23. Makaroun MS, Dillavou ED, Wheatley GH, et al. Five-year results of endovascular treatment with the Gore TAG device compared with open repair of thoracic aortic aneurysms. *Journal of Vascular Surgery* 2008;47(5):912-8.
24. Dillavou ED, Makaroun MS. Predictors of morbidity and mortality with endovascular and open thoracic aneurysm repair. *Journal of Vascular Surgery* 2008;48(5):1114-9; discussion 19-20.

For peer review only



For peer review only - http://bmjopen.bmj.com/site/about/guidelines.xhtml

25. Piffaretti G, Tozzi M, Lomazzi C, et al. Endovascular repair versus conventional surgery for descending thoracic aortic aneurysms. *Italian Journal of Vascular and Endovascular Surgery* 2007;14(4):279-85.
26. Goodney PP, Travis L, Lucas FL, et al. Survival after open versus endovascular thoracic aortic aneurysm repair in an observational study of the Medicare population. *Circulation* 2011;124(24):2661-9.
27. Hughes K, Guerrier J, Obirieze A, et al. Open versus endovascular repair of thoracic aortic aneurysms: a Nationwide Inpatient Sample study. *Vascular & Endovascular Surgery* 2014;48(5-6):383-7.
28. Von Allmen RS, Anjum A, Powell JT. Outcomes after endovascular or open repair for degenerative descending thoracic aortic aneurysm using linked hospital data. *British Journal of Surgery* 2014;101(10):1244-51.
29. Austin PC. An Introduction to Propensity Score Methods for Reducing the Effects of Confounding in Observational Studies. *Multivariate Behav Res* 2011;46(3):399-424. doi: 10.1080/00273171.2011.568786 [published Online First: 2011/06/08]
30. Bellomo R, Warrillow SJ, Reade MC. Why we should be wary of single-center trials. *Critical Care Medicine* 2009;37(12):3114-19. doi: 10.1097/CCM.0b013e3181bc7bd5
31. Eggebrecht H, Nienaber CA, Neuhäuser M, et al. Endovascular stent–graft placement in aortic dissection: a meta-analysis. *European Heart Journal* 2005;27(4):489-98. doi: 10.1093/eurheartj/ehi493
32. Alsawas M, Zaiem F, Larrea-Mantilla L, et al. Effectiveness of surgical interventions for thoracic aortic aneurysms: A systematic review and meta-analysis. *Journal of Vascular Surgery* 2017;66(4):1258-68.e8. doi: https://doi.org/10.1016/j.jvs.2017.05.082
33. Cheng D, Martin J, Shennib H, et al. Endovascular Aortic Repair Versus Open Surgical Repair for Descending Thoracic Aortic Disease: A Systematic Review and Meta-Analysis of Comparative Studies. *Journal of the American College of Cardiology* 2010;55(10):986-1001. doi: https://doi.org/10.1016/j.jacc.2009.11.047
34. Marko NF, Weil RJ. The Role of Observational Investigations in Comparative Effectiveness Research. *Value in Health* 2010;13(8):989-97. doi: https://doi.org/10.1111/j.1524-4733.2010.00786.x
35. Ludman Peter F, Moat N, de Belder Mark A, et al. Transcatheter Aortic Valve Implantation in the United Kingdom. *Circulation* 2015;131(13):1181-90. doi: 10.1161/CIRCULATIONAHA.114.013947
36. Katzen BT, Dake MD, MacLean AA, et al. Endovascular Repair of Abdominal and Thoracic Aortic Aneurysms. *Circulation* 2005;112(11):1663-75. doi: doi:10.1161/CIRCULATIONAHA.105.541284
37. Prasad A, Helder MR, Brown DA, et al. Understanding Differences in Administrative and Audited Patient Data in Cardiac Surgery: Comparison of the University HealthSystem Consortium and Society of Thoracic Surgeons Databases. *Journal of the American College of Surgeons* 2016;223(4):551-57.e4. doi: https://doi.org/10.1016/j.jamcollsurg.2016.06.393
38. Unverzagt S, Prondzinsky R, Peinemann F. Single-center trials tend to provide larger treatment effects than multicenter trials: a systematic review. *Journal of Clinical Epidemiology* 2013;66(11):1271-80. doi: 10.1016/j.jclinepi.2013.05.016

For peer review only



For peer review only - http://bmjopen.bmj.com/site/about/guidelines.xhtml

*Figure 1: PRISMA Diagram*

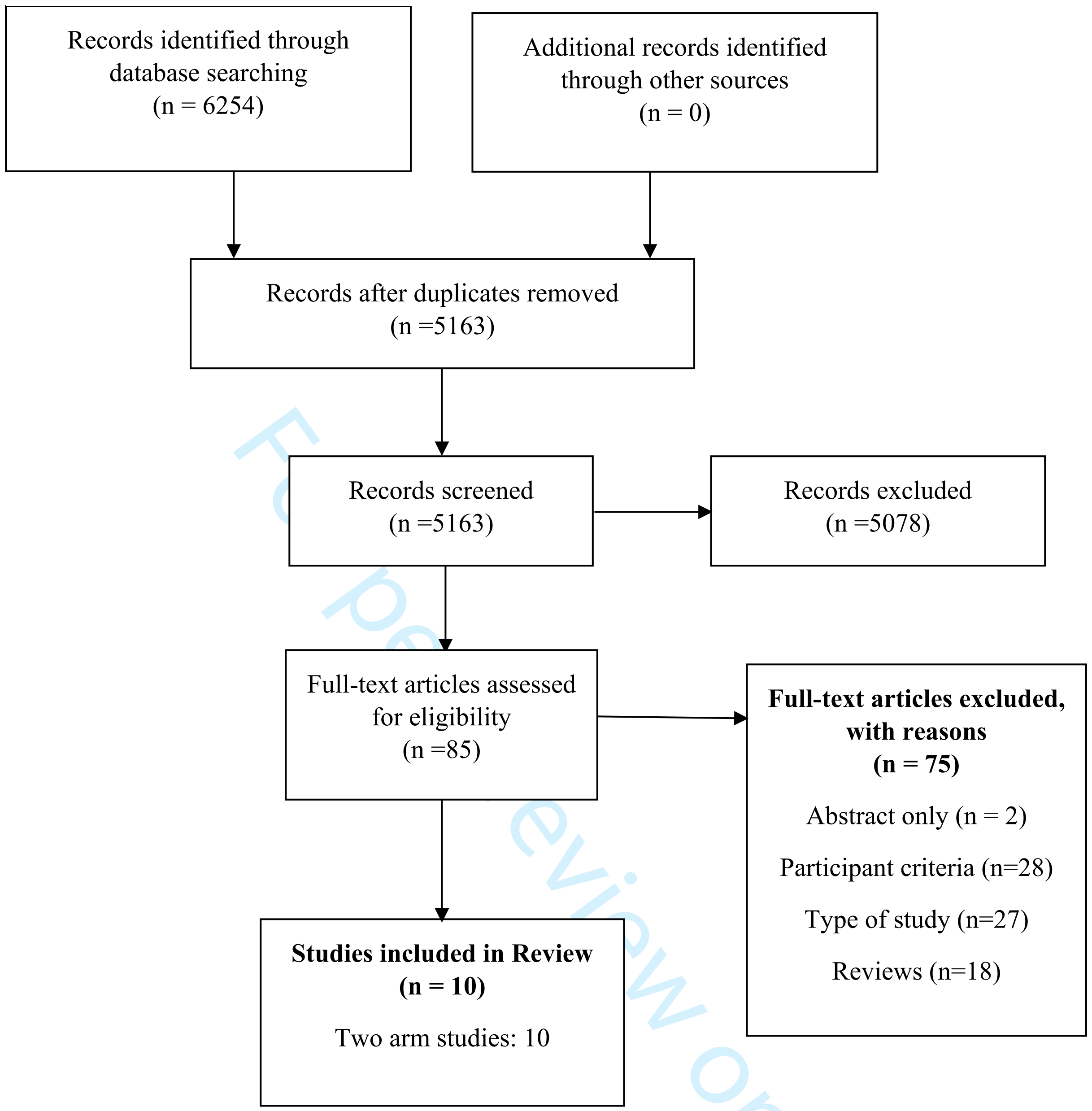

*Figure 2: Forest plot of unadjusted short-term all-cause mortality*

| Study or Subgroup | ESG Events | ESG Total | OSR Events | OSR Total | Weight | Odds Ratio M-H, Random, 95% CI |
|---|---|---|---|---|---|---|
| **1.1.1 Subgroup 1** | | | | | | |
| Goodney 2011 | 167 | 2732 | 893 | 12573 | 47.7% | 0.85 [0.72, 1.01] |
| Hughes 2014 | 26 | 712 | 380 | 8255 | 28.6% | 0.79 [0.52, 1.18] |
| Von Allmen 2014 | 23 | 354 | 20 | 264 | 17.2% | 0.85 [0.46, 1.58] |
| **Subtotal (95% CI)** | | **3798** | | **21092** | **93.4%** | **0.84 [0.72, 0.98]** |
| Total events | 216 | | 1293 | | | |
| Heterogeneity: Tau² = 0.00; Chi² = 0.13, df = 2 (P = 0.94); I² = 0% | | | | | | |
| Test for overall effect: Z = 2.22 (P = 0.03) | | | | | | |
| **1.1.2 Subgroup 2** | | | | | | |
| Gore TAG | 3 | 140 | 11 | 94 | 5.1% | 0.17 [0.04, 0.61] |
| Piffaretti 2007 | 1 | 17 | 2 | 11 | 1.5% | 0.28 [0.02, 3.55] |
| **Subtotal (95% CI)** | | **157** | | **105** | **6.6%** | **0.18 [0.06, 0.59]** |
| Total events | 4 | | 13 | | | |
| Heterogeneity: Tau² = 0.00; Chi² = 0.13, df = 1 (P = 0.71); I² = 0% | | | | | | |
| Test for overall effect: Z = 2.85 (P = 0.004) | | | | | | |
| **Total (95% CI)** | | **3955** | | **21197** | **100.0%** | **0.75 [0.55, 1.03]** |
| Total events | 220 | | 1306 | | | |
| Heterogeneity: Tau² = 0.04; Chi² = 6.72, df = 4 (P = 0.15); I² = 40% | | | | | | |
| Test for overall effect: Z = 1.80 (P = 0.07) | | | | | | |
| Test for subgroup differences: Chi² = 6.45, df = 1 (P = 0.01), I² = 84.5% | | | | | | |

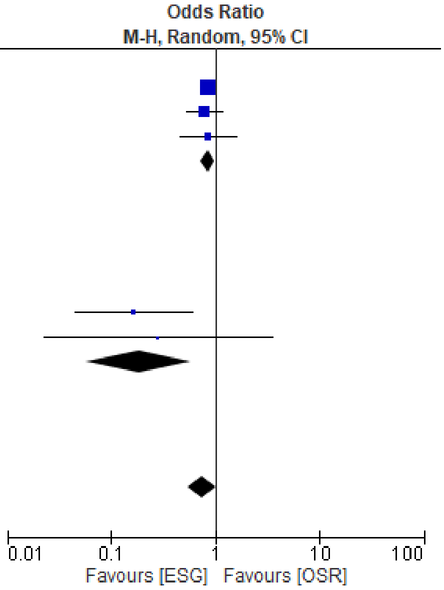


For peer review only

*Figure 3: Forest plot of adjusted short-term all-cause mortality*

| Study or Subgroup | log[Odds Ratio] | SE | Weight | Odds Ratio IV, Random, 95% CI |
|---|---|---|---|---|
| Goodney 2011 | -0.08 | 0.3 | 31.7% | 0.92 [0.51, 1.66] |
| Hughes 2014 | -0.62 | 0.26 | 42.2% | 0.54 [0.32, 0.90] |
| Von Allmen 2014 | -0.24 | 0.33 | 26.2% | 0.79 [0.41, 1.50] |
| **Total (95% CI)** | | | **100.0%** | **0.71 [0.51, 0.98]** |

Heterogeneity: Tau² = 0.00; Chi² = 2.00, df = 2 (P = 0.37); I² = 0%
Test for overall effect: Z = 2.07 (P = 0.04)

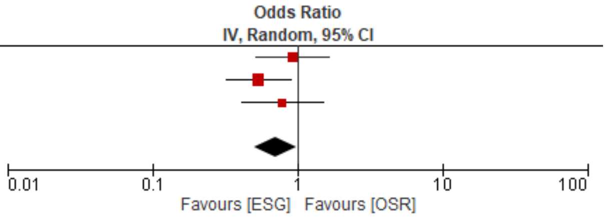


peer review only

**Appendix 1: Search Strategy**

**Medline/Central/HTA**

1. (TEVAR or EVAR).ti,ab.
2. (endovascular adj1 (stent* or repair* or treat* or surg* or (aneurysm? adj1 repair*))).ti,ab.
3. (open adj2 (surg* or repair*)).ti,ab.
4. endoprosthe*.mp.
5. *Aortic Aneurysm, Thoracic/su [Surgery]
6. *Aortic Thoracic/su [Surgery]
7. Thoracic Surgical Procedures/
8. or/1-7
9. exp Aortic Aneurysm, Thoracic/
10. (thorac* adj2 aneurysm*).ti,ab.
11. (descending adj1 aort* adj1 aneurysm*).ti,ab
12. TAA*.ti,ab.
13. (arch adj3 aneurysm*).ti.ab.
14. or/9-13
15. morbid*.mp.
16. mortalit*.mp.
17. outcome?.mp.
18. complication*.mp.
19. (rupture? or endoleak?).mp.
20. device migration.mp.
21. (ae or co or de).fs

For peer review only

22. (safe or safety or side effect* or undesirable effect* or treatment emergent or tolerability or toxicity or adrs or (adverse adj2 (effect or effects or reaction or reactions or event or events to outcome or outcomes))).ti,ab.
23. ((technical or surg*) adj2 (advance* or improve*)).mp.
24. (quality adj2 life).mp.
25. Mortality/
26. Mortbidity/
27. Postoperative complications/
28. Intraoperative complications/
29. Aortic Aneurysm, Thoracic/co
30. Conversion to Open Surgery/
31. “Quality of Life”
32. or/15-31
33. 8 and 14 and 32
34. (“clinical trial” or “clinical trial, phase I” or “clinical trial, phase ii” or clinical trial phase iii or clinical trial, phase iv or controlled clinical trial or “multicentre study” or “randomized controlled trial”).pt. or double-blind method/ or clinical trials, phase iii as topic/ or clinical trials, phase iv as topic/ or controlled clinical trials as topic/ or randomized controlled trials as topic/ or early termination of clincal trials as topic/ or multicenter studies as topic/ or ((randomi?ed adj7 trial*) or (controlled adj3 trial*) or (clinical adj2 trial*) or ((single or doubl* or tripl* or treb*) and (blind* or mask*))).ti,ab,kw. or (“4 arm” or “four arm”).ti,ab,kw.
35. cohort studies/ or longitudinal studies/ or follow-up studies/ or prospective studies/ or retrospective studies/ or cohort.ti,ab. or longitudinal.ti,ab. or prospective.ti,ab. or retrospective.ti,ab. or quasi-experiment.ti,ab.

For peer review only

36. Clinical Trial.pt. or random*.tw. or Clinical Trials/ or clinical trial.tw. or Follow-up Studies/ or prospectiv*.tw. or control*.tw.
37. or/34-36
38. 33 and 37

EMBASE

1. (TEVAR or EVAR).ti,ab.
2. (endovascular adj1 (stent* or repair* or treat* or surg* or (aneurysm? adj1 repair*))).ti,ab.
3. (open adj2 (surg* or repair*)).ti,ab.
4. endoprosthe*.mp.
5. Thoracic Aorta Aneruysm/su [Surgery]
6. Thoracoabdominal aorta aneurysm/su [Surgery]
7. Thoracic Aorta/su [Surgery]
8. exp Thoracic aorta surgery/
9. or/1-8
10. exp Thoracic Aorta Aneurysm/
11. exp Thoracoabdominal aorta aneurysm/
12. Thoracic Aorta/
13. (thorac* adj2 aneurysm*).ti,ab.
14. (descending adj1 aort* adj1 aneurysm*).ti,ab.
15. TAA*.ti,ab.
16. (arch adj3 aneurysm*).ti,ab.
17. or/10-16
18. (outcome? or mortalit*).mp.
19. complication*.mp.
20. ((technical or surg*) adj2 (advance* or improve*)).mp.

For peer review only

21. (quality adj2 life).mp.
22. Mortality/
23. Postoperative complication/
24. peroperative complication/
25. Thoracic Aorta Aneurysm/co
26. “Quality of Life”/
27. or/18-26
28. (Randomised Controlled Trial or Pragmatic Clinical Trial).pt or exp Randomized Controlled Trials as Topic/ or “Randomized Controlled Trial (topic)”/ or Randomized Controlled Trial/ or Randomization/ or Random Allocation/ or Double-Blind Method/ or Double Blind Procedure/ or Double-Blind Studies/ or Single-Blind Method/ or Single Blind Procedure/ or Single-Blind Studies/ or Placebos/ or Placebo/ or (random* or sham or placebo*).ti,ab,hw,kf,kw. or ((singl* or doubl*) adj (blind* or dumm* or mask*)).ti,ab,hw,,kw. or ((tripl* or trebl*) adj (blind* or dumm* or mask*)).ti,ab,hw,,kw.
29. random:.tw. or placebo:.mp. or double-blind:.mp.
30. controlled clinical trial/ or clinical trial/ or exp controlled study/ or randomized controlled trial/ or exp quasi experimental study/
31. or/28-30
32. 9 and 17 and 27 and 31
33. limit 32 to (human and yr=”1994-Current”)
34. exp cohort analysis/ or exp longitudinal study/ or exp prospective study/ or exp follow up/ or cohort*.tw.
35. limit 34 to (human and yr=”1994-Current”)
36. 9 and 17 and 27 and 35
37. 36 not 33
38. 33 or 37

For peer review only

**Appendix 2: Data extraction table**

| Study | **Von Allmen et al 2014** | **Goodney et al 2011** | **Goodney et al 2011 (propensity matched cohort)** | **Gore TAG** | **Hughes et al 2014** | **Piffaretti et al 2007** |
|---|---|---|---|---|---|---|
| **METHODS** | | | | | | |
| **Study Design** | Comparative cohort | Comparative cohort | Comparative cohort | Comparative cohort | comparative cohort | Comparative cohort |
| **Method of matching** | None | None | Stratified propensity score | ESG patients judged eligible for OSR | None | OSR patients judged eligible for ESG |
| **Inclusion Criteria** | Hospital diagnosis code 171.2 (TAA without mention of | | | Fusiform descending thoracic aortic aneurysm at least twice the size of the | Nationwide inpatient Sample database of | Elective, Chronic fusiform or saccular aneurysm of the |

For peer review only

| | | | | | | |
|---|---|---|---|---|---|---|
| | rupture) from ICD-10 score linked to operations code according to OPCS-4, linked with all-cause mortality from ONS and ICD-10, only OPCS-4 codes: L18.2, L19.2, L20.2, L21.2 | | | normal adjacent aorta or saccular aneurysm, Life expectancy >2 years<br>Surgical candidate<br>Male or infertile female >21 years old<br>Descending aorta must be clampable distal to the left carotid artery, and distal anastomosis must be performed proximal to the celiac axis<br>Inner aortic diameter of 23-37mm adjacent to aneurysm | patients >18 years old who had undergone an open or endovascular repair of non-ruptured thoracic aortic aneurysm. Patients selected using ICD-9 procedures codes 39.73 and 38.45. | descending thoracic aorta, OSR patients would have been a candidates for ESG due to morphological criteria, Selected on surgeon's opinion of lower risk of ESG |

| | | | | | | |
|---|---|---|---|---|---|---|
| | | | | Lack of significant thrombus of calcification in landing zones<br>Minimum of 2cm of normal thoracic aorta proximal and distal to aneurysm<br>Aortic taper of no more than 4mm or ability to treat with more than one graft | | |
| **Exclusion Criteria** | Dissection, Abdominal aneurysms, Non-identified area aneurysm, Ascending | | | Mycotic aneurysm, Hemodynamically unstable ruptured aneurysm , Major operation (other than planned subclavian to carotid transposition or | NA | Traumatic, Mycotic thoracic aneurysms, Dissections, Aneurysms of the ascending aorta, Aneurysms of the |

For peer review only

| | | | | | | |
|---|---|---|---|---|---|---|
| | aneurysm, Suprarenal aneurysm, Non-aortic procedures, age <50yrs | | | bypass) within 30 days, MI or CVA within 6 weeks, Creatinine >2.0mg/dL, Connective tissue disorder, Acute or chronic aortic dissection, Planned occlusion of carotid or celiac arteries, Documented drug abuse within 6 months, Participation in another investigational device or drug study within 1 year | | aortic arch, Aortic ulcers |
| **Definition of the short-term** | 30 days post-procedure | latest occurring of discharge or | latest occurring of discharge or | latest occurring of discharge or 30 days post-intervention | post-procedure hospital stay | 30-days post-intervention |

For peer review only

| | | | | | | |
|---|---|---|---|---|---|---|
| | | 30 days post-intervention | 30 days post-intervention | | | |
| **PARTICIPANTS** | | | | | | |
| **Country** | UK | USA | USA | USA | USA | Italy |
| **Setting** | Multi-centre – Hospital episode statistics | Multi-centre – Medicare data | Multi-centre – Medicare data | Multi-centre | Multi-centre | Single-centre |
| **Time frame** | 2006 to 2011 | 1998-2007 | 2003-2007 | ESG 1999- 2001, OSR pre 1999 (n=44) & 1999-2001 (n=50) | 1998-2007 | ESG 2000-2007, OSR 1996-2000 |
| **Recruitment** | Total 618<br>ESG 354<br>OSR 264 | Total 13,998<br>ESG 2433<br>OSR 11,565 | Total 1100<br>ESG 550<br>OSR 550 | Total 234<br>ESG 140<br>OSR 94 | Total 8967<br>ESG 712<br>OSR 8255 | Total 28<br>ESG 17<br>OSR 11 |
| **Age summary** | median (IQR)<br>ESG 73(66,78), OSR 71 (63-76) | mean (95% C.I.) | mean (95% C.I.) | mean(SD)<br>ESG 75.9 (10.4), OSR 68.2 (10.2) | median (IQR)<br>ESG 72 (63- 78), OSR 63 (52, 72) | mean (SD)<br>ESG 66 (10), OSR 61 (13) |

For peer review only

| | | | | | | |
|---|---|---|---|---|---|---|
| | | ESG 75.9 (75.6, 76.1), OSR 73.8 (73.7, 73.9) | ESG 71.1 (70.8, 71.44), OSR 70.7 (70.7, 71.1) | | | |
| **Proportion male** | ESG 232 (65.5%), OSR 137 (51.9%) | % (95% C.I.): ESG 58.7 (56.7, 60.7), OSR 55.4 (54.8, 56.3) | % (95% C.I.): ESG 64.0 (59.9, 68.0), OSR 68.1% (64.2, 72.0) | ESG 90(57%), OSR 48 (51%) | 5415 (61.3%) ESG, 437 (65.6%) OSR | ESG 14 (82%), OSR 8 (73%) |
| **Black race** | NR | % (95% C.I.): ESG 7.5 (6.4, 8.5), OSR 3.8 (3.4, 4.5) (p=0.001) | % (95% C.I.): ESG 2.7 (1.4, 4.1), OSR 2.7 (1.4, 4.0) (p=0.99) | NR | NR | NR |
| **Charlson comorbidity score** | NR | % (95% C.I.): ESG 1.46 | n(95% C.I.): ESG 0.5 (0.41, | NR | NR | NR |

For peer review only

| | | | | | | |
|---|---|---|---|---|---|---|
| | | (1.38, 1.54), OSR 0.83 (0.80, 0.86) (p=0.00001) | 0.60), OSR 0.44 (0.35, 0.53) (p=0.34) | | | |
| **Diabetes** | NR | % (95% C.I.): ESG 8.00 (6.7, 8.8), OSR 4.10 (3.7, 4.5) (p=0.001) | % (95% C.I.): ESG 4.90 (3.0, 6.7), OSR 2.90 (1.5, 4.3) (p=0.09) | NR | 684 (13.2%) ESG, 94 (8.3%) OSR (p<0.001) | NR |
| **Myocardial infarction** | NR | % (95% C.I.): ESG 7.00 (6.00, 8.1), OSR 5.10 (4.6, 5.4) (p=0.001) | % (95% C.I.): ESG 2.50 (1.2, 3.8), OSR 3.40 (1.9, 4.9) (p=0.38) | -NR | NR | NR |
| **Congestive heart failure** | NR | % (95% C.I.): ESG 10.85% | % (95% C.I.): ESG 3.00 (1.6, | ESG 13 (9%), OSR 9 (10%) (p=1.00) | NR | NR |

| | | | | | | |
|---|---|---|---|---|---|---|
| | | (9.6%- 12.0%), OSR 10.60% (10.1%- 11.2%) (p=0.16) | 4.5), OSR 4.70 (2.9, 6.5) (p=0.16) | | | |
| **Cerebrovascular disease** | NR | % (95% C.I.): ESG 5.09% (4.2%-5.9%), OSR 3.20% (2.9%- 3.5%) (p=0.65) | % (95% C.I.): ESG 2.00 (0.5, 2.6), OSR 2.00 (0.8, 3.1) (p=0.65) | NR | NR | 4 (23%) ESG, 1 (9%) OSR (p=NS) |
| **COPD** | NR | % (95% C.I.): ESG 22.04% (20.3%- 23.6%), OSR | % (95% C.I.): ESG 4.00 (2.3, 5.6), OSR 4.90 | ESG 56 (40%), OSR 36 (38%) (p=0.89) | 247 (34.7%) ESG, 1483 (17.9%) (P<0.001) | 10 (59%) ESG, 6 (54%) OSR (p=NS) |

For peer review only

| | | | | | | |
|---|---|---|---|---|---|---|
| | | 10.65% (10.0%-11.2%) (p=0.47) | (3.0, 6.7) (p=0.47) | | | |
| **Chronic renal failure** | NR | % (95% C.I.): ESG 4.9% (4.0%- 5.7%), OSR 0.88% (0.07%- 0.10%) (p=0.70) | % (95% C.I.): ESG 0.01 (0, 1.4), OSR 0.50 (0, 1.1) (p=0.70) | NR | 90 (12.6%) ESG, 126 (1.5%) OSR (p<0.001) | 4 (23%) ESG, 1 (9%) OSR (p=NS) |
| **History of malignancy** | NR | % (95% C.I.): ESG 5.71% (4.7%-6.6%), OSR 2.91% (2.6%- 3.2%) (p=0.82) | % (95% C.I.): ESG 1.80 (0.6, 2.9), OSR 1.60 (0.5, 2.6) (p=0.82) | NR | NR | NR |

For peer review only

| | | | | | | |
|---|---|---|---|---|---|---|
| **History of smoking (current or past)** | NR | NR | NR | ESG 117 (84%), OSR 77 (82%) (p=0.86) | NR | NR |
| **BMI (kg/m2)** | NR | NR | NR | mean (SD): ESG 26.4 (4.7), OSR 26.9 (5) (p=0.44) | NR | NR |
| **Coronary artery disease** | NR | NR | NR | ESG 69 (49%), OSR 34 (36%) (p=0.60) | NR | NR |
| **Stroke** | NR | NR | NR | ESG 14 (10%), OSR 9 (9%) (p=1.00) | NR | NR |
| **Peripheral arterial disease** | NR | NR | NR | ESG 22 (16%), OSR 10 (11%) (p=0.33) | NR | NR |
| **Symptomatic aneurysm** | NR | NR | NR | ESG 30 (21%), OSR 36 (38%) (p=0.007) | NR | NR |
| **Other concomitant aneurysm:** | NR | NR | NR | ESG 39 (28%), OSR 26 (28%) (p=1.00) | NR | NR |
| **Renal dialysis** | NR | NR | NR | ESG 2 (1%), OSR 0 (0%) (p=0.52) | NR | NR |

For peer review only

| | | | | | | |
|---|---|---|---|---|---|---|
| **Paraplegia** | NR | NR | NR | ESG 1 (1%), OSR 0 (0%) (p=1.00) | NR | NR |
| **Cancer** | NR | NR | NR | ESG 27 (19%), OSR 12 (13%) (p=0.21) | NR | NR |
| **Prior major vascular surgery** | NR | NR | NR | ESG 63 (45%), OSR 52 (55%) (p=0.14) | NR | NR |
| **Cardiac comorbidities** | NR | NR | NR | NR | 265 (37.2%) ESG, 3535 (42.8%) (p<0.001) | NR |
| **Hypertension** | NR | NR | NR | NR | NR | 16 (94%) ESG, 10 (91%) OSR (p=NS) |
| **Peripheral obstructive disease** | NR | NR | NR | NR | NR | 5 (29%) ESG, 2 (18%) OSR (p=NS) |
| **Ischemic heart disease** | NR | NR | NR | NR | NR | 6 (35%) ESG, 5 (45%) OSR (p=NS) |

For peer review only

| | | | | | | |
|---|---|---|---|---|---|---|
| **Cardiac Surgery** | NR | NR | NR | NR | NR | (18%) ESG, 0 (0%) OSR (p=NS) |
| **SHORT- TERM OUTCOMES** | | | | | | |
| **All-cause mortality** | ESG 21 (5.9%) OSR 4 (1.5%) Adjusted OR: 0.79 | ESG (6.1%) 95%CI 5.1%, 7.0%) OSR 7.1% (94%CI 6.7%, 7.6%) | ESG 4.2% (95% C.I. 2.5%-5.8%) OSR 4.5% (95% C.I. 2.8%-6.2%) | ESG 3 (2.1%) OSR 11 (11.7%) Adjusted OR: 0.54 | ESG 25 OSR 379 | ESG 1 (6%) OSR 2 (20%) |
| **Re-intervention** | ESG 21 (5.9%) OSR 4 (1.5%) Adjusted OR: 2.97 (1.09, 8.10) | NR | NR | NR | NR | NR |
| **Paraplegia/ Paraperisis** | NR | NR | NR | ESG 4 (3%) OSR 13 (14%) | NR | NR |

For peer review only

| | | | | | | |
|---|---|---|---|---|---|---|
| **Cerebral Vascular Accident** | NR | NR | NR | ESG 5 (4%)<br>OSR 5 (4%) | NR | ESG 2 (9%)<br>OSR 1 (12%) |
| **Neurological-unspecified type** | NR | NR | NR | NR | ESG 20 (2.8%)<br>OSR 273 (3.3%)<br>Adjusted OR: 0.38 | NR |
| **Respiratory failure** | NR | NR | NR | ESG 5 (4%)<br>OSR 19 (20%) | NR | NR |
| **Pneumonia** | NR | NR | NR | NR | NR | ESG 2 (12%)<br>OSR 3 (27%) |
| **Pulmonary-unspecified type** | NR | NR | NR | NR | ESG 17 (2.4%)<br>OSR 462 (5.6%)<br>Adjusted OR: 0.48 | NR |
| **Myocardial Infarction** | NR | NR | NR | ESG 0 (0%)<br>OSR 1(1%) | NR | ESG 0 (0%)<br>OSR 1 (9%) |
| **Cardiac-unspecified type** | NR | NR | NR | NR | ESG 28 (2.9%)<br>OSR 1252 (15.2%) | NR |

For peer review only

| | | | | | | |
|---|---|---|---|---|---|---|
| | | | | | Adjusted OR: 0.24 | |
| **Peripheral vascular disease** | NR | NR | NR | ESG 20 (14%)<br>OSR 4 (4%) | NR | NR |
| **Renal failure** | NR | NR | NR | ESG 2 (1%)<br>OSR 12 (13%) | NR | NR |
| **Wound infection/dehiscence** | NR | NR | NR | ESG 5 (4%)<br>OSR 10 (11%) | NR | NR |
| **GI complication** | NR | NR | NR | ESG 3 (2%)<br>OSR 6 (6%) | NR | NR |
| **Post-implant syndrome** | NR | NR | NR | NR | NR | ESG 4(18%)<br>OSR 0(0%) |
| **LONG-TERM OUTCOMES** | | | | | | |
| All-cause survival | 1 year adjusted OR: 1.1 95%CI [0.70-1.73], p=0.667 | NR | NR | NR | NR | NR |

For peer review only - http://bmjopen.bmj.com/site/about/guidelines.xhtml

| | | | | | | |
|---|---|---|---|---|---|---|
| | 5 year adjusted HR: 1.45 95%CI [1.08-1.94], p=0.013 | | | | | |
| **aortic-related survival** | 5 year adjusted HR: 1.27 95%CI [0.81-1.98], p=0.298 | NR | NR | NR | NR | NR |
| **survival from aortic-related re-intervention** | 5 year adjusted HR: 1.70 95%CI [1.11-2.60], p=0.014 | NR | NR | NR | NR | NR |

For peer review only

**Appendix 3: Risk of Bias assessment**

*Risk of Bias Results using the ROBINS-I risk of bias tool*

| Domain – source of bias | Goodney et al 2011 | Goodney et al 2011 (Propensity matched) | Gore TAG | Hughes et al 2014 | Piffaretti et al 2007 | Von Allmen et al 2014 |
|---|---|---|---|---|---|---|
| **Confounding** | Severe | Moderate | Severe | Severe | Severe | Severe |
| **Selection of participants into the study** | Low | Low | Low | Low | Low | Low |
| **Classification of interventions** | Low | Low | Low | Low | Low | Low |
| **Deviations from intended interventions** | Low | Low | Low | Low | Low | Low |
| **Missing data** | Low | Low | Low | Low | Low | Low |
| **Measurement of outcomes** | Low | Low | Low | Low | Low | Low |
| **Bias in selection of the reported result** | Moderate | Moderate | Moderate | Moderate | Moderate | Moderate |
| **Overall** | Severe | Moderate | Severe | Severe | Severe | Severe |

For peer review only

| Section/topic | # | Checklist item | Reported on page # |
|---|---|---|---|
| **TITLE** | | | |
| Title | 1 | Identify the report as a systematic review, meta-analysis, or both. | 1 |
| **ABSTRACT** | | | |
| Structured summary | 2 | Provide a structured summary including, as applicable: background; objectives; data sources; study eligibility criteria, participants, and interventions; study appraisal and synthesis methods; results; limitations; conclusions and implications of key findings; systematic review registration number. | 2 |
| **INTRODUCTION** | | | |
| Rationale | 3 | Describe the rationale for the review in the context of what is already known. | 4 |
| Objectives | 4 | Provide an explicit statement of questions being addressed with reference to participants, interventions, comparisons, outcomes, and study design (PICOS). | 5 |
| **METHODS** | | | |
| Protocol and registration | 5 | Indicate if a review protocol exists, if and where it can be accessed (e.g., Web address), and, if available, provide registration information including registration number. | 6 |
| Eligibility criteria | 6 | Specify study characteristics (e.g., PICOS, length of follow-up) and report characteristics (e.g., years considered, language, publication status) used as criteria for eligibility, giving rationale. | 6 |
| Information sources | 7 | Describe all information sources (e.g., databases with dates of coverage, contact with study authors to identify additional studies) in the search and date last searched. | 7 |
| Search | 8 | Present full electronic search strategy for at least one database, including any limits used, such that it could be repeated. | 7 |
| Study selection | 9 | State the process for selecting studies (i.e., screening, eligibility, included in systematic review, and, if applicable, included in the meta-analysis). | 8 |
| Data collection process | 10 | Describe method of data extraction from reports (e.g., piloted forms, independently, in duplicate) and any processes for obtaining and confirming data from investigators. | 8 |

For peer review only

| | | | |
|---|---|---|---|
| Data items | 11 | List and define all variables for which data were sought (e.g., PICOS, funding sources) and any assumptions and simplifications made. | 8 |
| Risk of bias in individual studies | 12 | Describe methods used for assessing risk of bias of individual studies (including specification of whether this was done at the study or outcome level), and how this information is to be used in any data synthesis. | 8 |
| Summary measures | 13 | State the principal summary measures (e.g., risk ratio, difference in means). | 9 |
| Synthesis of results | 14 | Describe the methods of handling data and combining results of studies, if done, including measures of consistency (e.g., $I^2$) for each meta-analysis. | 9 |



| Section/topic | # | Checklist item | Reported on page # |
|---|---|---|---|
| Risk of bias across studies | 15 | Specify any assessment of risk of bias that may affect the cumulative evidence (e.g., publication bias, selective reporting within studies). | 9 |
| Additional analyses | 16 | Describe methods of additional analyses (e.g., sensitivity or subgroup analyses, meta-regression), if done, indicating which were pre-specified. | 9 |
| **RESULTS** | | | |
| Study selection | 17 | Give numbers of studies screened, assessed for eligibility, and included in the review, with reasons for exclusions at each stage, ideally with a flow diagram. | 9 |
| Study characteristics | 18 | For each study, present characteristics for which data were extracted (e.g., study size, PICOS, follow-up period) and provide the citations. | Table 1, appendix 2 |
| Risk of bias within studies | 19 | Present data on risk of bias of each study and, if available, any outcome level assessment (see item 12). | 14, appendix 3 |
| Results of individual studies | 20 | For all outcomes considered (benefits or harms), present, for each study: (a) simple summary data for each intervention group (b) effect estimates and confidence intervals, ideally with a forest plot. | 14-19 |
| Synthesis of results | 21 | Present results of each meta-analysis done, including confidence intervals and measures of consistency. | 14-19 |
| Risk of bias across studies | 22 | Present results of any assessment of risk of bias across studies (see Item 15). | 14-19 |
| Additional analysis | 23 | Give results of additional analyses, if done (e.g., sensitivity or subgroup analyses, meta-regression [see Item 16]). | NA |

For peer review only

| **DISCUSSION** | | | |
|---|---|---|---|
| Summary of evidence | 24 | Summarize the main findings including the strength of evidence for each main outcome; consider their relevance to key groups (e.g., healthcare providers, users, and policy makers). | 19-21 |
| Limitations | 25 | Discuss limitations at study and outcome level (e.g., risk of bias), and at review-level (e.g., incomplete retrieval of identified research, reporting bias). | 21 |
| Conclusions | 26 | Provide a general interpretation of the results in the context of other evidence, and implications for future research. | 22 |
| **FUNDING** | | | |
| Funding | 27 | Describe sources of funding for the systematic review and other support (e.g., supply of data); role of funders for the systematic review. | 22 |

*From:* Moher D, Liberati A, Tetzlaff J, Altman DG, The PRISMA Group (2009). Preferred Reporting Items for Systematic Reviews and Meta-Analyses: The PRISMA Statement. PLoS Med 6(7): e1000097. doi:10.1371/journal.pmed1000097

For more information, visit: **www.prisma-statement.org**.

Page 2 of 2

For peer review only

BMJ Open

# A Systematic Review of Endovascular Stent Grafting Versus Open Surgical Repair for the Elective Treatment of Arch/Descending Thoracic Aortic Aneurysms



| | |
|---|---|
| Journal: | *BMJ Open* |
| Manuscript ID | bmjopen-2020-043323.R1 |
| Article Type: | Original research |
| Date Submitted by the Author: | 06-Jan-2021 |



| | |
|---|---|
| Complete List of Authors: | McCarthy, Andrew; Northumbria University, Faculty of Health and Life Sciences<br>Gray, Joanne; Northumbria University, Health and Life Sciences<br>Sastry, Priya; John Radcliffe Hospital, Cardiothoracic Surgery<br>Sharples, Linda; London School of Hygiene and Tropical Medicine, Medical Statistics<br>Vale, Luke; Newcastle University, Health Economics Group, Population Health Sciences Institute<br>Cook, Andrew; University of Southampton, Wessex Institute; University of Southampton, Southampton Clinical Trials Unit<br>Mcmeekin, Peter; Northumbria University, Faculty of Health and Life Sciences<br>Freeman, Carol; Papworth Hospital NHS Foundation Trust, Papworth Trials Unit Collaboration<br>Catarino, Pedro; Papworth Hospital NHS Foundation Trust<br>Large, Stephen; Papworth Hospital NHS Foundation Trust, Cardiac Surgery |



| | |
|---|---|
| <b>Primary Subject Heading</b>: | Surgery |
| Secondary Subject Heading: | Surgery |
| Keywords: | Cardiothoracic surgery < SURGERY, Vascular surgery < SURGERY, Thoracic surgery < SURGERY |
| | |


SCHOLARONE™
Manuscripts

*I, the Submitting Author has the right to grant and does grant on behalf of all authors of the Work (as defined in the below author licence), an exclusive licence and/or a non-exclusive licence for contributions from authors who are: i) UK Crown employees; ii) where BMJ has agreed a CC-BY licence shall apply, and/or iii) in accordance with the terms applicable for US Federal Government officers or employees acting as part of their official duties; on a worldwide, perpetual, irrevocable, royalty-free basis to BMJ Publishing Group Ltd ("BMJ") its licensees and where the relevant Journal is co-owned by BMJ to the co-owners of the Journal, to publish the Work in this journal and any other BMJ products and to exploit all rights, as set out in our licence.*

*The Submitting Author accepts and understands that any supply made under these terms is made by BMJ to the Submitting Author unless you are acting as an employee on behalf of your employer or a postgraduate student of an affiliated institution which is paying any applicable article publishing charge ("APC") for Open Access articles. Where the Submitting Author wishes to make the Work available on an Open Access basis (and intends to pay the relevant APC), the terms of reuse of such Open Access shall be governed by a Creative Commons licence – details of these licences and which Creative Commons licence will apply to this Work are set out in our licence referred to above.*

*Other than as permitted in any relevant BMJ Author's Self Archiving Policies, I confirm this Work has not been accepted for publication elsewhere, is not being considered for publication elsewhere and does not duplicate material already published. I confirm all authors consent to publication of this Work and authorise the granting of this licence.*

For peer review only

# A Systematic Review of Endovascular Stent Grafting Versus Open Surgical Repair for the Elective Treatment of Arch/Descending Thoracic Aortic Aneurysms

Andrew McCarthy [1], Joanne Gray [1], Priya Sastry [2], Linda Sharples [3], Luke Vale [4], Andrew Cook [5,6,7]; Peter McMeekin [1], Carol Freeman [8], Pedro Catarino [9], Stephen Large [9]

[1] Faculty of Health and Life Sciences, Northumbria University, Newcastle Upon Tyne, UK

[2] John Radcliffe Hospital, Oxford University Hospitals, UK

[3] Department of Medical Statistics, London School of Hygiene and Tropical Medicine, London, UK

[4] Health Economics Group, Population Health Sciences Institute, Newcastle University, Newcastle Upon Tyne, UK

[5] Wessex Institute, University of Southampton, UK

[6] Southampton Clinical Trials Unit, University of Southampton, UK

[7] University Hospital Southampton NHS Foundation Trust, UK

[8] Papworth Trials Unit Collaboration, Royal Papworth Hospital NHS Foundation Trust, Cambridge

[9] Royal Papworth Hospital NHS Foundation Trust, Cambridge

**Corresponding Author:**

**Andrew McCarthy-** Coach Lane Campus West, Northumbria University, Newcastle upon Tyne, United Kingdom, NE7 7XA

andrew2.mccarthy@northumbria.ac.uk

Tele: 0191 215 6718

For peer review only

## ABSTRACT

**Objective:** to review comparisons of the effectiveness of endovascular stent grafting (ESG) against open surgical repair (OSR) for treatment of chronic arch or descending thoracic aortic aneurysms (TAA).

**Design** Systematic review and meta-analysis

**Data sources** MEDLINE, EMBASE, CENTRAL, WHO International Clinical Trials Routine data collection, current controlled trials, clinical trials, and the NIHR portfolio were searched from January 1994 through March 2020.

**Eligibility criteria for selective studies** All identified studies that directly compared ESG and OSR, including RCTs, quasi-randomized and non-randomized controlled trials, comparative cohort studies and case-control studies matched on main outcomes were sought. Study participants had to receive elective treatments for elective arch and/or descending thoracic aortic aneurysms (TAA). Studies were excluded where other thoracic aortic conditions (e.g. rupture or dissection) were reported, unless results for patients receiving elective treatment for arch/descending TAA were reported separately.

**Data extraction and synthesis** Data was extracted by one reviewer and checked by another. Risk of Bias was assessed using the ROBINS-I tool. Meta-analysis was conducted using a random-effects model. Where meta-analysis not appropriate, results were reported narratively.

**Results** Five comparative cohort studies met inclusion criteria, reporting a total of 3955 ESG and 21,197 OSR patients. Meta-analysis of unadjusted short-term (30 day) all-cause mortality favoured ESG (OR ratio: 0.75; 95%CI (0.55-0.1.03)). Heterogeneity was identified between larger and smaller studies. Meta-analysis of adjusted short-term all-cause mortality favoured ESG OR 0.71, 95%CI (0.51, 0.98), no heterogeneity. Longer-term (beyond 30 days) survival from all-cause mortality favoured OSR in larger studies and ESG in smaller studies. Freedom from re-intervention in the longer-term




For peer review only - http://bmjopen.bmj.com/site/about/guidelines.xhtml

favoured OSR. Studies reporting short-term non-fatal complications suggest fewer events following ESG.

**Conclusions** There is limited and increasingly dated evidence on the comparison of ESG and OSR for treatment of arch/descending TAA

**POSPERO registration number** CRD42017054565

**KEY WORDS**

Systematic Review, Meta-analysis, Thoracic Aortic Aneurysm, Open Surgical Repair, Endovascular Stent Graft

**STRENGTHS AND LIMITATIONS OF THIS STUDY**

Strength:

- This is the first systematic review to investigate two-armed studies looking only at elective ESG versus OSR for treatment of chronic arch or descending thoracic aortic aneurysms. Other reviews include dissections, ruptures and treatment for other conditions.
- This systematic review inclusion criteria were broad and sought to include evidence from both randomized and non-randomized study designs.

Limitation:

- Statistical analysis of non-fatal complications was not possible due to the limited and inconsistent reporting of such complications.

For peer review only



For peer review only - http://bmjopen.bmj.com/site/about/guidelines.xhtml

## INTRODUCTION

### Rationale

A thoracic aortic aneurysm (TAA) is an abnormal expansion of the aorta at any part of its course from the heart to the diaphragm. Aneurysms manifest with acute symptoms or they may be chronic. If untreated, TAA can continue to expand and the annual risk of aneurysm rupture increases from 2% in aneurysms smaller than 5cm in diameter to 7% in aneurysms over 6cm in diameter[1]. The majority of aneurysms (and therefore studies) centre on the ascending or abdominal segments of the aorta, arch and descending aneurysms are relatively under-studied. Therefore, this review focused on chronic aneurysms affecting the arch or descending thoracic aorta.

There are currently two main methods of repair for arch/descending TAA: Open Surgical Repair (OSR) and Endovascular Stent Grafting (ESG). OSR involves replacing the aneurysmal segment of aorta with a surgical graft during open surgery using heart-lung bypass. As the arch and descending aorta supply blood to the brain and spinal cord, OSR is high risk, with reported mortality and paraplegia rates of approximately 5% and 10% [2] respectively. In ESG a stent is placed across the aneurysm, providing a new conduit through which the blood flows. ESG is most often selected for patients who are considered at high risk of death or permanent disability from OSR. However, some patients may not have a suitable anatomy/morphology for ESG procedures [2]. Concerns have also been raised about long-term durability and safety of the implanted devices [3 4] due to complications such as stent migration or endoleaks, with the incidence of late endoleaks (leaks around or through the stent graft that occur more than one year after insertion) reported as being between 2.5% and 27.8% [5]. The need for re-intervention due to technical failures of the stent following ESG is also high (23% at 5 years) [6 7]. With each re-intervention there is an added risk of complication either due to the increased complexity of the procedure or deteriorating health of the patient [2].

One previous systematic review comparing only elective OSR and ESG for descending and arch TAAs has been reported [8]. This systematic review [8] was restricted to data from randomized controlled



For peer review only - http://bmjopen.bmj.com/site/about/guidelines.xhtml

trials in which patients were randomly assigned to ESG and OSR, but no eligible trials were identified when searching relevant data based up to Jan 2016. There are other systematic reviews [9 10] that have included non-randomized studies; however, these reviews also included emergency cases and other thoracic aortic pathologies (e.g. dissection or rupture). As such, they may not represent a true comparison of elective ESG and OSR procedures for descending thoracic aortic aneurysms. Given the lack of RCT evidence, this systematic review includes non-randomized comparative studies of the relative effectiveness of OSR or ESG.

The decision to operate requires a favourable balance between the risks and benefits of that intervention compared to those attached to the expected outcomes of the disease. This balance is in turn influenced by a number of factors. Aneurysm size correlates to the risk of death (if left untreated) and so patients are usually referred once the aneurysm crosses a chosen threshold. At that point, patient fitness (*and life expectancy*), the reality or possibility of an intrinsic weakness affecting the whole aorta, the technical feasibility of the proposed intervention and patient wishes, come into play. This systematic review takes a step back from these issues and examines only the outcomes for patients in whom a decision to intervene has been made. This review is an essential starting point for the analysis of effective treatments for thoracic aortic aneurysms.

**Objectives**

The aim of this systematic review was to assess the effectiveness of endovascular stent grafting (ESG) compared to open surgical repair (OSR) as a treatment for chronic arch or descending TAA.



For peer review only - http://bmjopen.bmj.com/site/about/guidelines.xhtml

## METHODS

### Protocol and registration

The review protocol was registered in the PROSPERO database (CRD42017054565)[11].

### PUBLIC PATIENT INVOLVEMENT

There was no patient and public involvement in the whole process of conducting this review.

### Eligibility criteria

### Study designs

All identified studies that directly compared ESG and OSR, including RCTs, quasi-randomized and non-randomized controlled trials, comparative cohort studies and case-control studies matched on main outcomes were sought [12].

Non-randomized studies that controlled for selection bias were included – irrespective of study size. Acceptable methods for addressing selection bias included matching patients at baseline for important covariates, matching patients using propensity scores or adjustment for important covariates during analysis. Non-randomized studies that did not use these methods but included larger sample sizes (more than 250 patients) were also included as they have the potential to reduce sampling variation relative to smaller unmatched studies[13].

Non-English language papers were noted and, where possible, data were extracted from the abstract. No translation of full text articles was possible.

### Study Participants

Study participants had to receive elective treatments for arch and/ or descending thoracic aortic aneurysms. Where emergency interventions or other thoracic aortic conditions (such as intramural haematoma, rupture, and dissection) were reported, studies were excluded, unless results for patients receiving elective treatment for arch/descending TAA were reported separately. Studies



For peer review only - http://bmjopen.bmj.com/site/about/guidelines.xhtml

that reported secondary (re-intervention) procedures for arch/descending TAAs were included only if the original, primary procedure for each participant could be identified.

**Study interventions**

Open Surgical Repair was defined as replacement of the aneurysmal aorta with a prosthetic conduit via a sternotomy or thoracotomy with circulatory support. Endovascular stent grafting was defined as transluminal introduction of a stent-graft under X-ray guidance.

**Outcome measures**

This review assessed short-term and long-term clinical effectiveness. Short-term was defined as either within 30-days of the intervention and/or time until discharge following the intervention. Since most studies did not report long-term outcomes conditional on surviving the short-term period, long-term analyses covered the time from intervention until the end of follow-up. Primary outcomes were all-cause mortality and aneurysm-related mortality. Secondary outcomes were re-interventions and non-fatal complications. The latter were sub-classified as vascular, neurological, cardiac, respiratory and 'other' complications.

**Information sources**

MEDLINE, EMBASE, CENTRAL, WHO International Clinical Trials Routine data collection, Current Controlled Trials, Clinical Trials, and the UK National Institute for Health Research portfolio were searched. The search strategy (Appendix 1) was adapted as appropriate for each database (for example to allow for variations in controlled vocabulary terms and syntax [14]). Electronic searches identified publications between 1994, date of first reported case of ESG for descending and arch TAA[15], and March 2020.

Websites of regulatory bodies and Health Technology Assessment agencies were screened for unpublished relevant reports. A citation search of all included studies was conducted via Web of



For peer review only - http://bmjopen.bmj.com/site/about/guidelines.xhtml

Science. Studies from systematic reviews that satisfied the inclusion criteria of this review were screened for eligibility.

**Study selection**

All titles and abstracts were screened by one reviewer (AM). A second investigator (JG) reviewed 10% of all titles and abstracts classed as eligible or not eligible and all titles and abstracts classified as unclear by the first reviewer. The full text of selected studies was assessed by one investigator (AM) and classified as relevant, not relevant or unclear according to the inclusion criteria. A second investigator (JG) reviewed 10% of all papers, as well as all studies deemed unclear. Disagreements at either stage were resolved by discussion and where necessary a third reviewer (PM) arbitrated. Where required, a clinical expert (PS) was asked to screen papers.

**Data collection process**

A data extraction form was developed based on The Cochrane Collaboration data collection form [16]. Data were extracted by one reviewer (AM) and checked by a second reviewer (JG) for accuracy. Disagreements were resolved by discussion or arbitration by a third reviewer (PM).

**Data items**

Reported short-term and longer-term all-cause mortality, as well as short-term and longer-term aneurysm-related mortality were collected. All reported complications were recorded and grouped according to expert clinical guidance as described above as neurological, cardiac, respiratory or 'other' complications, with endoleaks following ESG recorded separately.

**Risk of bias in individual studies**

Risk of bias tools used were specific to the study design. As described later only non-randomized study designs were identified. Therefore, the ROBINS-I tool [17] was used. The ROBINS-I tool classifies the risk of bias in seven domains (*confounding; selection of participants; classification of*



For peer review only - http://bmjopen.bmj.com/site/about/guidelines.xhtml

*interventions; deviations from intended interventions; missing data; measurement of outcomes; selection of the reported results*) and overall as either critical, severe, moderate, low, or unclear.

**Synthesis of results**

Point estimates and precision of the incidence of short and long-term events and unadjusted and adjusted comparisons (odds ratios (OR) hazard ratios (HR)) were recorded. Meta-analysis of short-term event-comparisons was based on the OR with 95% confidence interval (CI) and conducted using RevMan [18]. An overall treatment effect was calculated for the log (OR) of each outcome using a (normal) random effects model. Where comparisons could not be made, data were tabulated and reported narratively.

Heterogeneity between studies was assessed according to Cochrane guidelines, on clinical criteria, by visual inspection of plots of the data, from the chi-square test for heterogeneity, and the I-squared statistic. An $I^2$ statistic of 25-50% indicated low heterogeneity; 50-75% indicated moderate heterogeneity, and over 75% indicating significant heterogeneity.

**RESULTS**

**Study selection**

The search identified 5163 unique studies. Of these, 5078 studies were not eligible. Ineligibility was primarily because of irrelevant anatomy (ascending, abdominal, or peripheral aneurysms), irrelevant pathology (acute dissection or other acute aortic syndromes), or because the study pooled data from a mixture of anatomical and pathological situations such that data on chronic arch and DTA aneurysms could not be extracted with confidence. Thus 85 papers underwent full text screening. 75 papers were rejected at this stage leaving 10 papers to be included in the full review. Six papers reported data from the Gore TAG Trial [19-24], leaving five unique comparative studies that fulfilled the inclusion criteria. Further details of the selection process are shown in the PRISMA diagram (Figure 1).



For peer review only - http://bmjopen.bmj.com/site/about/guidelines.xhtml

One study, Goodney et al 2011, reported both an unadjusted analysis for the unadjusted cohort and an analysis of a subgroup of propensity score matched patients. This review reports information from both cohorts separately but does not include both in any analysis to avoid double-counting. Brief summaries of the identified studies are given in Table 1, with detail provided in Appendix 2. Overall, the five studies included data on 3955 ESG and 21,197 OSR patients and all studies were conducted in Europe or the USA. Two studies had non-overlapping or only partially overlapping recruitment periods between the ESG and OSR intervention groups (Gore Tag and Piffaretti et al 2007[19 25]). There were clear differences in patient characteristics between the two treatment groups. ESG patients were consistently older, with average age differences ranging from 2 to 9 years in all but one analysis (Goodney et al 2011 the propensity matched cohort). Similarly, in most studies the proportion of men was greater in the ESGgroup.



For peer review only - http://bmjopen.bmj.com/site/about/guidelines.xhtml

*Table 1: Study characteristics*

| Study | **Von Allmen et al 2014** | **Goodney et al 2011 (2 analyses*)** | **Gore TAG** | **Hughes et al 2014** | **Piffaretti et al 2007** |
|---|---|---|---|---|---|
| **METHODS** | | | | | |
| **Study Design** | Comparative cohort | Comparative cohort | Comparative cohort | Comparative cohort | Comparative cohort |
| Method of matching | None | 1. None 2. propensity matched | ESG patients judged eligible for OSR | None | OSR patients judged eligible for ESG |
| **PARTICIPANTS** | | | | | |
| **Country** | UK | USA | USA | USA | Italy |
| **Setting** | Multi-centre – Hospital episode statistics | Multi-centre – Medicare data | Multi-centre | Multi-centre | Single-centre |
| **Time frame** | 2006 to 2011 | 1. 1998-2007 2. 2003-2007 | ESG 1999-2001, OSR pre 1999 (n=44) & 1999-2001 (n=50) | 1998-2007 | ESG 2000-2007, OSR 1996-2000 |
| **Recruitment** | Total 618 ESG 354 OSR 264 OSR | 1. Total 13,998 (ESG 2433, OSR 11,565) | Total 234 ESG 140 OSR 94 | Total 8967 ESG 712 OSR 8255 | Total 28 ESG 17 OSR 11 |



For peer review only - http://bmjopen.bmj.com/site/about/guidelines.xhtml

| | | | | | |
|---|---|---|---|---|---|
| | | 2. Total 1100 (ESG 550, OSR 550) | | | |
| **Age summary** | median (IQR) ESG 73(66,78), OSR 71 (63-76) | mean (95% C.I.) 1. ESG 75.9 (75.6, 76.1), OSR 73.8 (73.7, 73.9) 2. ESG 71.1 (70.8, 71.4), OSR 70.7 (70.7, 71.1) | mean(SD) ESG 75.9 (10.4), OSR 68.2 (10.2) | median (IQR) ESG 72 (63-78), OSR 63 (52, 72) | mean (SD) ESG 66 (10), OSR 61 (13) |
| **Proportion male** | ESG 232 (65.5%), OSR 137 (51.9%) | % (95% C.I.): 1. ESG 58.7 (56.7, 60.7), OSR 55.4 (54.8, 56.3) 2. ESG 64.0 (59.9, 68.0), OSR 68.1% (64.2, 72.0) | ESG 90(57%), OSR 48 (51%) | 5415 (61.3%) ESG, 437 (65.6%) OSR | ESG 14 (82%), OSR 8 (73%) |
| **OUTCOMES** | | | | | |
| **Short-term** | Death from any cause | Death from any cause | Death from any cause | Death from any cause | Death from any cause |



For peer review only - http://bmjopen.bmj.com/site/about/guidelines.xhtml

| | | | | | |
|---|---|---|---|---|---|
| | Re-intervention All-cause mortality (unadjusted | | Complications | Complications | Complications |
| **Long-term** | Time to death from any cause Time to aortic-related death Time to aortic-related re-intervention | Time to death from any cause | Time to death from any cause Time to aneurysm-related death aneurysm-related mortality Time to re-intervention Time to major adverse event | Not reported | Time to death from any cause |
| * 1. Unmatched cohort, 2. Propensity matched cohort | | | | | |

For peer review only

**Risk of bias within studies**

The propensity-matched analysis of Goodney et al (2011) [26] was judged to be of overall moderate risk of bias and four studies were at severe risk of bias due to the potential for confounding (Appendix 3).



For peer review only - http://bmjopen.bmj.com/site/about/guidelines.xhtml

Two large cohorts derived from UK and USA administrative databases (Von Allmen et al 2014 and Hughes et al 2014) [27 28] did not attempt to address selection bias at the design stage, but did adjust for some confounders in the analysis. A third study (Goodney et al 2011), using USA administrative data, included both unadjusted population and analysis of a subset of the data defined by propensity score matching. Propensity score methods rely on all important confounders being available and included[29]. As this study did not include all confounders considered important, such as aneurysm size, this study was judged to have moderate risk of bias due to confounding.

One study (Gore TAG)[19] included only ESG patients who would also have been eligible for OSR and the another study (Piffaretti et al 2007) [25] included only OSR patients who would be suitable for ESG, both according to clinician judgement. Since suitability was decided retrospectively, such matching may not be robust.

A further potential bias with each study was selective reporting. Although the measurements and analyses of the outcomes were clearly defined within each study, no evidence of a pre-study analysis plan was identified.

**Synthesis of results**

**Short-term death from any cause (30-day or in hospital)**

*Unadjusted Mortality:*

All five studies [19 25-28] reported unadjusted 30 day or in-hospital mortality (Figure 2). Overall, the odds of short-term mortality were lower in the ESG group than the OSR group (OR: 0.75; 95 %CI. (0.55-1.03)). Moderate heterogeneity was present in the overall analysis ($I^2$= 40%), mainly due to differences between the two smaller studies [19 25] and the three larger studies [26-28], with a large $I^2$ for subgroup differences between the larger studies subgroup and the smaller studies subgroup of 84.5%.



For peer review only - http://bmjopen.bmj.com/site/about/guidelines.xhtml

For the three larger studies[26-28] (3798 ESG and 21,092 OSR patients), the treatment effect for death was smaller (OR: 0.84 95%CI (0.72, 0.98)) than that for the two smaller studies [19 25](157 ESG and 105 OSR patients, combined OR: 0.18 95%CI (0.06, 0.59)). There was no evidence of heterogeneity in results between these subgroups ($I^2$=0).

*Sensitivity analysis*

Hughes et al 2014 was the only study to include aortic arch aneurysms. Arch aneurysms tend to be more complex procedures and are mostly conducted using OSR. A sensitivity analysis excluding this study was therefore conducted (Figure 3).

Overall, the odds of short-term mortality were lower in the ESG group than the OSR group (OR: 0.73; 95 %CI. (0.45-1.18)). Moderate heterogeneity was present in the overall analysis ($I^2$= 42%), mainly due to differences between the two smaller studies and the two larger studies, with a large $I^2$ for subgroup differences between the larger studies subgroup and the smaller studies subgroup of 65.1%.

For the two larger studies (3086 ESG and 12837 OSR patients), the treatment effect for death was smaller (OR: 0.85 95%CI (0.72, 1.00)) than that for the two smaller studies (157 ESG and 105 OSR patients, combined OR: 0.20 95%CI (0.04, 1.06)). There was no evidence of heterogeneity in results within the large subgroup ($I^2$= 0) and evidence of low heterogeneity withing the small studies subgroup ($I^2$=26%).

*Adjusted Mortality:*

The three larger studies [26-28] included adjusted analysis of short-term mortality (Figure 4). Goodney et al 2011 used stratified propensity matching. Hughes et al 2014 [27] adjusted for preoperative comorbidities, independent predictors of mortality and post-operative complications and the Von Allmen 2014 study adjusted for age and sex. The OR for ESG, relative to OSR, for these three studies



For peer review only - http://bmjopen.bmj.com/site/about/guidelines.xhtml

combined was 0.71, 95%CI (0.51, 0.98). No evidence of heterogeneity was identified in this meta-analysis ($I^2$=0%)

**All-cause mortality (following hospital discharge or after 30 days)**

Meta-analysis of the longer-term all-cause mortality was not possible as follow-up varied between the four studies that reported this outcome (Gore Tag, Goodney et al 2011, Piffaretti et al 2007 and Von Allmen 2014) [19 25 26 28] . The results in the larger studies (Goodney et al 2011, Von Allmen 2014)[26 28] differed from those in the smaller studies (Gore Tag and Piffaretti et al 2007)[25 30]. We note that the analyses included the short-term deaths as well as those occurring beyond hospital discharge or after 30 days.

Von Allmen et al 2014 reported time to death from any cause up to five years, throughout which OSR had higher overall survival. Adjusting for age and sex, ESG had higher odds of death at one year (OR: 1.10 95%CI (0.70, 1.73); p=0.667). Additionally, the hazard ratio for ESG relative to OSR up to five years, adjusting for age and sex, was HR=1.45 (95%CI [1.08, 1.94], p=0.013).

Goodney et al 2011 also reported time to death from any cause up to five years. Adjusting for age, sex, race, era of procedure and Charlson comorbidity score, five-year survival for OSR in the unmatched cohort was higher than for ESG (89% versus 79%, log-rank p<0.0001). In the propensity-matched cohort, five-year life-table analysis reported higher survival in the OSR group (81% 95%CI (77%-85%)) than for the ESG group (73% 95%CI (68%, 76%)) (log-rank p=0.007).

In the Gore TAG study, Time to death from any cause over five years did not differ between the ESG and OSR groups, with survival probability at five years in both groups of 63% (log rank P value= 0.625). Piffaretti et al 2007 reported survival overall in the ESG group up to five years and the OSR group up to 13 years. For the five years in which survival is reported in both groups, ESG had a higher



For peer review only - http://bmjopen.bmj.com/site/about/guidelines.xhtml

survival compared to OSR although this was based on only 4 deaths, and the follow-up periods differed substantially (log rank p>0.05).

**Aneurysm/Aortic-related mortality**

Meta-analysis of Aneurysm/aortic-related mortality could not be conducted. No studies reported aneurysm-related mortality during hospitalization or up to 30 days separately to the long-term. Von Allmen et al 2014 defined  aortic-related mortality as any aortic-related death occurring within five years of the index operation according to ICD-10 codes referring to aorta, or cause of deaths closely related to complications of surgery (e.g. paraplegia). Time to aortic-related death did not differ between OSR group than the ESG group during the five-year follow-up (log-rank p=0.270). This study[28] also reported adjusted (for age and sex) five year HR of 1.27 (0.81 to 1.98; P =0.298) for ESG patients relative to OSR patients.

Gore TAG[19] defined aneurysm related mortality as deaths that occurred in hospital or within 30 days of initial procedure or any reintervention or a death due to the aneurysm or the treatment device and reported up to five years, with longer time to aneurysm-related death in the ESG group over this period (log-rank p= 0.012).

**Short-term re-interventions**

One study reported aortic-related re-interventions in 21 (5.9%) ESG patients and 4 (1.5%) OSR patients, an adjusted OR of 2.97 (95%CI [1.09, 8.10], p=0.033) for ESG versus OSR (Von Allmen et al 2014[28]).

**Long-term re-interventions**

Two studies reported this outcome (Von Allmen et al 2014[28] and Gore TAG [19]). Von Allmen et al 2014[28] reported time to aortic-related re-interventions up to five years. Time to re-intervention was



For peer review only - http://bmjopen.bmj.com/site/about/guidelines.xhtml

longer in the OSR group compared to the ESG group (log-rank p=0.007), with adjusted (for age and sex) hazard ratio at five years of 1·70 (95%CI [1·11 to 2·60], p =0·014). In Gore TAG[19], time to re-intervention over five years was longer in the ESG group (log-rank P=0.011). We note that these analyses included short-term re-interventions.

**Complications**

Table 2 summarises the short-term complications reported in all five studies[19 25-28]. Most studies reported a small number of neurological, pulmonary, cardiac and other complications. However, one larger study (Hughes et al 2014)[27] reported lower odds for ESG patients for neurological complications, OR of 0.48 (95%CI (0.26, 0.86), p=0.015), pulmonary complications, OR: 0.38 (95%CI (0.21, 0.67), p=0.001) and cardiac complications, OR: 0.24 (95%CI (0.15, 0.37), p<0.001). The Gore TAG study[19] reported endoleak rates for ESG patients of 8.5%.

*Table 2: Short-term complications*

| Study | Gore TAG | | Piffaretti et al 2007 | | Hughes et al 2011 | |
|---|---|---|---|---|---|---|
| **Arm (participants)** | **ESG (n=140)** | **OSR (n=94)** | **ESG (n=17)** | **OSR (n=11)** | **ESG (n=712)** | **OSR (n=8255)** |
| **NEUROLOGICAL** | | | | | | |
| **Paraplegia** | NR | NR | NR | NR | NR | NR |
| **Paraplegia/ Paraperisis** | 4 (3%) | 13 (14%) | NR | NR | NR | NR |
| **Cerebral Vascular Accident** | 5 (4%) | 5 (4%) | 2 (9%) | 1 (12%) | NR | NR |
| **Neurological-unspecified type** | NR | NR | NR | NR | 20 (2.8%) | 273 (3.3%) |
| **RESPIRATORY** | | | | | | |

For peer review only



For peer review only - http://bmjopen.bmj.com/site/about/guidelines.xhtml

| | | | | | | |
|---|---|---|---|---|---|---|
| **Respiratory failure** | 5 (4%) | 19 (20%) | NR | NR | NR | NR |
| **Pneumonia** | NR | NR | 2 (12%) | 3 (27%) | NR | NR |
| **Pulmonary-unspecified type** | NR | NR | NR | NR | 17 (2.4%) | 462 (5.6%) |
| **CARDIAC** | | | | | | |
| **Myocardial Infarction** | 0 (0%) | 1(1%) | 0 (0%) | 1 (9%) | NR | NR |
| **Cardiac-unspecified type** | NR | NR | NR | NR | 28 (2.9%) | 1252 (15.2%) |
| **ENDOLEAKS** | | | | | | |
| **Endoleaks** | 12 (8.5%) | NR | NR | NR | NR | NR |
| **OTHER** | | | | | | |
| **Peripheral vascular disease** | 20 (14%) | 4 (4%) | NR | NR | NR | NR |
| **Renal failure** | 2 (1%) | 12 (13%) | NR | NR | NR | NR |
| **Wound infection/dehiscence** | 5 (4%) | 10 (11%) | NR | NR | NR | NR |
| **GI complication** | 3 (2%) | 6 (6%) | NR | NR | NR | NR |
| **Post-implant syndrome** | NR | NR | 4(18%) | 0(0%) | NR | NR |
| *Note: Goodney et al 2011 and Von Allmen et al 2014 did not report short-term complications* | | | | | | |

**DISCUSSION**

This is the first systematic review that reports results from non-randomized comparative studies regarding the effectiveness of elective ESG and OSR for arch and or descending TAA. Previous reviews have either included single arm studies only[31] or were comparative reviews that included non-elective procedures[9 32], or mixed pathologies such as dissections[10 33]. The results of these



For peer review only - http://bmjopen.bmj.com/site/about/guidelines.xhtml

reviews may not be applicable to patients undergoing elective procedures for descending or arch TAA pathology.

In this review only five comparative cohort studies were identified. The lack of RCTs comparing ESG and OSR for elective arch/descending thoracic aortic aneurysms may reflect the lack of equipoise from practitioners and patients. Well-designed non-randomized trials can demonstrate a high degree of concordance with the results of well-designed RCTs, provided that all confounders are adequately accommodated in the design and/or analysis[34]. However, the studies identified in this review had moderate to serious risk of bias, mainly due to potential for confounding.

Although, complications in the short-term also appear to favour ESG, evidence regarding short-term complications is either lacking or inconsistently reported. All five studies[19 25-28] suggested that the less invasive nature of ESG confers lower short-term mortality and fewer short-term non-fatal complications than OSR. The two small unadjusted studies[19 25] had a much larger effect than larger studies[26-28]. These larger studies used more robust methods for addressing confounding; this may indicate the presence of publication bias where only those smaller studies with larger effect sizes have been published.

Longer-term results are difficult to interpret since most data were not presented in a way to separate early risk from longer term survival. The two smaller studies[19 25] reported continuing survival benefit for ESG patients in the long-term but are limited by small numbers, different recruitment periods, and failure to exclude early risk. The survival advantage associated with ESG in the Gore TAG study disappears by year five, which suggests that the hazards are lower for OSR patients that survive the initial procedure. Conversely, the two larger studies (Von Allmen et al 2014 and Goodney et al 2011[26 28]) report that the short-term survival advantage of ESG were reversed by twelve months, and that the OSR group hadan increasing survival advantage thereafter. Similarly, time to re-intervention favoured ESG in the smaller Gore TAG study, whilst time to aortic-related re-interventions favoured OSR in the larger study Von Allmen et al 2014[28].



For peer review only - http://bmjopen.bmj.com/site/about/guidelines.xhtml

The evidence regarding mortality is inconsistent across the studies with more recent, larger studies[26-28] reporting a more modest survival benefit for ESG in the short-term and a survival benefit for OSR in the long-term. The two[19 25], smaller studies suggest a large reduction in the risk of mortality and short term complications in the short run and a survival benefit in the long-run for ESG.

Inconsistency of results across studies may be due to heterogeneity of the study populations, both within and across studies. Differences in recruitment periods may reflect changes in technology and practice in both groups. The larger studies [26-28] were based on patients recruited up to twenty years later than the smaller studies. Outcomes may have improved differentially with OSR patients receiving proportionally better outcomes due to general improvements in surgical practice and patient selection over time[35]. A potential reason for the reported long-term survival benefit of OSR in more recent, larger studies may be differences in patient risk in each intervention group. Open surgical repair is advocated for younger, lower-risk patients, and EVAR is preferred for older, higher-risk patients[36]. This selection bias may not have been adequately accommodated in adjusted analyses.

The three large studies[26-28] were based on administrative health records with case ascertainment that depends on accurate coding, which can vary across and within providers[37]. Furthermore, one of the small studies, Piffaretti et al 2007[25], recruited patients from one centre which may have compromised external validity as they may for example, depend on the expertise within a centre and the nature of the patients that centre treats[30 38]. Additionally, the Gore TAG study[19] utilised a more restricted inclusion criteria for ESG patients compared to those who received OSR, potentially introducing heterogeneity between the two groups.

The headline conclusion of this review is that whilst there is a degree of variability, OSR is better for long-term survival and avoidance of re-intervention, and that ESG is better for early outcomes. This conclusion is based on non-randomized comparisons in which the influence of important



For peer review only - http://bmjopen.bmj.com/site/about/guidelines.xhtml

confounders for choice of intervention (ESG or OSR) has not been adjusted for. Deeper exploration is required, but randomized data may be difficult to acquire as there are well-established guidelines about which patients are favoured for OSR and which for ESG. Thus, to compare ESG and OSR it is first necessary to establish an understanding of the reasons driving clinical management, for example by a structured consensus analysis such as a Delphi study. Thereafter, unless there is clinical equipoise about which patients are best served by OSR or ESG, the best quality evidence to establish the relative merits of ESG and OSR for arch/ DTA aneurysms is likely to be a systematic, prospective, observational, comparative study, using individual patient data.

**Limitations**

The findings in this review are limited by the number and potential for confounding of the five studies[19 25-28] identified, so that definitive conclusions are not possible. The adjustment for confounders is likely to be only partial and some bias in the estimates of differences in mortality and complications may persist, despite attempts to reduce bias in the larger studies identified. Statistical analysis of non-fatal complications was not possible due to limited and inconsistent reporting.

**CONCLUSIONS**

This systematic review is the first to consider evidence from non-randomized studies that directly compare ESG and OSR for the treatment of elective arch/descending thoracic aortic aneurysms. Other reviews conducted in this area have been complicated by the inclusion of treatments for emergency cases or other thoracic aortic pathologies (e.g. thoracic aortic ulcer, or dissection).

There is limited and increasingly dated evidence addressing the comparison of ESG and OSR in the management of elective arch and or descending thoracic aortic aneurysms. Further, high quality, evidence is required. Although large RCTs may not be possible in this relatively rare condition, future studies should use strong methodological study design in order to control for potential confounders



For peer review only - http://bmjopen.bmj.com/site/about/guidelines.xhtml

For peer review only

and reduce potential biases. Additionally, given the current conflicting evidence, it is important that comparisons of OSR and ESG are conducted in both the short-term and the long-term.

**FUNDING**

"This report is independent research funded by the National Institute for Health Research **(Health Technology Assessment, 11/147/03 - Effective Treatments for Thoracic Aortic Aneurysms (ETT AA study): A prospective cohort study).** The views expressed in this publication are those of the author(s) and not necessarily those of the NHS, the National Institute for Health Research or the Department of Health."

**CONFLICT OF INTEREST**

Andrew McCarthy, Joanne Gray, Linda Sharples, Priya. Sastry, Peter McMeekin, Andrew Cook have nothing to disclose. Luke Vale reports grants from NIHR Health Technology Assessment Programme, during the conduct of the study; and being a member of the NIHR Health Technology Assessment Programme Clinical Trials and Evaluation Panel from 2015-2018. Carol Freeman reports grants from DoH NIHR HTA Programme, during the conduct of the study. Pedro Catarino reports grants from NIHR, outside the submitted work. Stephen. Large reports grants from NIHR, during the conduct of the study.

**ACKNOWLEDGMENTS**

The authors would like to thank our Funders and the ETTAA Working Group for their support with this study.

**AUTHOR CONTRIBUTIONS**

Author contributions according to theorediT (contributor roles taxonomy). Conceptualization- AM, JG, PS, LS, LV, AC, PM, SL; Data curation- AM ;Formal Analysis- AM, JG, PM; Funding acquisition- JG, AC, LV, CF, PS, LS, SL; Investigation - AM, JG, PM, LV, PS; Methodology - AM, JG, PM, LV, AC, CF, PS,



For peer review only - http://bmjopen.bmj.com/site/about/guidelines.xhtml

LS, SL; Supervision - JG, PM, LV; Validation – AM, JG, PM, PS, LS, PC, SL; Writing – original draft - AM, JG, PM; Writing – review & editing - AM, JG, PM, AC, LV, CF, PS, LS, PC, SL.

**DATA SHARING AGREEMENT**

Data were extracted from the studies included in this Systematic Review and referenced below.

**REFERENCES**

1. Akin I, Kische S, Rehders TC, et al. Endovascular repair of thoracic aortic aneurysm. *Archives of medical science : AMS* 2010;6(5):646-52. doi: 10.5114/aoms.2010.17075 [published Online First: 2010/10/26]
2. Sastry P, Hughes V, Hayes P, et al. The ETTAA study protocol: a UK-wide observational study of 'Effective Treatments for Thoracic Aortic Aneurysm'. *BMJ Open* 2015;5(6) doi: 10.1136/bmjopen-2015-008147
3. Kim LG, Sweeting MJ, Epstein D, et al. Optimizing Surveillance and Re-intervention Strategy Following Elective Endovascular Repair of Abdominal Aortic Aneurysms. *Annals of Surgery* 2019;Publish Ahead of Print doi: 10.1097/sla.0000000000003625
4. Patel R, Sweeting MJ, Powell JT, et al. Endovascular versus open repair of abdominal aortic aneurysm in 15-years' follow-up of the UK endovascular aneurysm repair trial 1 (EVAR trial 1): a randomised controlled trial. *The Lancet* 2016;388(10058):2366-74. doi: 10.1016/S0140-6736(16)31135-7
5. Ricotta JJ. Endoleak management and postoperative surveillance following endovascular repair of thoracic aortic aneurysms. *Journal of Vascular Surgery* 2010;52(4, Supplement):91S-99S. doi: https://doi.org/10.1016/j.jvs.2010.06.149
6. Kalteis M, Haller F, Artmann A, et al. Experience and Outcomes After a Decade of Endovascular Abdominal Aortic Aneurysm Repair: A Retrospective Study From a Community-Based Single Center. *Annals of Vascular Surgery* 2012;26(3):330-37. doi: https://doi.org/10.1016/j.avsg.2011.06.012
7. Mertens J, Houthoofd S, Daenens K, et al. Long-term results after endovascular abdominal aortic aneurysm repair using the Cook Zenith endograft. *Journal of Vascular Surgery* 2011;54(1):48-57.e2. doi: https://doi.org/10.1016/j.jvs.2010.12.068
8. Abraha I, Romagnoli C, Montedori A, et al. Thoracic stent graft versus surgery for thoracic aneurysm. *Cochrane Database of Systematic Reviews* 2016(6) doi: 10.1002/14651858.CD006796.pub4
9. Health Quality O. Endovascular Repair of Descending Thoracic Aortic Aneurysm: An Evidence-Based Analysis. *Ontario Health Technology Assessment Series* 2005;5(18):1-59.
10. Walsh SR, Tang TY, Sadat U, et al. Endovascular stenting versus open surgery for thoracic aortic disease: Systematic review and meta-analysis of perioperative results. *Journal of Vascular Surgery* 2008;47(5):1094-98.e3. doi: https://doi.org/10.1016/j.jvs.2007.09.062
11. Gray J, McCarthy A, McMeekin P, et al. Effective Treatments for Thoracic Aortic Aneurysms (ETTAA study): protocol for a systematic review. *PROSPERO* 2016(CRD420170545565)
12. Reeves BC, Deeks JJ, Higgins JP, et al. Including Non-Randomized Studies. In: Higgins JP, Green S, eds. Cochrane Handbook for Systematic Reviews of Interventions2008.



For peer review only - http://bmjopen.bmj.com/site/about/guidelines.xhtml

13. Deeks J, Dinnes J, D'Amico R, et al. Evaluating non-randomised intervention studies. *Health Technology Assessment* 2003;7(27)
14. Texas Uo. Search Filters for Various Databases: PubMed 2016 [updated 3 October 2017. Available from: https://libguides.sph.uth.tmc.edu/search_filters accessed 14 July 2016.
15. Dake MD, Miller DC, Semba CP, et al. Transluminal Placement of Endovascular Stent-Grafts for the Treatment of Descending Thoracic Aortic Aneurysms. *New England Journal of Medicine* 1994;331(26):1729-34. doi: 10.1056/nejm199412293312601
16. Higgins JP, Green S. Guide to the Contents of a Cochrane Protocol and Review. Cochrane Handbook for Systematic Reviews of Interventions2008.
17. Sterne JA, Hernán MA, Reeves BC, et al. ROBINS-I: a tool for assessing risk of bias in non-randomised studies of interventions. *BMJ* 2016;355 doi: 10.1136/bmj.i4919
18. Review Manager (RevMan) [program]. Version 5.3 version. The Nordic Cochrane Centre: The Cochrane Collaboration, 2014.
19. Bavaria JE, Appoo JJ, Makaroun MS, et al. Endovascular stent grafting versus open surgical repair of descending thoracic aortic aneurysms in low-risk patients: a multicenter comparative trial. *Journal of Thoracic & Cardiovascular Surgery* 2007;133(2):369-77.
20. Cho JS, Haider SE, Makaroun MS. Endovascular therapy of thoracic aneurysms: Gore TAG trial results. *Seminars in Vascular Surgery* 2006;19(1):18-24.
21. Cho JS, Haider SE, Makaroun MS. US multicenter trials of endoprostheses for the endovascular treatment of descending thoracic aneurysms. *Journal of Vascular Surgery* 2006;43 Suppl A:12A-19A.
22. Go MR, Cho J-S, Makaroun MS. Mid-term Results of a Multicenter Study of Thoracic Endovascular Aneurysm Repair Versus Open Repair. *Perspectives in Vascular Surgery and Endovascular Therapy* 2007;19(2):124-30. doi: 10.1177/1531003507303430
23. Makaroun MS, Dillavou ED, Wheatley GH, et al. Five-year results of endovascular treatment with the Gore TAG device compared with open repair of thoracic aortic aneurysms. *Journal of Vascular Surgery* 2008;47(5):912-8.
24. Dillavou ED, Makaroun MS. Predictors of morbidity and mortality with endovascular and open thoracic aneurysm repair. *Journal of Vascular Surgery* 2008;48(5):1114-9; discussion 19-20.
25. Piffaretti G, Tozzi M, Lomazzi C, et al. Endovascular repair versus conventional surgery for descending thoracic aortic aneurysms. *Italian Journal of Vascular and Endovascular Surgery* 2007;14(4):279-85.
26. Goodney PP, Travis L, Lucas FL, et al. Survival after open versus endovascular thoracic aortic aneurysm repair in an observational study of the Medicare population. *Circulation* 2011;124(24):2661-9.
27. Hughes K, Guerrier J, Obirieze A, et al. Open versus endovascular repair of thoracic aortic aneurysms: a Nationwide Inpatient Sample study. *Vascular & Endovascular Surgery* 2014;48(5-6):383-7.
28. Von Allmen RS, Anjum A, Powell JT. Outcomes after endovascular or open repair for degenerative descending thoracic aortic aneurysm using linked hospital data. *British Journal of Surgery* 2014;101(10):1244-51.
29. Austin PC. An Introduction to Propensity Score Methods for Reducing the Effects of Confounding in Observational Studies. *Multivariate Behav Res* 2011;46(3):399-424. doi: 10.1080/00273171.2011.568786 [published Online First: 2011/06/08]
30. Bellomo R, Warrillow SJ, Reade MC. Why we should be wary of single-center trials. *Critical Care Medicine* 2009;37(12):3114-19. doi: 10.1097/CCM.0b013e3181bc7bd5
31. Eggebrecht H, Nienaber CA, Neuhäuser M, et al. Endovascular stent–graft placement in aortic dissection: a meta-analysis. *European Heart Journal* 2005;27(4):489-98. doi: 10.1093/eurheartj/ehi493



For peer review only - http://bmjopen.bmj.com/site/about/guidelines.xhtml

32. Alsawas M, Zaiem F, Larrea-Mantilla L, et al. Effectiveness of surgical interventions for thoracic aortic aneurysms: A systematic review and meta-analysis. *Journal of Vascular Surgery* 2017;66(4):1258-68.e8. doi: https://doi.org/10.1016/j.jvs.2017.05.082
33. Cheng D, Martin J, Shennib H, et al. Endovascular Aortic Repair Versus Open Surgical Repair for Descending Thoracic Aortic Disease: A Systematic Review and Meta-Analysis of Comparative Studies. *Journal of the American College of Cardiology* 2010;55(10):986-1001. doi: https://doi.org/10.1016/j.jacc.2009.11.047
34. Marko NF, Weil RJ. The Role of Observational Investigations in Comparative Effectiveness Research. *Value in Health* 2010;13(8):989-97. doi: https://doi.org/10.1111/j.1524-4733.2010.00786.x
35. Ludman Peter F, Moat N, de Belder Mark A, et al. Transcatheter Aortic Valve Implantation in the United Kingdom. *Circulation* 2015;131(13):1181-90. doi: 10.1161/CIRCULATIONAHA.114.013947
36. Katzen BT, Dake MD, MacLean AA, et al. Endovascular Repair of Abdominal and Thoracic Aortic Aneurysms. *Circulation* 2005;112(11):1663-75. doi: doi:10.1161/CIRCULATIONAHA.105.541284
37. Prasad A, Helder MR, Brown DA, et al. Understanding Differences in Administrative and Audited Patient Data in Cardiac Surgery: Comparison of the University HealthSystem Consortium and Society of Thoracic Surgeons Databases. *Journal of the American College of Surgeons* 2016;223(4):551-57.e4. doi: https://doi.org/10.1016/j.jamcollsurg.2016.06.393
38. Unverzagt S, Prondzinsky R, Peinemann F. Single-center trials tend to provide larger treatment effects than multicenter trials: a systematic review. *Journal of Clinical Epidemiology* 2013;66(11):1271-80. doi: 10.1016/j.jclinepi.2013.05.016

For peer review only



For peer review only - http://bmjopen.bmj.com/site/about/guidelines.xhtml

*Figure 1: PRISMA Diagram*

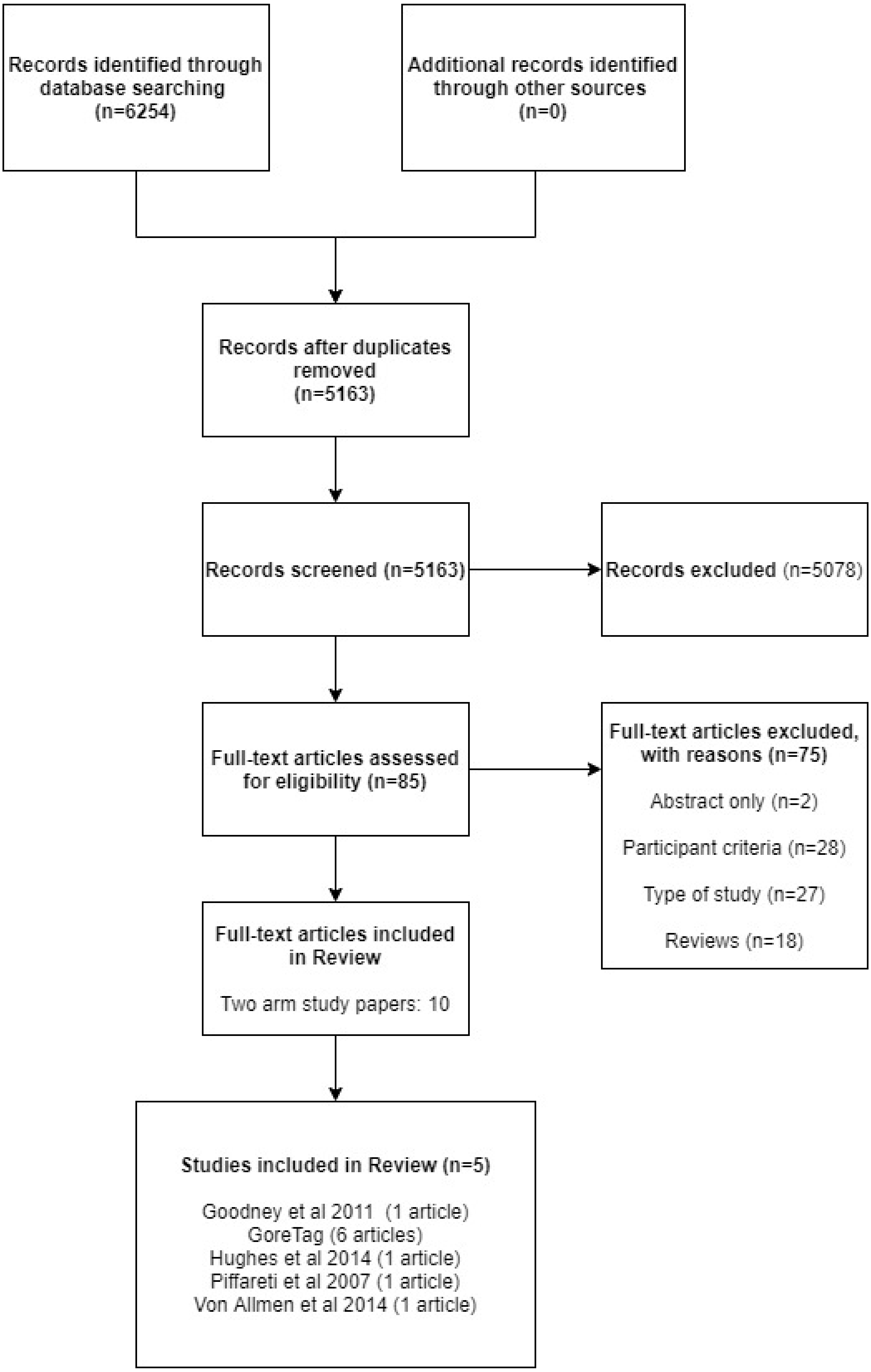

*Figure 2: Forest plot of unadjusted short-term all-cause mortality*

| Study or Subgroup | ESG Events | ESG Total | OSR Events | OSR Total | Weight | Odds Ratio M-H, Random, 95% CI |
|---|---|---|---|---|---|---|
| **1.1.1 Subgroup 1** | | | | | | |
| Goodney 2011 | 167 | 2732 | 893 | 12573 | 47.7% | 0.85 [0.72, 1.01] |
| Hughes 2014 | 26 | 712 | 380 | 8255 | 28.6% | 0.79 [0.52, 1.18] |
| Von Allmen 2014 | 23 | 354 | 20 | 264 | 17.2% | 0.85 [0.46, 1.58] |
| **Subtotal (95% CI)** | | **3798** | | **21092** | **93.4%** | **0.84 [0.72, 0.98]** |
| Total events | 216 | | 1293 | | | |
| Heterogeneity: Tau² = 0.00; Chi² = 0.13, df = 2 (P = 0.94); I² = 0% | | | | | | |
| Test for overall effect: Z = 2.22 (P = 0.03) | | | | | | |
| **1.1.2 Subgroup 2** | | | | | | |
| Gore TAG | 3 | 140 | 11 | 94 | 5.1% | 0.17 [0.04, 0.61] |
| Piffaretti 2007 | 1 | 17 | 2 | 11 | 1.5% | 0.28 [0.02, 3.55] |
| **Subtotal (95% CI)** | | **157** | | **105** | **6.6%** | **0.18 [0.06, 0.59]** |
| Total events | 4 | | 13 | | | |
| Heterogeneity: Tau² = 0.00; Chi² = 0.13, df = 1 (P = 0.71); I² = 0% | | | | | | |
| Test for overall effect: Z = 2.85 (P = 0.004) | | | | | | |
| **Total (95% CI)** | | **3955** | | **21197** | **100.0%** | **0.75 [0.55, 1.03]** |
| Total events | 220 | | 1306 | | | |
| Heterogeneity: Tau² = 0.04; Chi² = 6.72, df = 4 (P = 0.15); I² = 40% | | | | | | |
| Test for overall effect: Z = 1.80 (P = 0.07) | | | | | | |
| Test for subgroup differences: Chi² = 6.45, df = 1 (P = 0.01), I² = 84.5% | | | | | | |

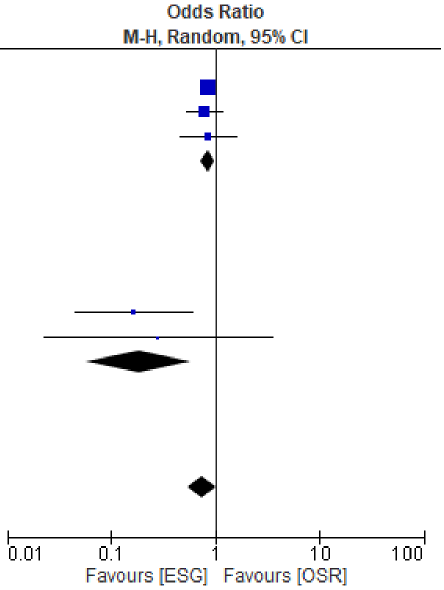


For peer review only

*Figure 3: Forest plot of unadjusted short-term all-cause mortality. Sensitivity Analysis.*

| | ESG | | OSR | | | Odds Ratio | Odds Ratio |
|---|---|---|---|---|---|---|---|
| Study or Subgroup | Events | Total | Events | Total | Weight | M-H, Random, 95% CI | M-H, Random, 95% CI |
| **1.1.1 large studies** | | | | | | | |
| Von Allmen 2014 | 23 | 354 | 20 | 264 | 30.9% | 0.85 [0.46, 1.58] | |
| Goodney 2011 | 167 | 2732 | 893 | 12573 | 58.4% | 0.85 [0.72, 1.01] | |
| **Subtotal (95% CI)** | | **3086** | | **12837** | **89.3%** | **0.85 [0.72, 1.00]** | |
| Total events | 190 | | 913 | | | | |
| Heterogeneity: Tau² = 0.00; Chi² = 0.00, df = 1 (P = 0.99); I² = 0% | | | | | | | |
| Test for overall effect: Z = 1.92 (P = 0.06) | | | | | | | |
| **1.1.2 Small studies** | | | | | | | |
| Piffaretti 2007 | 1 | 17 | 11 | 94 | 4.8% | 0.47 [0.06, 3.91] | |
| Gore TAG | 3 | 140 | 2 | 11 | 5.8% | 0.10 [0.01, 0.67] | |
| **Subtotal (95% CI)** | | **157** | | **105** | **10.7%** | **0.20 [0.04, 1.06]** | |
| Total events | 4 | | 13 | | | | |
| Heterogeneity: Tau² = 0.37; Chi² = 1.35, df = 1 (P = 0.25); I² = 26% | | | | | | | |
| Test for overall effect: Z = 1.89 (P = 0.06) | | | | | | | |
| **Total (95% CI)** | | **3243** | | **12942** | **100.0%** | **0.73 [0.45, 1.18]** | |
| Total events | 194 | | 926 | | | | |
| Heterogeneity: Tau² = 0.10; Chi² = 5.14, df = 3 (P = 0.16); I² = 42% | | | | | | | |
| Test for overall effect: Z = 1.28 (P = 0.20) | | | | | | | |
| Test for subgroup differences: Chi² = 2.87, df = 1 (P = 0.09), I² = 65.1% | | | | | | | |

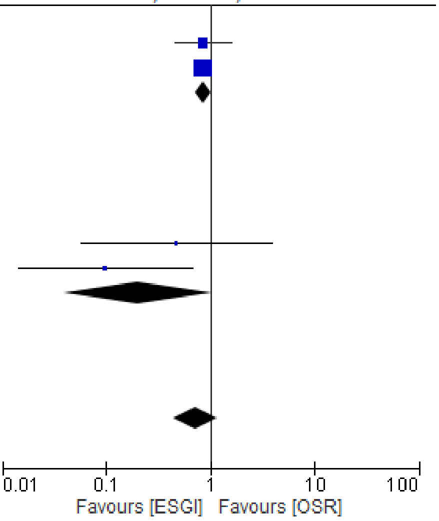


peer review only

*Figure 4: Forest plot of adjusted short-term all-cause mortality*

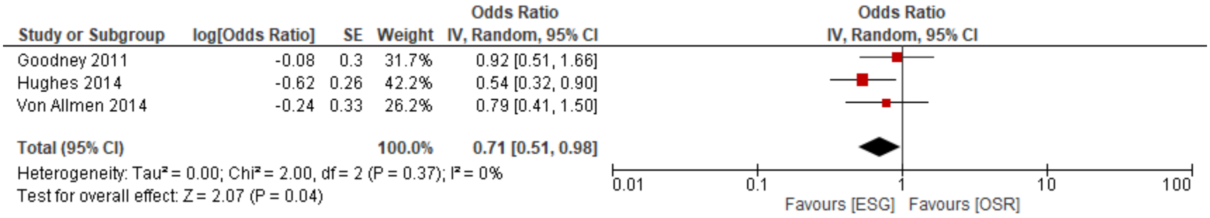


peer review only

## Appendix 1: Search Strategy

### Medline/Central/HTA

1. (TEVAR or EVAR).ti,ab.
2. (endovascular adj1 (stent* or repair* or treat* or surg* or (aneurysm? adj1 repair*))).ti,ab.
3. (open adj2 (surg* or repair*)).ti,ab.
4. endoprosthe*.mp.
5. *Aortic Aneurysm, Thoracic/su [Surgery]
6. *Aortic Thoracic/su [Surgery]
7. Thoracic Surgical Procedures/
8. or/1-7
9. exp Aortic Aneurysm, Thoracic/
10. (thorac* adj2 aneurysm*).ti,ab.
11. (descending adj1 aort* adj1 aneurysm*).ti,ab
12. TAA*.ti,ab.
13. (arch adj3 aneurysm*).ti.ab.
14. or/9-13
15. morbid*.mp.
16. mortalit*.mp.
17. outcome?.mp.
18. complication*.mp.
19. (rupture? or endoleak?).mp.
20. device migration.mp.
21. (ae or co or de).fs

For peer review only

22. (safe or safety or side effect* or undesirable effect* or treatment emergent or tolerability or toxicity or adrs or (adverse adj2 (effect or effects or reaction or reactions or event or events to outcome or outcomes))).ti,ab.
23. ((technical or surg*) adj2 (advance* or improve*)).mp.
24. (quality adj2 life).mp.
25. Mortality/
26. Mortbidity/
27. Postoperative complications/
28. Intraoperative complications/
29. Aortic Aneurysm, Thoracic/co
30. Conversion to Open Surgery/
31. "Quality of Life"
32. or/15-31
33. 8 and 14 and 32
34. ("clinical trial" or "clinical trial, phase I" or "clinical trial, phase ii" or clinical trial phase iii or clinical trial, phase iv or controlled clinical trial or "multicentre study" or "randomized controlled trial").pt. or double-blind method/ or clinical trials, phase iii as topic/ or clinical trials, phase iv as topic/ or controlled clinical trials as topic/ or randomized controlled trials as topic/ or early termination of clincal trials as topic/ or multicenter studies as topic/ or ((randomi?ed adj7 trial*) or (controlled adj3 trial*) or (clinical adj2 trial*) or ((single or doubl* or tripl* or treb*) and (blind* or mask*))).ti,ab,kw. or ("4 arm" or "four arm").ti,ab,kw.
35. cohort studies/ or longitudinal studies/ or follow-up studies/ or prospective studies/ or retrospective studies/ or cohort.ti,ab. or longitudinal.ti,ab. or prospective.ti,ab. or retrospective.ti,ab. or quasi-experiment.ti,ab.

For peer review only

36. Clinical Trial.pt. or random*.tw. or Clinical Trials/ or clinical trial.tw. or Follow-up Studies/ or prospectiv*.tw. or control*.tw.
37. or/34-36
38. 33 and 37

EMBASE

1. (TEVAR or EVAR).ti,ab.
2. (endovascular adj1 (stent* or repair* or treat* or surg* or (aneurysm? adj1 repair*))).ti,ab.
3. (open adj2 (surg* or repair*)).ti,ab.
4. endoprosthe*.mp.
5. Thoracic Aorta Aneruysm/su [Surgery]
6. Thoracoabdominal aorta aneurysm/su [Surgery]
7. Thoracic Aorta/su [Surgery]
8. exp Thoracic aorta surgery/
9. or/1-8
10. exp Thoracic Aorta Aneurysm/
11. exp Thoracoabdominal aorta aneurysm/
12. Thoracic Aorta/
13. (thorac* adj2 aneurysm*).ti,ab.
14. (descending adj1 aort* adj1 aneurysm*).ti,ab.
15. TAA*.ti,ab.
16. (arch adj3 aneurysm*).ti,ab.
17. or/10-16
18. (outcome? or mortalit*).mp.
19. complication*.mp.
20. ((technical or surg*) adj2 (advance* or improve*)).mp.

For peer review only

21. (quality adj2 life).mp.
22. Mortality/
23. Postoperative complication/
24. peroperative complication/
25. Thoracic Aorta Aneurysm/co
26. “Quality of Life”/
27. or/18-26
28. (Randomised Controlled Trial or Pragmatic Clinical Trial).pt or exp Randomized Controlled Trials as Topic/ or “Randomized Controlled Trial (topic)”/ or Randomized Controlled Trial/ or Randomization/ or Random Allocation/ or Double-Blind Method/ or Double Blind Procedure/ or Double-Blind Studies/ or Single-Blind Method/ or Single Blind Procedure/ or Single-Blind Studies/ or Placebos/ or Placebo/ or (random* or sham or placebo*).ti,ab,hw,kf,kw. or ((singl* or doubl*) adj (blind* or dumm* or mask*)).ti,ab,hw,,kw. or ((tripl* or trebl*) adj (blind* or dumm* or mask*)).ti,ab,hw,,kw.
29. random:.tw. or placebo:.mp. or double-blind:.mp.
30. controlled clinical trial/ or clinical trial/ or exp controlled study/ or randomized controlled trial/ or exp quasi experimental study/
31. or/28-30
32. 9 and 17 and 27 and 31
33. limit 32 to (human and yr=”1994-Current”)
34. exp cohort analysis/ or exp longitudinal study/ or exp prospective study/ or exp follow up/ or cohort*.tw.
35. limit 34 to (human and yr=”1994-Current”)
36. 9 and 17 and 27 and 35
37. 36 not 33
38. 33 or 37

For peer review only

**Appendix 2: Data extraction table**

| Study | **Von Allmen et al 2014** | **Goodney et al 2011** (2 analyses*) | **Gore TAG** | **Hughes et al 2014** | **Piffaretti et al 2007** |
|---|---|---|---|---|---|
| **METHODS** | | | | | |
| **Study Design** | Comparative cohort | Comparative cohort | Comparative cohort | comparative cohort | Comparative cohort |
| **Method of matching** | None | 1. None<br>2. propensity matched | ESG patients judged eligible for OSR | None | OSR patients judged eligible for ESG |
| **Inclusion Criteria** | Hospital diagnosis code 171.2 (TAA without mention of rupture) from ICD-10 score linked to | Patients diagnosed with a thoracic aneurysm according to ICD9 diagnosis code: | Fusiform descending thoracic aortic aneurysm at least twice the size of the normal adjacent aorta or saccular | Nationwide inpatient Sample database of patients >18 years old who had | Elective, Chronic fusiform or saccular aneurysm of the descending thoracic aorta, OSR patients |

For peer review only

| | | | | | |
|---|---|---|---|---|---|
| | operations code according to OPCS-4, linked with all-cause mortality from ONS and ICD-10, only OPCS-4 codes: L18.2, L19.2, L20.2, L21.2 | Intact thoracic aneurysms 441.2<br>Patients had undergone an open or TEVAR procedure according to ICD9 procedure codes:<br>Open 38.35, 38.45<br>TEVAR 39.73, 39.79 | aneurysm, Life expectancy >2 years<br>Surgical candidate<br>Male or infertile female >21 years old<br>Descending aorta must be clampable distal to the left carotid artery, and distal anastomosis must be performed proximal to the celiac axis<br>Inner aortic diameter of 23-37mm adjacent to aneurysm<br>Lack of significant thrombus of calcification in landing zones | undergone an open or endovascular repair of non-ruptured thoracic aortic aneurysm. Patients selected using ICD-9 procedures codes 39.73 and 38.45. | would have been a candidates for ESG due to morphological criteria, Selected on surgeon's opinion of lower risk of ESG |

For peer review only

| | | | | | |
|---|---|---|---|---|---|
| | | | Minimum of 2cm of normal thoracic aorta proximal and distal to aneurysm Aortic taper of no more than 4mm or ability to treat with more than one graft | | |
| **Exclusion Criteria** | Dissection, Abdominal aneurysms, Non-identified area aneurysm, Ascending aneurysm, Suprarenal aneurysm, Non- | Patients with ICD 9 diagnosis codes indicating ascending thoracic aneurysms or cardiopulmonary bypass occurring with circulatory arrest. Thoracoabdominal aneurysm, thoracic aortic | Mycotic aneurysm, Hemodynamically unstable ruptured aneurysm , Major operation (other than planned subclavian to carotid transposition or bypass) within 30 days, MI or CVA within 6 weeks, Creatinine >2.0mg/dL, Connective tissue | NA | Traumatic, Mycotic thoracic aneurysms, Dissections, Aneurysms of the ascending aorta, Aneurysms of the aortic arch, Aortic ulcers |

For peer review only

| | | | | | |
|---|---|---|---|---|---|
| | aortic procedures, age <50yrs | dissection, other aortic pathology. IC9 procedure codes indicating debranching or other procedures to extend endovascular landing zones (39.24, 39.25, | disorder, Acute or chronic aortic dissection, Planned occlusion of carotid or celiac arteries, Documented drug abuse within 6 months, Participation in another investigational device or drug study within 1 year | | |
| **Definition of the short-term** | 30 days post-procedure | latest occurring of discharge or 30 days post-intervention | latest occurring of discharge or 30 days post-intervention | post-procedure hospital stay | 30-days post-intervention |
| **PARTICIPANTS** | | | | | |
| **Country** | UK | USA | USA | USA | Italy |

| | | | | | |
|---|---|---|---|---|---|
| **Setting** | Multi-centre – Hospital episode statistics | Multi-centre – Medicare data | Multi-centre | Multi-centre | Single-centre |
| **Time frame** | 2006 to 2011 | 1. 1998-2007<br>2. 2003-2007 | ESG 1999- 2001, OSR pre 1999 (n=44) & 1999-2001 (n=50) | 1998-2007 | ESG 2000-2007, OSR 1996-2000 |
| **Recruitment** | Total 618<br>ESG 354<br>OSR 264 | 1. Total 13,998 (ESG 2433, OSR 11,565)<br>2. Total 1100 (ESG 550, OSR 550) | Total 234<br>ESG 140<br>OSR 94 | Total 8967<br>ESG 712<br>OSR 8255 | Total 28<br>ESG 17<br>OSR 11 |
| **Age summary** | median (IQR)<br>ESG 73(66,78), OSR 71 (63-76) | mean (95% C.I.)<br>1. ESG 75.9 (75.6, 76.1), OSR 73.8 (73.7, 73.9)<br>2.<br>ESG 71.1 (70.8, 71.44), OSR 70.7 (70.7, 71.1) | mean(SD)<br>ESG 75.9 (10.4), OSR 68.2 (10.2) | median (IQR)<br>ESG 72 (63- 78), OSR 63 (52, 72) | mean (SD)<br>ESG 66 (10), OSR 61 (13) |

For peer review only

| | | | | | |
|---|---|---|---|---|---|
| **Proportion male** | ESG 232 (65.5%), OSR 137 (51.9%) | % (95% C.I.): 1. ESG 58.7 (56.7, 60.7), OSR 55.4 (54.8, 56.3) 2. ESG 64.0 (59.9, 68.0), OSR 68.1% (64.2, 72.0) | ESG 90(57%), OSR 48 (51%) | 5415 (61.3%) ESG, 437 (65.6%) OSR | ESG 14 (82%), OSR 8 (73%) |
| **Black race** | NR | % (95% C.I.): 1. ESG 7.5 (6.4, 8.5), OSR 3.8 (3.4, 4.5) (p=0.001) 2. ESG 2.7 (1.4, 4.1), OSR 2.7 (1.4, 4.0) (p=0.99) | NR | NR | NR |
| **Charlson comorbidity score** | NR | % (95% C.I.): 1. ESG 1.46 (1.38, 1.54), OSR 0.83 (0.80, 0.86) (p=0.00001) | NR | NR | NR |

For peer review only

| | | | | | |
|---|---|---|---|---|---|
| | | 2. ESG 0.5 (0.41, 0.60), OSR 0.44 (0.35, 0.53) (p=0.34) | | | |
| **Diabetes** | NR | % (95% C.I.): 1. ESG 8.00 (6.7, 8.8), OSR 4.10 (3.7, 4.5) (p=0.001) 2. ESG 4.90 (3.0, 6.7), OSR 2.90 (1.5, 4.3) (p=0.09) | NR | 684 (13.2%) ESG, 94 (8.3%) OSR (p<0.001) | NR |
| **Myocardial infarction** | NR | % (95% C.I.): 1. ESG 7.00 (6.00, 8.1), OSR 5.10 (4.6, 5.4) (p=0.001) 2. ESG 2.50 (1.2, 3.8), OSR 3.40 (1.9, 4.9) (p=0.38) | -NR | NR | NR |
| **Congestive heart failure** | NR | % (95% C.I.): | ESG 13 (9%), OSR 9 (10%) (p=1.00) | NR | NR |

For peer review only

| | | | | | |
|---|---|---|---|---|---|
| | | 1. ESG 10.85% (9.6%-12.0%), OSR 10.60% (10.1%- 11.2%) (p=0.16)<br>2. ESG 3.00 (1.6, 4.5), OSR 4.70 (2.9, 6.5) (p=0.16) | | | |
| **Cerebrovascular disease** | NR | % (95% C.I.):<br>1. ESG 5.09% (4.2%-5.9%), OSR 3.20% (2.9%- 3.5%) (p=0.65)<br>2. ESG 2.00 (0.5, 2.6), OSR 2.00 (0.8, 3.1) (p=0.65) | NR | NR | 4 (23%) ESG, 1 (9%) OSR (p=NS) |
| **COPD** | NR | % (95% C.I.):<br>1. ESG 22.04% (20.3%-23.6%), OSR 10.65% (10.0%-11.2%) (p=0.47) | ESG 56 (40%), OSR 36 (38%) (p=0.89) | 247 (34.7%) ESG, 1483 (17.9%) (P<0.001) | 10 (59%) ESG, 6 (54%) OSR (p=NS) |

For peer review only

| | | | | | |
|---|---|---|---|---|---|
| | | 2. ESG 4.00 (2.3, 5.6), OSR 4.90 (3.0, 6.7) (p=0.47) | | | |
| **Chronic renal failure** | NR | % (95% C.I.):<br>1. ESG 4.9% (4.0%- 5.7%), OSR 0.88% (0.07%- 0.10%) (p=0.70)<br>2. ESG 0.01 (0, 1.4), OSR 0.50 (0, 1.1) (p=0.70) | NR | 90 (12.6%) ESG, 126 (1.5%) OSR (p<0.001) | 4 (23%) ESG, 1 (9%) OSR (p=NS) |
| **History of malignancy** | NR | % (95% C.I.):<br>1. ESG 5.71% (4.7%-6.6%), OSR 2.91% (2.6%- 3.2%) (p=0.82)<br>2. ESG 1.80 (0.6, 2.9), OSR 1.60 (0.5, 2.6) (p=0.82) | NR | NR | NR |
| **History of smoking (current or past)** | NR | NR | ESG 117 (84%), OSR 77 (82%) (p=0.86) | NR | NR |

| | | | | | |
|---|---|---|---|---|---|
| **BMI (kg/m2)** | NR | NR | mean (SD): ESG 26.4 (4.7), OSR 26.9 (5) (p=0.44) | NR | NR |
| **Coronary artery disease** | NR | NR | ESG 69 (49%), OSR 34 (36%) (p=0.60) | NR | NR |
| **Stroke** | NR | NR | ESG 14 (10%), OSR 9 (9%) (p=1.00) | NR | NR |
| **Peripheral arterial disease** | NR | NR | ESG 22 (16%), OSR 10 (11%) (p=0.33) | NR | NR |
| **Symptomatic aneurysm** | NR | NR | ESG 30 (21%), OSR 36 (38%) (p=0.007) | NR | NR |
| **Other concomitant aneurysm:** | NR | NR | ESG 39 (28%), OSR 26 (28%) (p=1.00) | NR | NR |
| **Renal dialysis** | NR | NR | ESG 2 (1%), OSR 0 (0%) (p=0.52) | NR | NR |

For peer review only

| | | | | | |
|---|---|---|---|---|---|
| **Paraplegia** | NR | NR | ESG 1 (1%), OSR 0 (0%) (p=1.00) | NR | NR |
| **Cancer** | NR | NR | ESG 27 (19%), OSR 12 (13%) (p=0.21) | NR | NR |
| **Prior major vascular surgery** | NR | NR | ESG 63 (45%), OSR 52 (55%) (p=0.14) | NR | NR |
| **Cardiac comorbidities** | NR | NR | NR | 265 (37.2%) ESG, 3535 (42.8%) (p<0.001) | NR |
| **Hypertension** | NR | NR | NR | NR | 16 (94%) ESG, 10 (91%) OSR (p=NS) |
| **Peripheral obstructive disease** | NR | NR | NR | NR | 5 (29%) ESG, 2 (18%) OSR (p=NS) |
| **Ischemic heart disease** | NR | NR | NR | NR | 6 (35%) ESG, 5 (45%) OSR (p=NS) |

For peer review only

| | | | | | |
|---|---|---|---|---|---|
| **Cardiac Surgery** | NR | NR | NR | NR | (18%) ESG, 0 (0%)<br>OSR (p=NS) |
| **SHORT- TERM OUTCOMES** | | | | | |
| **All-cause mortality** | ESG 21 (5.9%)<br>OSR 4 (1.5%)<br>Adjusted OR: 0.79 | 1. ESG 6.1% (95%CI 5.1%, 7.0%)<br>OSR 7.1% (95%CI 6.7%, 7.6%)<br>2. ESG 4.2% (95% C.I. 2.5%-5.8%)<br>OSR 4.5% (95% C.I. 2.8%-6.2%) | ESG 3 (2.1%)<br>OSR 11 (11.7%)<br>Adjusted OR: 0.54 | ESG 25<br>OSR 379 | ESG 1 (6%)<br>OSR 2 (20%) |
| **Re-intervention** | ESG 21 (5.9%)<br>OSR 4 (1.5%)<br>Adjusted OR: 2.97 (1.09, 8.10) | NR | NR | NR | NR |

For peer review only

| | | | | | |
|---|---|---|---|---|---|
| **Paraplegia/ Paraperisis** | NR | NR | ESG 4 (3%)<br>OSR 13 (14%) | NR | NR |
| **Cerebral Vascular Accident** | NR | NR | ESG 5 (4%)<br>OSR 5 (4%) | NR | ESG 2 (9%)<br>OSR 1 (12%) |
| **Neurological-unspecified type** | NR | NR | NR | ESG 20 (2.8%)<br>OSR 273 (3.3%)<br>Adjusted OR: 0.38 | NR |
| **Respiratory failure** | NR | NR | ESG 5 (4%)<br>OSR 19 (20%) | NR | NR |
| **Pneumonia** | NR | NR | NR | NR | ESG 2 (12%)<br>OSR 3 (27%) |
| **Pulmonary-unspecified type** | NR | NR | NR | ESG 17 (2.4%)<br>OSR 462 (5.6%)<br>Adjusted OR: 0.48 | NR |
| **Myocardial Infarction** | NR | NR | ESG 0 (0%)<br>OSR 1(1%) | NR | ESG 0 (0%)<br>OSR 1 (9%) |

For peer review only

| | | | | | |
|---|---|---|---|---|---|
| **Cardiac-unspecified type** | NR | NR | NR | ESG 28 (2.9%)<br>OSR 1252 (15.2%)<br>Adjusted OR: 0.24 | NR |
| **Peripheral vascular disease** | NR | NR | ESG 20 (14%)<br>OSR 4 (4%) | NR | NR |
| **Renal failure** | NR | NR | ESG 2 (1%)<br>OSR 12 (13%) | NR | NR |
| **Wound infection/dehiscence** | NR | NR | ESG 5 (4%)<br>OSR 10 (11%) | NR | NR |
| **GI complication** | NR | NR | ESG 3 (2%)<br>OSR 6 (6%) | NR | NR |
| **Post-implant syndrome** | NR | NR | NR | NR | ESG 4(18%)<br>OSR 0(0%) |
| **LONG-TERM OUTCOMES** | | | | | |

For peer review only

| | | | | | |
|---|---|---|---|---|---|
| All-cause survival | 1 year adjusted OR: 1.1 95%CI [0.70-1.73], p=0.667 5 year adjusted HR: 1.45 95%CI [1.08-1.94], p=0.013 | NR | NR | NR | NR |
| **aortic-related survival** | 5 year adjusted HR: 1.27 95%CI [0.81-1.98], p=0.298 | NR | NR | NR | NR |
| **survival from aortic-related re-intervention** | 5 year adjusted HR: 1.70 95%CI [1.11-2.60], p=0.014 | NR | NR | NR | NR |
| * 1. Unadjusted cohort, 2. Propensity matched cohort | | | | | |

For peer review only

**Appendix 3: Risk of Bias assessment**

*Risk of Bias Results using the ROBINS-I risk of bias tool*

| Domain – source of bias | Goodney et al 2011 (2 analyses*) | Gore TAG | Hughes et al 2014 | Piffaretti et al 2007 | Von Allmen et al 2014 |
|---|---|---|---|---|---|
| **Confounding** | 1. Severe<br>2. Moderate | Severe | Severe | Severe | Severe |
| **Selection of participants into the study** | 1. Low<br>2. Low | Low | Low | Low | Low |
| **Classification of interventions** | 1. Low<br>2. Low | Low | Low | Low | Low |
| **Deviations from intended interventions** | 1. Low<br>2. Low | Low | Low | Low | Low |
| **Missing data** | 1. Low<br>2. Low | Low | Low | Low | Low |
| **Measurement of outcomes** | 1. Low<br>2. Low | Low | Low | Low | Low |
| **Bias in selection of the reported result** | 1. Moderate<br>2. Moderate | Moderate | Moderate | Moderate | Moderate |
| **Overall** | 1. Severe<br>2. Moderate | Severe | Severe | Severe | Severe |
| * 1. Unadjusted cohort, 2. Propensity matched cohort | | | | | |

For peer review only

| Section/topic | # | Checklist item | Reported on page # |
|---|---|---|---|
| **TITLE** | | | |
| Title | 1 | Identify the report as a systematic review, meta-analysis, or both. | 1 |
| **ABSTRACT** | | | |
| Structured summary | 2 | Provide a structured summary including, as applicable: background; objectives; data sources; study eligibility criteria, participants, and interventions; study appraisal and synthesis methods; results; limitations; conclusions and implications of key findings; systematic review registration number. | 2 |
| **INTRODUCTION** | | | |
| Rationale | 3 | Describe the rationale for the review in the context of what is already known. | 4 |
| Objectives | 4 | Provide an explicit statement of questions being addressed with reference to participants, interventions, comparisons, outcomes, and study design (PICOS). | 5 |
| **METHODS** | | | |
| Protocol and registration | 5 | Indicate if a review protocol exists, if and where it can be accessed (e.g., Web address), and, if available, provide registration information including registration number. | 6 |
| Eligibility criteria | 6 | Specify study characteristics (e.g., PICOS, length of follow-up) and report characteristics (e.g., years considered, language, publication status) used as criteria for eligibility, giving rationale. | 6 |
| Information sources | 7 | Describe all information sources (e.g., databases with dates of coverage, contact with study authors to identify additional studies) in the search and date last searched. | 7 |
| Search | 8 | Present full electronic search strategy for at least one database, including any limits used, such that it could be repeated. | 7 |
| Study selection | 9 | State the process for selecting studies (i.e., screening, eligibility, included in systematic review, and, if applicable, included in the meta-analysis). | 8 |
| Data collection process | 10 | Describe method of data extraction from reports (e.g., piloted forms, independently, in duplicate) and any processes for obtaining and confirming data from investigators. | 8 |

For peer review only

| | | | |
|---|---|---|---|
| Data items | 11 | List and define all variables for which data were sought (e.g., PICOS, funding sources) and any assumptions and simplifications made. | 8 |
| Risk of bias in individual studies | 12 | Describe methods used for assessing risk of bias of individual studies (including specification of whether this was done at the study or outcome level), and how this information is to be used in any data synthesis. | 8 |
| Summary measures | 13 | State the principal summary measures (e.g., risk ratio, difference in means). | 9 |
| Synthesis of results | 14 | Describe the methods of handling data and combining results of studies, if done, including measures of consistency (e.g., $I^2$) for each meta-analysis. | 9 |



| Section/topic | # | Checklist item | Reported on page # |
|---|---|---|---|
| Risk of bias across studies | 15 | Specify any assessment of risk of bias that may affect the cumulative evidence (e.g., publication bias, selective reporting within studies). | 9 |
| Additional analyses | 16 | Describe methods of additional analyses (e.g., sensitivity or subgroup analyses, meta-regression), if done, indicating which were pre-specified. | 9 |
| **RESULTS** | | | |
| Study selection | 17 | Give numbers of studies screened, assessed for eligibility, and included in the review, with reasons for exclusions at each stage, ideally with a flow diagram. | 9 |
| Study characteristics | 18 | For each study, present characteristics for which data were extracted (e.g., study size, PICOS, follow-up period) and provide the citations. | Table 1, appendix 2 |
| Risk of bias within studies | 19 | Present data on risk of bias of each study and, if available, any outcome level assessment (see item 12). | 14, appendix 3 |
| Results of individual studies | 20 | For all outcomes considered (benefits or harms), present, for each study: (a) simple summary data for each intervention group (b) effect estimates and confidence intervals, ideally with a forest plot. | 14-19 |
| Synthesis of results | 21 | Present results of each meta-analysis done, including confidence intervals and measures of consistency. | 14-19 |
| Risk of bias across studies | 22 | Present results of any assessment of risk of bias across studies (see Item 15). | 14-19 |
| Additional analysis | 23 | Give results of additional analyses, if done (e.g., sensitivity or subgroup analyses, meta-regression [see Item 16]). | NA |

For peer review only

| | | | |
|---|---|---|---|
| **DISCUSSION** | | | |
| Summary of evidence | 24 | Summarize the main findings including the strength of evidence for each main outcome; consider their relevance to key groups (e.g., healthcare providers, users, and policy makers). | 19-21 |
| Limitations | 25 | Discuss limitations at study and outcome level (e.g., risk of bias), and at review-level (e.g., incomplete retrieval of identified research, reporting bias). | 21 |
| Conclusions | 26 | Provide a general interpretation of the results in the context of other evidence, and implications for future research. | 22 |
| **FUNDING** | | | |
| Funding | 27 | Describe sources of funding for the systematic review and other support (e.g., supply of data); role of funders for the systematic review. | 22 |

*From:* Moher D, Liberati A, Tetzlaff J, Altman DG, The PRISMA Group (2009). Preferred Reporting Items for Systematic Reviews and Meta-Analyses: The PRISMA Statement. PLoS Med 6(7): e1000097. doi:10.1371/journal.pmed1000097

For more information, visit: **www.prisma-statement.org**.



For peer review only

For peer review only - http://bmjopen.bmj.com/site/about/guidelines.xhtml

BMJ Open

# A Systematic Review of Endovascular Stent Grafting Versus Open Surgical Repair for the Elective Treatment of Arch/Descending Thoracic Aortic Aneurysms


| | |
|---|---|
| Journal: | *BMJ Open* |
| Manuscript ID | bmjopen-2020-043323.R2 |
| Article Type: | Original research |
| Date Submitted by the Author: | 09-Feb-2021 |


| | |
|---|---|
| Complete List of Authors: | McCarthy, Andrew; Northumbria University, Faculty of Health and Life Sciences<br>Gray, Joanne; Northumbria University, Health and Life Sciences<br>Sastry, Priya; John Radcliffe Hospital, Cardiothoracic Surgery<br>Sharples, Linda; London School of Hygiene and Tropical Medicine, Medical Statistics<br>Vale, Luke; Newcastle University, Health Economics Group, Population Health Sciences Institute<br>Cook, Andrew; University of Southampton, Wessex Institute; University of Southampton, Southampton Clinical Trials Unit<br>Mcmeekin, Peter; Northumbria University, Faculty of Health and Life Sciences<br>Freeman, Carol; Papworth Hospital NHS Foundation Trust, Papworth Trials Unit Collaboration<br>Catarino, Pedro; Papworth Hospital NHS Foundation Trust<br>Large, Stephen; Papworth Hospital NHS Foundation Trust, Cardiac Surgery |


| | |
|---|---|
| <b>Primary Subject Heading</b>: | Surgery |
| Secondary Subject Heading: | Surgery |
| Keywords: | Cardiothoracic surgery < SURGERY, Vascular surgery < SURGERY, Thoracic surgery < SURGERY |
| | |


SCHOLARONE™ Manuscripts

*I, the Submitting Author has the right to grant and does grant on behalf of all authors of the Work (as defined in the below author licence), an exclusive licence and/or a non-exclusive licence for contributions from authors who are: i) UK Crown employees; ii) where BMJ has agreed a CC-BY licence shall apply, and/or iii) in accordance with the terms applicable for US Federal Government officers or employees acting as part of their official duties; on a worldwide, perpetual, irrevocable, royalty-free basis to BMJ Publishing Group Ltd ("BMJ") its licensees and where the relevant Journal is co-owned by BMJ to the co-owners of the Journal, to publish the Work in this journal and any other BMJ products and to exploit all rights, as set out in our licence.*

*The Submitting Author accepts and understands that any supply made under these terms is made by BMJ to the Submitting Author unless you are acting as an employee on behalf of your employer or a postgraduate student of an affiliated institution which is paying any applicable article publishing charge ("APC") for Open Access articles. Where the Submitting Author wishes to make the Work available on an Open Access basis (and intends to pay the relevant APC), the terms of reuse of such Open Access shall be governed by a Creative Commons licence – details of these licences and which Creative Commons licence will apply to this Work are set out in our licence referred to above.*

*Other than as permitted in any relevant BMJ Author's Self Archiving Policies, I confirm this Work has not been accepted for publication elsewhere, is not being considered for publication elsewhere and does not duplicate material already published. I confirm all authors consent to publication of this Work and authorise the granting of this licence.*

For peer review only

# A Systematic Review of Endovascular Stent Grafting Versus Open Surgical Repair for the Elective Treatment of Arch/Descending Thoracic Aortic Aneurysms

Andrew McCarthy [1], Joanne Gray [1], Priya Sastry [2], Linda Sharples [3], Luke Vale [4], Andrew Cook [5,6,7]; Peter McMeekin [1], Carol Freeman [8], Pedro Catarino [9], Stephen Large [9]

[1] Faculty of Health and Life Sciences, Northumbria University, Newcastle Upon Tyne, UK

[2] John Radcliffe Hospital, Oxford University Hospitals, UK

[3] Department of Medical Statistics, London School of Hygiene and Tropical Medicine, London, UK

[4] Health Economics Group, Population Health Sciences Institute, Newcastle University, Newcastle Upon Tyne, UK

[5] Wessex Institute, University of Southampton, UK

[6] Southampton Clinical Trials Unit, University of Southampton, UK

[7] University Hospital Southampton NHS Foundation Trust, UK

[8] Papworth Trials Unit Collaboration, Royal Papworth Hospital NHS Foundation Trust, Cambridge

[9] Royal Papworth Hospital NHS Foundation Trust, Cambridge

**Corresponding Author:**

**Andrew McCarthy-** Coach Lane Campus West, Northumbria University, Newcastle upon Tyne, United Kingdom, NE7 7XA

andrew2.mccarthy@northumbria.ac.uk

Tele: 0191 215 6718

For peer review only

**ABSTRACT**

**Objective:** to review comparisons of the effectiveness of endovascular stent grafting (ESG) against open surgical repair (OSR) for treatment of chronic arch or descending thoracic aortic aneurysms (TAA).

**Design** Systematic review and meta-analysis

**Data sources** MEDLINE, EMBASE, CENTRAL, WHO International Clinical Trials Routine data collection, current controlled trials, clinical trials, and the NIHR portfolio were searched from January 1994 through March 2020.

**Eligibility criteria for selective studies** All identified studies that compared ESG and OSR, including RCTs, quasi-randomized and non-randomized controlled trials, comparative cohort studies and case-control studies matched on main outcomes were sought. Participants had to receive elective treatments for arch/descending (TAA). Studies were excluded where other thoracic aortic conditions (e.g. rupture or dissection) were reported, unless results for patients receiving elective treatment for arch/descending TAA reported separately.

**Data extraction and synthesis** Data was extracted by one reviewer and checked by another. Risk of Bias was assessed using the ROBINS-I tool. Meta-analysis was conducted using random effects. Where meta-analysis not appropriate, results were reported narratively.

**Results** Five comparative cohort studies met inclusion criteria, reporting 3955 ESG and 21,197 OSR patients. Meta-analysis of unadjusted short-term (30 day) all-cause mortality favoured ESG (OR: 0.75; 95%CI (0.55, 1.03)). Heterogeneity identified between larger and smaller studies. Sensitivity analysis of four studies including only descending TAA showed no statistical significance (OR 0.73, 95 %CI. (0.45, 1.18)), moderate heterogeneity. Meta-analysis of adjusted short-term all-cause mortality favoured ESG (OR 0.71, 95%CI (0.51, 0.98)), no heterogeneity. Longer-term (beyond 30 days) survival from all-cause mortality favoured OSR in larger studies and ESG in smaller studies. Freedom from re-




For peer review only - http://bmjopen.bmj.com/site/about/guidelines.xhtml

intervention in the longer-term favoured OSR. Studies reporting short-term non-fatal complications suggest fewer events following ESG.

**Conclusions** There is limited and increasingly dated evidence on the comparison of ESG and OSR for treatment of arch/descending TAA

**PROSPERO registration number** CRD42017054565

**KEY WORDS**

Systematic Review, Meta-analysis, Thoracic Aortic Aneurysm, Open Surgical Repair, Endovascular Stent Graft

**STRENGTHS AND LIMITATIONS OF THIS STUDY**

Strength:

- This is the first systematic review to investigate two-armed studies looking only at elective ESG versus OSR for treatment of chronic arch or descending thoracic aortic aneurysms. Other reviews include dissections, ruptures and treatment for other conditions.
- This systematic review inclusion criteria were broad and sought to include evidence from both randomized and non-randomized study designs.

Limitation:

- Statistical analysis of non-fatal complications was not possible due to the limited and inconsistent reporting of such complications.

For peer review only

## INTRODUCTION

### Rationale

A thoracic aortic aneurysm (TAA) is an abnormal expansion of the aorta at any part of its course from the heart to the diaphragm. Aneurysms manifest with acute symptoms or they may be chronic. If untreated, TAA can continue to expand and the annual risk of aneurysm rupture increases from 2% in aneurysms smaller than 5cm in diameter to 7% in aneurysms over 6cm in diameter[1]. The majority of aneurysms (and therefore studies) centre on the ascending or abdominal segments of the aorta, arch and descending aneurysms are relatively under-studied. Therefore, this review focused on chronic aneurysms affecting the arch or descending thoracic aorta.

There are currently two main methods of repair for arch/descending TAA: Open Surgical Repair (OSR) and Endovascular Stent Grafting (ESG). OSR involves replacing the aneurysmal segment of aorta with a surgical graft during open surgery using heart-lung bypass. As the arch and descending aorta supply blood to the brain and spinal cord, OSR is high risk, with reported mortality and paraplegia rates of approximately 5% and 10% [2] respectively. In ESG a stent is placed across the aneurysm, providing a new conduit through which the blood flows. ESG is most often selected for patients who are considered at high risk of death or permanent disability from OSR. However, some patients may not have a suitable anatomy/morphology for ESG procedures [2]. Concerns have also been raised about long-term durability and safety of the implanted devices [3 4] due to complications such as stent migration or endoleaks, with the incidence of late endoleaks (leaks around or through the stent graft that occur more than one year after insertion) reported as being between 2.5% and 27.8% [5]. The need for re-intervention due to technical failures of the stent following ESG is also high (23% at 5 years) [6 7]. With each re-intervention there is an added risk of complication either due to the increased complexity of the procedure or deteriorating health of the patient [2].

One previous systematic review comparing only elective OSR and ESG for descending and arch TAAs has been reported [8]. This systematic review [8] was restricted to data from randomized controlled



For peer review only - http://bmjopen.bmj.com/site/about/guidelines.xhtml

trials in which patients were randomly assigned to ESG and OSR, but no eligible trials were identified when searching relevant data based up to Jan 2016. There are other systematic reviews [9 10] that have included non-randomized studies; however, these reviews also included emergency cases and other thoracic aortic pathologies (e.g. dissection or rupture). As such, they may not represent a true comparison of elective ESG and OSR procedures for descending thoracic aortic aneurysms. Given the lack of RCT evidence, this systematic review includes non-randomized comparative studies of the relative effectiveness of OSR or ESG.

The decision to operate requires a favourable balance between the risks and benefits of that intervention compared to those attached to the expected outcomes of the disease. This balance is in turn influenced by a number of factors.  Aneurysm size correlates to the risk of death (if left untreated) and so patients are usually referred once the aneurysm crosses a chosen threshold. At that point, patient fitness (*and life expectancy*), the reality or possibility of an intrinsic weakness affecting the whole aorta, the technical feasibility of the proposed intervention and patient wishes, come into play. This systematic review takes a step back from these issues and examines only the outcomes for patients in whom a decision to intervene has been made. This review is an essential starting point for the analysis of effective treatments for thoracic aortic aneurysms.

**Objectives**

The aim of this systematic review was to assess the effectiveness of endovascular stent grafting (ESG) compared to open surgical repair (OSR) as a treatment for chronic arch or descending TAA.



For peer review only - http://bmjopen.bmj.com/site/about/guidelines.xhtml

## METHODS

### Protocol and registration

The review protocol was registered in the PROSPERO database (CRD42017054565)[11].

### PUBLIC PATIENT INVOLVEMENT

There was no patient and public involvement in the whole process of conducting this review.

### Eligibility criteria

### Study designs

All identified studies that directly compared ESG and OSR, including RCTs, quasi-randomized and non-randomized controlled trials, comparative cohort studies and case-control studies matched on main outcomes were sought [12].

Non-randomized studies that controlled for selection bias were included – irrespective of study size. Acceptable methods for addressing selection bias included matching patients at baseline for important covariates, matching patients using propensity scores or adjustment for important covariates during analysis. Non-randomized studies that did not use these methods but included larger sample sizes (more than 250 patients) were also included as they have the potential to reduce sampling variation relative to smaller unmatched studies[13].

Non-English language papers were noted and, where possible, data were extracted from the abstract. No translation of full text articles was possible.

### Study Participants

Study participants had to receive elective treatments for arch and/ or descending thoracic aortic aneurysms. Where emergency interventions or other thoracic aortic conditions (such as intramural haematoma, rupture, and dissection) were reported, studies were excluded, unless results for patients receiving elective treatment for arch/descending TAA were reported separately. Studies



For peer review only - http://bmjopen.bmj.com/site/about/guidelines.xhtml

that reported secondary (re-intervention) procedures for arch/descending TAAs were included only if the original, primary procedure for each participant could be identified.

**Study interventions**

Open Surgical Repair was defined as replacement of the aneurysmal aorta with a prosthetic conduit via a sternotomy or thoracotomy with circulatory support. Endovascular stent grafting was defined as transluminal introduction of a stent-graft under X-ray guidance.

**Outcome measures**

This review assessed short-term and long-term clinical effectiveness. Short-term was defined as either within 30-days of the intervention and/or time until discharge following the intervention. Since most studies did not report long-term outcomes conditional on surviving the short-term period, long-term analyses covered the time from intervention until the end of follow-up. Primary outcomes were all-cause mortality and aneurysm-related mortality. Secondary outcomes were re-interventions and non-fatal complications. The latter were sub-classified as vascular, neurological, cardiac, respiratory and 'other' complications.

**Information sources**

MEDLINE, EMBASE, CENTRAL, WHO International Clinical Trials Routine data collection, Current Controlled Trials, Clinical Trials, and the UK National Institute for Health Research portfolio were searched. The search strategy (Appendix 1) was adapted as appropriate for each database (for example to allow for variations in controlled vocabulary terms and syntax [14]). Electronic searches identified publications between 1994, date of first reported case of ESG for descending and arch TAA[15], and March 2020.

Websites of regulatory bodies and Health Technology Assessment agencies were screened for unpublished relevant reports. A citation search of all included studies was conducted via Web of



For peer review only - http://bmjopen.bmj.com/site/about/guidelines.xhtml

For peer review only

Science. Studies from systematic reviews that satisfied the inclusion criteria of this review were screened for eligibility.

**Study selection**

All titles and abstracts were screened by one reviewer (AM). A second investigator (JG) reviewed 10% of all titles and abstracts classed as eligible or not eligible and all titles and abstracts classified as unclear by the first reviewer. The full text of selected studies was assessed by one investigator (AM) and classified as relevant, not relevant or unclear according to the inclusion criteria. A second investigator (JG) reviewed 10% of all papers, as well as all studies deemed unclear. Disagreements at either stage were resolved by discussion and where necessary a third reviewer (PM) arbitrated. Where required, a clinical expert (PS) was asked to screen papers.

**Data collection process**

A data extraction form was developed based on The Cochrane Collaboration data collection form [16]. Data were extracted by one reviewer (AM) and checked by a second reviewer (JG) for accuracy. Disagreements were resolved by discussion or arbitration by a third reviewer (PM).

**Data items**

Reported short-term and longer-term all-cause mortality, as well as short-term and longer-term aneurysm-related mortality were collected. All reported complications were recorded and grouped according to expert clinical guidance as described above as neurological, cardiac, respiratory or ‘other’ complications, with endoleaks following ESG recorded separately.

**Risk of bias in individual studies**

Risk of bias tools used were specific to the study design. As described later only non-randomized study designs were identified. Therefore, the ROBINS-I tool [17] was used. The ROBINS-I tool classifies the risk of bias in seven domains (*confounding; selection of participants; classification of*



For peer review only - http://bmjopen.bmj.com/site/about/guidelines.xhtml

*interventions; deviations from intended interventions; missing data; measurement of outcomes; selection of the reported results*) and overall as either critical, severe, moderate, low, or unclear.

**Synthesis of results**

Point estimates and precision of the incidence of short and long-term events and unadjusted and adjusted comparisons (odds ratios (OR) hazard ratios (HR)) were recorded. Meta-analysis of short-term event-comparisons was based on the OR with 95% confidence interval (CI) and conducted using RevMan [18]. An overall treatment effect was calculated for the log (OR) of each outcome using a (normal) random effects model. Where comparisons could not be made, data were tabulated and reported narratively.

Heterogeneity between studies was assessed according to Cochrane guidelines, on clinical criteria, by visual inspection of plots of the data, from the chi-square test for heterogeneity, and the I-squared statistic. An $I^2$ statistic of 25-50% indicated low heterogeneity; 50-75% indicated moderate heterogeneity, and over 75% indicating significant heterogeneity.

**RESULTS**

**Study selection**

The search identified 5163 unique studies. Of these, 5078 studies were not eligible. Ineligibility was primarily because of irrelevant anatomy (ascending, abdominal, or peripheral aneurysms), irrelevant pathology (acute dissection or other acute aortic syndromes), or because the study pooled data from a mixture of anatomical and pathological situations such that data on chronic arch and DTA aneurysms could not be extracted with confidence. Thus 85 papers underwent full text screening. 75 papers were rejected at this stage leaving 10 papers to be included in the full review. Six papers reported data from the Gore TAG Trial [19-24], leaving five unique comparative studies that fulfilled the inclusion criteria. Further details of the selection process are shown in the PRISMA diagram (Figure 1).



For peer review only - http://bmjopen.bmj.com/site/about/guidelines.xhtml

One study, Goodney et al 2011[25], reported both an unadjusted analysis for the unadjusted cohort and an analysis of a subgroup of propensity score matched patients. Data from the unadjusted cohort were used in all meta-analyses except the adjusted mortality meta-analysis, which used the propensity matched data. Brief summaries of the identified studies are given in Table 1, with detail provided in Appendix 2. Overall, the five studies included data on 3955 ESG and 21,197 OSR patients and all studies were conducted in Europe or the USA. Two studies had non-overlapping or only partially overlapping recruitment periods between the ESG and OSR intervention groups (Gore Tag[19-24] and Piffaretti et al 2007[26]). There were clear differences in patient characteristics between the two treatment groups. ESG patients were consistently older, with average age differences ranging from 2 to 9 years in all but one analysis (Goodney et al 2011[25] the propensity matched cohort). Similarly, in most studies the proportion of men was greater in the ESGgroup.

For peer review only

*Table 1: Study characteristics*

| Study | **Von Allmen et al 2014** | **Goodney et al 2011 (2 analyses*)** | **Gore TAG** | **Hughes et al 2014** | **Piffaretti et al 2007** |
|---|---|---|---|---|---|
| **METHODS** | | | | | |
| **Study Design** | Comparative cohort | Comparative cohort | Comparative cohort | Comparative cohort | Comparative cohort |
| Method of matching | None | 1. None 2. propensity matched | ESG patients judged eligible for OSR | None | OSR patients judged eligible for ESG |
| **PARTICIPANTS** | | | | | |
| **Country** | UK | USA | USA | USA | Italy |
| **Setting** | Multi-centre – Hospital episode statistics | Multi-centre – Medicare data | Multi-centre | Multi-centre | Single-centre |
| **Time frame** | 2006 to 2011 | 1. 1998-2007 2. 2003-2007 | ESG 1999-2001, OSR pre 1999 (n=44) & 1999-2001 (n=50) | 1998-2007 | ESG 2000-2007, OSR 1996-2000 |
| **Recruitment** | Total 618 ESG 354 OSR 264 OSR | 1. Total 13,998 (ESG 2433, OSR 11,565) | Total 234 ESG 140 OSR 94 | Total 8967 ESG 712 OSR 8255 | Total 28 ESG 17 OSR 11 |



For peer review only - http://bmjopen.bmj.com/site/about/guidelines.xhtml

| | | | | | |
|---|---|---|---|---|---|
| | | 2. Total 1100 (ESG 550, OSR 550) | | | |
| **Age summary** | median (IQR) ESG 73(66,78), OSR 71 (63-76) | mean (95% C.I.) 1. ESG 75.9 (75.6, 76.1), OSR 73.8 (73.7, 73.9) 2. ESG 71.1 (70.8, 71.4), OSR 70.7 (70.7, 71.1) | mean(SD) ESG 75.9 (10.4), OSR 68.2 (10.2) | median (IQR) ESG 72 (63-78), OSR 63 (52, 72) | mean (SD) ESG 66 (10), OSR 61 (13) |
| **Proportion male** | ESG 232 (65.5%), OSR 137 (51.9%) | % (95% C.I.): 1. ESG 58.7 (56.7, 60.7), OSR 55.4 (54.8, 56.3) 2. ESG 64.0 (59.9, 68.0), OSR 68.1% (64.2, 72.0) | ESG 90(57%), OSR 48 (51%) | 5415 (61.3%) ESG, 437 (65.6%) OSR | ESG 14 (82%), OSR 8 (73%) |
| **OUTCOMES** | | | | | |
| **Short-term** | Death from any cause | Death from any cause | Death from any cause | Death from any cause | Death from any cause |



For peer review only - http://bmjopen.bmj.com/site/about/guidelines.xhtml

For peer review only

| | | | | | |
|---|---|---|---|---|---|
| | Re-intervention All-cause mortality (unadjusted | | Complications | Complications | Complications |
| **Long-term** | Time to death from any cause Time to aortic-related death Time to aortic-related re-intervention | Time to death from any cause | Time to death from any cause Time to aneurysm-related death aneurysm-related mortality Time to re-intervention Time to major adverse event | Not reported | Time to death from any cause |
| * 1. Unmatched cohort, 2. Propensity matched cohort | | | | | |

**Risk of bias within studies**

The propensity-matched analysis of Goodney et al (2011) [25] was judged to be of overall moderate risk of bias and four studies were at severe risk of bias due to the potential for confounding (Appendix 3).



For peer review only - http://bmjopen.bmj.com/site/about/guidelines.xhtml

Two large cohorts derived from UK and USA administrative databases (Von Allmen et al 2014[27] and Hughes et al 2014[28]) did not attempt to address selection bias at the design stage, but did adjust for some confounders in the analysis. A third study (Goodney et al 2011[25]), using USA administrative data, included both unadjusted population and analysis of a subset of the data defined by propensity score matching. Propensity score methods rely on all important confounders being available and included[29]. As this study did not include all confounders considered important, such as aneurysm size, this study was judged to have moderate risk of bias due to confounding.

One study (Gore TAG)[19-24] included only ESG patients who would also have been eligible for OSR and the another study (Piffaretti et al 2007)[26] included only OSR patients who would be suitable for ESG, both according to clinician judgement. Since suitability was decided retrospectively, such matching may not be robust.

A further potential bias with each study was selective reporting. Although the measurements and analyses of the outcomes were clearly defined within each study, no evidence of a pre-study analysis plan was identified.

**Synthesis of results**

**Short-term death from any cause (30-day or in hospital)**

*Unadjusted Mortality:*

All five studies[19-28] reported unadjusted 30 day or in-hospital mortality (Figure 2). Overall, the odds of short-term mortality were lower in the ESG group than the OSR group (OR: 0.75; 95 %CI. (0.55-1.03)). Moderate heterogeneity was present in the overall analysis ($I^2$= 40%), mainly due to differences between the two smaller studies[19-24 26] and the three larger studies [25 27 28], with a large $I^2$ for subgroup differences between the larger studies subgroup and the smaller studies subgroup of 84.5%.



For peer review only - http://bmjopen.bmj.com/site/about/guidelines.xhtml

For the three larger studies[25 27 28] (3798 ESG and 21,092 OSR patients), the treatment effect for death was smaller (OR: 0.84 95%CI (0.72, 0.98)) than that for the two smaller studies[19-24 26] (157 ESG and 105 OSR patients, combined OR: 0.18 95%CI (0.06, 0.59)). There was no evidence of heterogeneity in results between these subgroups ($I^2$=0).

*Sensitivity analysis*

Hughes et al 2014[28] was the only study to include aortic arch aneurysms. Arch aneurysms tend to be more complex procedures and are mostly conducted using OSR. A sensitivity analysis excluding this study was therefore conducted (Figure 3).

While the point effect estimates all favoured ESG, the overall pooled odds ratio (OR: 0.73; 95 %CI. (0.45-1.18)) did not reach statistical significance (p=0.2). Moderate heterogeneity was present in the overall analysis ($I^2$= 42%), mainly due to differences between the two smaller studies and the two larger studies, with a large $I^2$ for subgroup differences between the larger studies subgroup and the smaller studies subgroup of 65.1%.

For the two larger studies[25 27] (3086 ESG and 12837 OSR patients), the treatment effect for death was smaller (OR: 0.85 95%CI (0.72, 1.00)) than that for the two smaller studies[19-24 26] (157 ESG and 105 OSR patients, combined OR: 0.20 95%CI (0.04, 1.06)). There was no evidence of heterogeneity in results within the large subgroup ($I^2$= 0) and evidence of low heterogeneity withing the small studies subgroup ($I^2$=26%).

*Adjusted Mortality:*

The three larger studies [25 27 28] included adjusted analysis of short-term mortality (Figure 4). Goodney et al 2011[25] used stratified propensity matching. Hughes et al 2014 [28] adjusted for preoperative comorbidities, independent predictors of mortality and post-operative complications and the Von Allmen 2014[27] study adjusted for age and sex. The OR for ESG, relative to OSR, for these three



For peer review only - http://bmjopen.bmj.com/site/about/guidelines.xhtml

studies combined was 0.71, 95%CI (0.51, 0.98). No evidence of heterogeneity was identified in this meta-analysis ($I^2$=0%)

**All-cause mortality (following hospital discharge or after 30 days)**

Meta-analysis of the longer-term all-cause mortality was not possible as follow-up varied between the four studies that reported this outcome (Gore Tag[19-24], Goodney et al 2011[25], Piffaretti et al 2007[26] and Von Allmen 2014[27]) . The results in the larger studies (Goodney et al 2011[25], Von Allmen 2014[27]) differed from those in the smaller studies (Gore Tag[19-24] and Piffaretti et al 2007[26]). We note that the analyses included the short-term deaths as well as those occurring beyond hospital discharge or after 30 days.

Von Allmen et al 2014[27] reported time to death from any cause up to five years, throughout which OSR had higher overall survival. Adjusting for age and sex, ESG had higher odds of death at one year (OR: 1.10 95%CI (0.70, 1.73); p=0.667). Additionally, the hazard ratio for ESG relative to OSR up to five years, adjusting for age and sex, was HR=1.45 (95%CI [1.08, 1.94], p=0.013).

Goodney et al 2011[25] also reported time to death from any cause up to five years. Adjusting for age, sex, race, era of procedure and Charlson comorbidity score, five-year survival for OSR in the unmatched cohort was higher than for ESG (89% versus 79%, log-rank p<0.0001). In the propensity-matched cohort, five-year life-table analysis reported higher survival in the OSR group (81% 95%CI (77%-85%)) than for the ESG group (73% 95%CI (68%, 76%)) (log-rank p=0.007).

In the Gore TAG study[19-24], Time to death from any cause over five years did not differ between the ESG and OSR groups, with survival probability at five years in both groups of 63% (log rank P value= 0.625). Piffaretti et al 2007[26] reported survival overall in the ESG group up to five years and the OSR group up to 13 years. For the five years in which survival is reported in both groups, ESG had a higher



For peer review only - http://bmjopen.bmj.com/site/about/guidelines.xhtml

survival compared to OSR although this was based on only 4 deaths, and the follow-up periods differed substantially (log rank p>0.05).

**Aneurysm/Aortic-related mortality**

Meta-analysis of Aneurysm/aortic-related mortality could not be conducted. No studies reported aneurysm-related mortality during hospitalization or up to 30 days separately to the long-term. Von Allmen et al 2014[27] defined aortic-related mortality as any aortic-related death occurring within five years of the index operation according to ICD-10 codes referring to aorta, or cause of deaths closely related to complications of surgery (e.g. paraplegia). Time to aortic-related death did not differ between OSR group than the ESG group during the five-year follow-up (log-rank p=0.270). This study[27] also reported adjusted (for age and sex) five year HR of 1.27 (0.81 to 1.98; P =0.298) for ESG patients relative to OSR patients.

Gore TAG[19-24] defined aneurysm related mortality as deaths that occurred in hospital or within 30 days of initial procedure or any reintervention or a death due to the aneurysm or the treatment device and reported up to five years, with longer time to aneurysm-related death in the ESG group over this period (log-rank p= 0.012).

**Short-term re-interventions**

One study reported aortic-related re-interventions in 21 (5.9%) ESG patients and 4 (1.5%) OSR patients, an adjusted OR of 2.97 (95%CI [1.09, 8.10], p=0.033) for ESG versus OSR (Von Allmen et al 2014[27]).

**Long-term re-interventions**

Two studies reported this outcome (Von Allmen et al 2014[27] and Gore TAG [19-24]). Von Allmen et al 2014[27] reported time to aortic-related re-interventions up to five years. Time to re-intervention was



For peer review only - http://bmjopen.bmj.com/site/about/guidelines.xhtml

longer in the OSR group compared to the ESG group (log-rank p=0.007), with adjusted (for age and sex) hazard ratio at five years of 1·70 (95%CI [1·11 to 2·60], p =0·014). In Gore TAG[19-24], time to re-intervention over five years was longer in the ESG group (log-rank P=0.011). We note that these analyses included short-term re-interventions.

**Complications**

Table 2 summarises the short-term complications reported in all five studies[19 25-28]. Most studies reported a small number of neurological, pulmonary, cardiac and other complications. However, one larger study (Hughes et al 2014)[28] reported lower odds for ESG patients for neurological complications, OR of 0.48 (95%CI (0.26, 0.86), p=0.015), pulmonary complications, OR: 0.38 (95%CI (0.21, 0.67), p=0.001) and cardiac complications, OR: 0.24 (95%CI (0.15, 0.37), p<0.001). The Gore TAG study[19-24] reported endoleak rates for ESG patients of 8.5%.

*Table 2: Short-term complications*

| Study | Gore TAG | | Piffaretti et al 2007 | | Hughes et al 2011 | |
|---|---|---|---|---|---|---|
| **Arm (participants)** | **ESG (n=140)** | **OSR (n=94)** | **ESG (n=17)** | **OSR (n=11)** | **ESG (n=712)** | **OSR (n=8255)** |
| **NEUROLOGICAL** | | | | | | |
| **Paraplegia** | NR | NR | NR | NR | NR | NR |
| **Paraplegia/ Paraperisis** | 4 (3%) | 13 (14%) | NR | NR | NR | NR |
| **Cerebral Vascular Accident** | 5 (4%) | 5 (4%) | 2 (9%) | 1 (12%) | NR | NR |
| **Neurological-unspecified type** | NR | NR | NR | NR | 20 (2.8%) | 273 (3.3%) |
| **RESPIRATORY** | | | | | | |



For peer review only - http://bmjopen.bmj.com/site/about/guidelines.xhtml

| | | | | | | |
|---|---|---|---|---|---|---|
| **Respiratory failure** | 5 (4%) | 19 (20%) | NR | NR | NR | NR |
| **Pneumonia** | NR | NR | 2 (12%) | 3 (27%) | NR | NR |
| **Pulmonary-unspecified type** | NR | NR | NR | NR | 17 (2.4%) | 462 (5.6%) |
| **CARDIAC** | | | | | | |
| **Myocardial Infarction** | 0 (0%) | 1(1%) | 0 (0%) | 1 (9%) | NR | NR |
| **Cardiac-unspecified type** | NR | NR | NR | NR | 28 (2.9%) | 1252 (15.2%) |
| **ENDOLEAKS** | | | | | | |
| **Endoleaks** | 12 (8.5%) | NR | NR | NR | NR | NR |
| **OTHER** | | | | | | |
| **Peripheral vascular disease** | 20 (14%) | 4 (4%) | NR | NR | NR | NR |
| **Renal failure** | 2 (1%) | 12 (13%) | NR | NR | NR | NR |
| **Wound infection/dehiscence** | 5 (4%) | 10 (11%) | NR | NR | NR | NR |
| **GI complication** | 3 (2%) | 6 (6%) | NR | NR | NR | NR |
| **Post-implant syndrome** | NR | NR | 4(18%) | 0(0%) | NR | NR |
| *Note: Goodney et al 2011 and Von Allmen et al 2014 did not report short-term complications* | | | | | | |

**DISCUSSION**

This is the first systematic review that reports results from non-randomized comparative studies regarding the effectiveness of elective ESG and OSR for arch and or descending TAA. Previous reviews have either included single arm studies only[30] or were comparative reviews that included non-elective procedures[9 31], or mixed pathologies such as dissections[10 32]. The results of these



For peer review only - http://bmjopen.bmj.com/site/about/guidelines.xhtml

reviews may not be applicable to patients undergoing elective procedures for descending or arch TAA pathology.

In this review only five comparative cohort studies were identified. The lack of RCTs comparing ESG and OSR for elective arch/descending thoracic aortic aneurysms may reflect the lack of equipoise from practitioners and patients. Well-designed non-randomized trials can demonstrate a high degree of concordance with the results of well-designed RCTs, provided that all confounders are adequately accommodated in the design and/or analysis[33]. However, the studies identified in this review had moderate to serious risk of bias, mainly due to potential for confounding.

Although, complications in the short-term also appear to favour ESG, evidence regarding short-term complications is either lacking or inconsistently reported. All five studies[19 25-28] suggested that the less invasive nature of ESG confers lower short-term mortality and fewer short-term non-fatal complications than OSR. The two small unadjusted studies[19-24 26] had a much larger effect than larger studies[25 27 28]. These larger studies used more robust methods for addressing confounding; this may indicate the presence of publication bias where only those smaller studies with larger effect sizes have been published.

Longer-term results are difficult to interpret since most data were not presented in a way to separate early risk from longer term survival. The two smaller studies[19-24 26] reported continuing survival benefit for ESG patients in the long-term but are limited by small numbers, different recruitment periods, and failure to exclude early risk. The survival advantage associated with ESG in the Gore TAG[19-24] study disappears by year five, which suggests that the hazards are lower for OSR patients that survive the initial procedure. Conversely, the two larger studies (Von Allmen et al 2014[27] and Goodney et al 2011[25]) report that the short-term survival advantage of ESG were reversed by twelve months, and that the OSR group had an increasing survival advantage thereafter. Similarly, time to re-intervention favoured ESG in the smaller Gore TAG[19-24] study, whilst time to aortic-related re-interventions favoured OSR in the larger study Von Allmen et al 2014[27].



For peer review only - http://bmjopen.bmj.com/site/about/guidelines.xhtml

The evidence regarding mortality is inconsistent across the studies with more recent, larger studies[25 27 28] reporting a more modest survival benefit for ESG in the short-term and a survival benefit for OSR in the long-term. The two smaller studies [19-24 26]suggest a large reduction in the risk of mortality and short term complications in the short run and a survival benefit in the long-run for ESG.

Inconsistency of results across studies may be due to heterogeneity of the study populations, both within and across studies. Differences in recruitment periods may reflect changes in technology and practice in both groups. The larger studies [25 27 28] were based on patients recruited up to twenty years later than the smaller studies. Outcomes may have improved differentially with OSR patients receiving proportionally better outcomes due to general improvements in surgical practice and patient selection over time[34]. A potential reason for the reported long-term survival benefit of OSR in more recent, larger studies may be differences in patient risk in each intervention group. Open surgical repair is advocated for younger, lower-risk patients, and EVAR is preferred for older, higher-risk patients[35]. This selection bias may not have been adequately accommodated in adjusted analyses.

The three large studies[25 27 28] were based on administrative health records with case ascertainment that depends on accurate coding, which can vary across and within providers[36]. Furthermore, one of the small studies, Piffaretti et al 2007[26], recruited patients from one centre which may have compromised external validity as they may for example, depend on the expertise within a centre and the nature of the patients that centre treats[37 38]. Additionally, the Gore TAG study[19-24] utilised a more restricted inclusion criteria for ESG patients compared to those who received OSR, potentially introducing heterogeneity between the two groups.

The headline conclusion of this review is that whilst there is a degree of variability, OSR is better for long-term survival and avoidance of re-intervention, and that ESG is better for early outcomes. This conclusion is based on non-randomized comparisons in which the influence of important confounders for choice of intervention (ESG or OSR) has not been adjusted for. Deeper exploration is



For peer review only - http://bmjopen.bmj.com/site/about/guidelines.xhtml

required, but randomized data may be difficult to acquire as there are well-established guidelines about which patients are favoured for OSR and which for ESG. Thus, to compare ESG and OSR it is first necessary to establish an understanding of the reasons driving clinical management, for example by a structured consensus analysis such as a Delphi study. Thereafter, unless there is clinical equipoise about which patients are best served by OSR or ESG, the best quality evidence to establish the relative merits of ESG and OSR for arch/ DTA aneurysms is likely to be a systematic, prospective, observational, comparative study, using individual patient data.

**Limitations**

The findings in this review are limited by the number and potential for confounding of the five studies[19-28] identified, so that definitive conclusions are not possible. The adjustment for confounders is likely to be only partial and some bias in the estimates of differences in mortality and complications may persist, despite attempts to reduce bias in the larger studies identified. Statistical analysis of non-fatal complications was not possible due to limited and inconsistent reporting.

## CONCLUSIONS

This systematic review is the first to consider evidence from non-randomized studies that directly compare ESG and OSR for the treatment of elective arch/descending thoracic aortic aneurysms. Other reviews conducted in this area have been complicated by the inclusion of treatments for emergency cases or other thoracic aortic pathologies (e.g. thoracic aortic ulcer, or dissection).

There is limited and increasingly dated evidence addressing the comparison of ESG and OSR in the management of elective arch and or descending thoracic aortic aneurysms. Further, high quality, evidence is required. Although large RCTs may not be possible in this relatively rare condition, future studies should use strong methodological study design in order to control for potential confounders and reduce potential biases. Additionally, given the current conflicting evidence, it is important that comparisons of OSR and ESG are conducted in both the short-term and the long-term.



For peer review only - http://bmjopen.bmj.com/site/about/guidelines.xhtml

**FUNDING**

"This report is independent research funded by the National Institute for Health Research **(Health Technology Assessment, 11/147/03 - Effective Treatments for Thoracic Aortic Aneurysms (ETT AA study): A prospective cohort study).** The views expressed in this publication are those of the author(s) and not necessarily those of the NHS, the National Institute for Health Research or the Department of Health."

**CONFLICT OF INTEREST**

Andrew McCarthy, Joanne Gray, Linda Sharples, Priya. Sastry, Peter McMeekin, Andrew Cook have nothing to disclose. Luke Vale reports grants from NIHR Health Technology Assessment Programme, during the conduct of the study; and being a member of the NIHR Health Technology Assessment Programme Clinical Trials and Evaluation Panel from 2015-2018. Carol Freeman reports grants from DoH NIHR HTA Programme, during the conduct of the study. Pedro Catarino reports grants from NIHR, outside the submitted work. Stephen. Large reports grants from NIHR, during the conduct of the study.

**ACKNOWLEDGMENTS**

The authors would like to thank our Funders and the ETTAA Working Group for their support with this study.

**AUTHOR CONTRIBUTIONS**

Author contributions according to the CrediT (contributor roles taxonomy). Conceptualization- AM, JG, PS, LS, LV, AC, PM, SL; Data curation- AM ;Formal Analysis- AM, JG, PM; Funding acquisition- JG, AC, LV, CF, PS, LS, SL; Investigation - AM, JG, PM, LV, PS; Methodology - AM, JG, PM, LV, AC, CF, PS, LS, SL; Supervision - JG, PM, LV; Validation – AM, JG, PM, PS, LS, PC, SL; Writing – original draft - AM, JG, PM; Writing – review & editing - AM, JG, PM, AC, LV, CF, PS, LS, PC, SL.



For peer review only - http://bmjopen.bmj.com/site/about/guidelines.xhtml

**DATA SHARING AGREEMENT**

Data were extracted from the studies included in this Systematic Review and referenced below.

**REFERENCES**


1. Akin I, Kische S, Rehders TC, et al. Endovascular repair of thoracic aortic aneurysm. *Archives of medical science : AMS* 2010;6(5):646-52. doi: 10.5114/aoms.2010.17075 [published Online First: 2010/10/26]
2. Sastry P, Hughes V, Hayes P, et al. The ETTAA study protocol: a UK-wide observational study of 'Effective Treatments for Thoracic Aortic Aneurysm'. *BMJ Open* 2015;5(6) doi: 10.1136/bmjopen-2015-008147
3. Kim LG, Sweeting MJ, Epstein D, et al. Optimizing Surveillance and Re-intervention Strategy Following Elective Endovascular Repair of Abdominal Aortic Aneurysms. *Annals of Surgery* 2019;Publish Ahead of Print doi: 10.1097/sla.0000000000003625
4. Patel R, Sweeting MJ, Powell JT, et al. Endovascular versus open repair of abdominal aortic aneurysm in 15-years' follow-up of the UK endovascular aneurysm repair trial 1 (EVAR trial 1): a randomised controlled trial. *The Lancet* 2016;388(10058):2366-74. doi: 10.1016/S0140-6736(16)31135-7
5. Ricotta JJ. Endoleak management and postoperative surveillance following endovascular repair of thoracic aortic aneurysms. *Journal of Vascular Surgery* 2010;52(4, Supplement):91S-99S. doi: https://doi.org/10.1016/j.jvs.2010.06.149
6. Kalteis M, Haller F, Artmann A, et al. Experience and Outcomes After a Decade of Endovascular Abdominal Aortic Aneurysm Repair: A Retrospective Study From a Community-Based Single Center. *Annals of Vascular Surgery* 2012;26(3):330-37. doi: https://doi.org/10.1016/j.avsg.2011.06.012
7. Mertens J, Houthoofd S, Daenens K, et al. Long-term results after endovascular abdominal aortic aneurysm repair using the Cook Zenith endograft. *Journal of Vascular Surgery* 2011;54(1):48-57.e2. doi: https://doi.org/10.1016/j.jvs.2010.12.068
8. Abraha I, Romagnoli C, Montedori A, et al. Thoracic stent graft versus surgery for thoracic aneurysm. *Cochrane Database of Systematic Reviews* 2016(6) doi: 10.1002/14651858.CD006796.pub4
9. Health Quality O. Endovascular Repair of Descending Thoracic Aortic Aneurysm: An Evidence-Based Analysis. *Ontario Health Technology Assessment Series* 2005;5(18):1-59.
10. Walsh SR, Tang TY, Sadat U, et al. Endovascular stenting versus open surgery for thoracic aortic disease: Systematic review and meta-analysis of perioperative results. *Journal of Vascular Surgery* 2008;47(5):1094-98.e3. doi: https://doi.org/10.1016/j.jvs.2007.09.062
11. Gray J, McCarthy A, McMeekin P, et al. Effective Treatments for Thoracic Aortic Aneurysms (ETTAA study): protocol for a systematic review. *PROSPERO* 2016(CRD420170545565)
12. Reeves BC, Deeks JJ, Higgins JP, et al. Including Non-Randomized Studies. In: Higgins JP, Green S, eds. Cochrane Handbook for Systematic Reviews of Interventions2008.
13. Deeks J, Dinnes J, D'Amico R, et al. Evaluating non-randomised intervention studies. *Health Technology Assessment* 2003;7(27)
14. Texas Uo. Search Filters for Various Databases: PubMed 2016 [updated 3 October 2017. Available from: https://libguides.sph.uth.tmc.edu/search_filters accessed 14 July 2016.




For peer review only - http://bmjopen.bmj.com/site/about/guidelines.xhtml

15. Dake MD, Miller DC, Semba CP, et al. Transluminal Placement of Endovascular Stent-Grafts for the Treatment of Descending Thoracic Aortic Aneurysms. *New England Journal of Medicine* 1994;331(26):1729-34. doi: 10.1056/nejm199412293312601
16. Higgins JP, Green S. Guide to the Contents of a Cochrane Protocol and Review. Cochrane Handbook for Systematic Reviews of Interventions2008.
17. Sterne JA, Hernán MA, Reeves BC, et al. ROBINS-I: a tool for assessing risk of bias in non-randomised studies of interventions. *BMJ* 2016;355 doi: 10.1136/bmj.i4919
18. Review Manager (RevMan) [program]. Version 5.3 version. The Nordic Cochrane Centre: The Cochrane Collaboration, 2014.
19. Bavaria JE, Appoo JJ, Makaroun MS, et al. Endovascular stent grafting versus open surgical repair of descending thoracic aortic aneurysms in low-risk patients: a multicenter comparative trial. *Journal of Thoracic & Cardiovascular Surgery* 2007;133(2):369-77.
20. Cho JS, Haider SE, Makaroun MS. Endovascular therapy of thoracic aneurysms: Gore TAG trial results. *Seminars in Vascular Surgery* 2006;19(1):18-24.
21. Cho JS, Haider SE, Makaroun MS. US multicenter trials of endoprostheses for the endovascular treatment of descending thoracic aneurysms. *Journal of Vascular Surgery* 2006;43 Suppl A:12A-19A.
22. Go MR, Cho J-S, Makaroun MS. Mid-term Results of a Multicenter Study of Thoracic Endovascular Aneurysm Repair Versus Open Repair. *Perspectives in Vascular Surgery and Endovascular Therapy* 2007;19(2):124-30. doi: 10.1177/1531003507303430
23. Makaroun MS, Dillavou ED, Wheatley GH, et al. Five-year results of endovascular treatment with the Gore TAG device compared with open repair of thoracic aortic aneurysms. *Journal of Vascular Surgery* 2008;47(5):912-8.
24. Dillavou ED, Makaroun MS. Predictors of morbidity and mortality with endovascular and open thoracic aneurysm repair. *Journal of Vascular Surgery* 2008;48(5):1114-9; discussion 19-20.
25. Goodney PP, Travis L, Lucas FL, et al. Survival after open versus endovascular thoracic aortic aneurysm repair in an observational study of the Medicare population. *Circulation* 2011;124(24):2661-9.
26. Piffaretti G, Tozzi M, Lomazzi C, et al. Endovascular repair versus conventional surgery for descending thoracic aortic aneurysms. *Italian Journal of Vascular and Endovascular Surgery* 2007;14(4):279-85.
27. Von Allmen RS, Anjum A, Powell JT. Outcomes after endovascular or open repair for degenerative descending thoracic aortic aneurysm using linked hospital data. *British Journal of Surgery* 2014;101(10):1244-51.
28. Hughes K, Guerrier J, Obirieze A, et al. Open versus endovascular repair of thoracic aortic aneurysms: a Nationwide Inpatient Sample study. *Vascular & Endovascular Surgery* 2014;48(5-6):383-7.
29. Austin PC. An Introduction to Propensity Score Methods for Reducing the Effects of Confounding in Observational Studies. *Multivariate Behav Res* 2011;46(3):399-424. doi: 10.1080/00273171.2011.568786 [published Online First: 2011/06/08]
30. Eggebrecht H, Nienaber CA, Neuhäuser M, et al. Endovascular stent–graft placement in aortic dissection: a meta-analysis. *European Heart Journal* 2005;27(4):489-98. doi: 10.1093/eurheartj/ehi493
31. Alsawas M, Zaiem F, Larrea-Mantilla L, et al. Effectiveness of surgical interventions for thoracic aortic aneurysms: A systematic review and meta-analysis. *Journal of Vascular Surgery* 2017;66(4):1258-68.e8. doi: https://doi.org/10.1016/j.jvs.2017.05.082
32. Cheng D, Martin J, Shennib H, et al. Endovascular Aortic Repair Versus Open Surgical Repair for Descending Thoracic Aortic Disease: A Systematic Review and Meta-Analysis of Comparative Studies. *Journal of the American College of Cardiology* 2010;55(10):986-1001. doi: https://doi.org/10.1016/j.jacc.2009.11.047



For peer review only - http://bmjopen.bmj.com/site/about/guidelines.xhtml

33. Marko NF, Weil RJ. The Role of Observational Investigations in Comparative Effectiveness Research. *Value in Health* 2010;13(8):989-97. doi: https://doi.org/10.1111/j.1524-4733.2010.00786.x
34. Ludman Peter F, Moat N, de Belder Mark A, et al. Transcatheter Aortic Valve Implantation in the United Kingdom. *Circulation* 2015;131(13):1181-90. doi: 10.1161/CIRCULATIONAHA.114.013947
35. Katzen BT, Dake MD, MacLean AA, et al. Endovascular Repair of Abdominal and Thoracic Aortic Aneurysms. *Circulation* 2005;112(11):1663-75. doi: doi:10.1161/CIRCULATIONAHA.105.541284
36. Prasad A, Helder MR, Brown DA, et al. Understanding Differences in Administrative and Audited Patient Data in Cardiac Surgery: Comparison of the University HealthSystem Consortium and Society of Thoracic Surgeons Databases. *Journal of the American College of Surgeons* 2016;223(4):551-57.e4. doi: https://doi.org/10.1016/j.jamcollsurg.2016.06.393
37. Bellomo R, Warrillow SJ, Reade MC. Why we should be wary of single-center trials. *Critical Care Medicine* 2009;37(12):3114-19. doi: 10.1097/CCM.0b013e3181bc7bd5
38. Unverzagt S, Prondzinsky R, Peinemann F. Single-center trials tend to provide larger treatment effects than multicenter trials: a systematic review. *Journal of Clinical Epidemiology* 2013;66(11):1271-80. doi: 10.1016/j.jclinepi.2013.05.016

Figure 1: PRISMA Diagram

Figure 2: Forest plot of unadjusted short-term all-cause mortality

Figure 3: Forest plot of unadjusted short-term all-cause mortality. Sensitivity Analysis.

Figure 4: Forest plot of adjusted short-term all-cause mortality

For peer review only



For peer review only - http://bmjopen.bmj.com/site/about/guidelines.xhtml

*Figure 1: PRISMA Diagram*

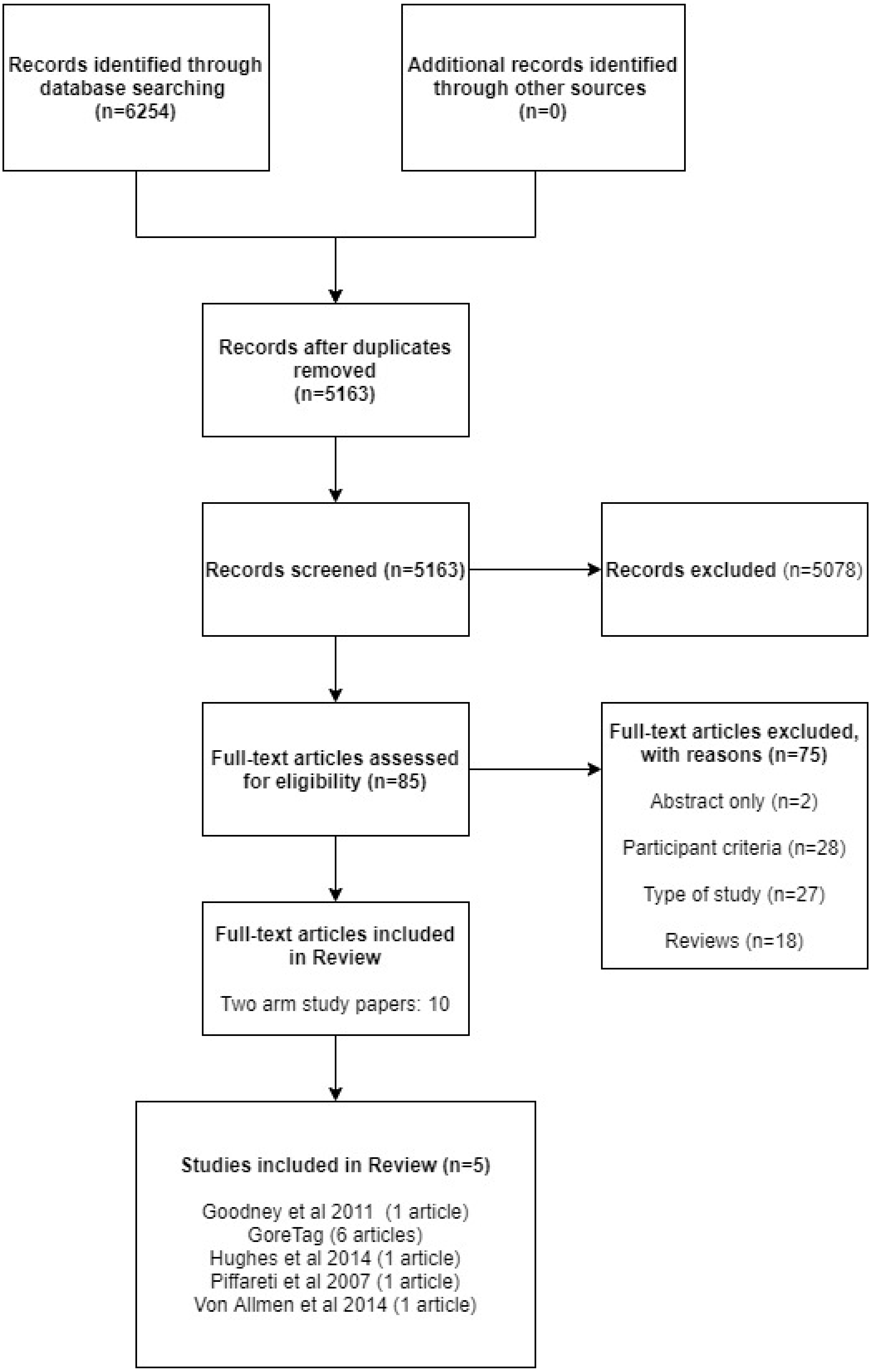

*Figure 2: Forest plot of unadjusted short-term all-cause mortality*

| Study or Subgroup | ESG Events | ESG Total | OSR Events | OSR Total | Weight | Odds Ratio M-H, Random, 95% CI |
|---|---|---|---|---|---|---|
| **1.1.1 Subgroup 1** | | | | | | |
| Goodney 2011 | 167 | 2732 | 893 | 12573 | 47.7% | 0.85 [0.72, 1.01] |
| Hughes 2014 | 26 | 712 | 380 | 8255 | 28.6% | 0.79 [0.52, 1.18] |
| Von Allmen 2014 | 23 | 354 | 20 | 264 | 17.2% | 0.85 [0.46, 1.58] |
| **Subtotal (95% CI)** | | **3798** | | **21092** | **93.4%** | **0.84 [0.72, 0.98]** |
| Total events | 216 | | 1293 | | | |
| Heterogeneity: Tau² = 0.00; Chi² = 0.13, df = 2 (P = 0.94); I² = 0% | | | | | | |
| Test for overall effect: Z = 2.22 (P = 0.03) | | | | | | |
| **1.1.2 Subgroup 2** | | | | | | |
| Gore TAG | 3 | 140 | 11 | 94 | 5.1% | 0.17 [0.04, 0.61] |
| Piffaretti 2007 | 1 | 17 | 2 | 11 | 1.5% | 0.28 [0.02, 3.55] |
| **Subtotal (95% CI)** | | **157** | | **105** | **6.6%** | **0.18 [0.06, 0.59]** |
| Total events | 4 | | 13 | | | |
| Heterogeneity: Tau² = 0.00; Chi² = 0.13, df = 1 (P = 0.71); I² = 0% | | | | | | |
| Test for overall effect: Z = 2.85 (P = 0.004) | | | | | | |
| **Total (95% CI)** | | **3955** | | **21197** | **100.0%** | **0.75 [0.55, 1.03]** |
| Total events | 220 | | 1306 | | | |
| Heterogeneity: Tau² = 0.04; Chi² = 6.72, df = 4 (P = 0.15); I² = 40% | | | | | | |
| Test for overall effect: Z = 1.80 (P = 0.07) | | | | | | |
| Test for subgroup differences: Chi² = 6.45, df = 1 (P = 0.01), I² = 84.5% | | | | | | |

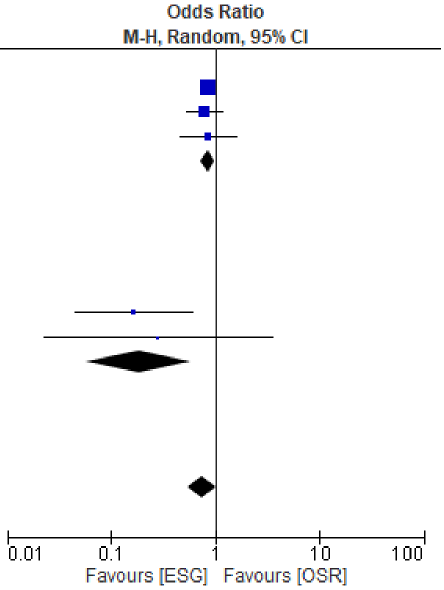


For peer review only

*Figure 3: Forest plot of unadjusted short-term all-cause mortality. Sensitivity Analysis.*

| Study or Subgroup | ESG Events | ESG Total | OSR Events | OSR Total | Weight | Odds Ratio M-H, Random, 95% CI |
|---|---|---|---|---|---|---|
| **1.1.1 large studies** | | | | | | |
| Von Allmen 2014 | 23 | 354 | 20 | 264 | 30.9% | 0.85 [0.46, 1.58] |
| Goodney 2011 | 167 | 2732 | 893 | 12573 | 58.4% | 0.85 [0.72, 1.01] |
| **Subtotal (95% CI)** | | **3086** | | **12837** | **89.3%** | **0.85 [0.72, 1.00]** |
| Total events | 190 | | 913 | | | |
| Heterogeneity: Tau² = 0.00; Chi² = 0.00, df = 1 (P = 0.99); I² = 0% | | | | | | |
| Test for overall effect: Z = 1.92 (P = 0.06) | | | | | | |
| **1.1.2 Small studies** | | | | | | |
| Piffaretti 2007 | 1 | 17 | 11 | 94 | 4.8% | 0.47 [0.06, 3.91] |
| Gore TAG | 3 | 140 | 2 | 11 | 5.8% | 0.10 [0.01, 0.67] |
| **Subtotal (95% CI)** | | **157** | | **105** | **10.7%** | **0.20 [0.04, 1.06]** |
| Total events | 4 | | 13 | | | |
| Heterogeneity: Tau² = 0.37; Chi² = 1.35, df = 1 (P = 0.25); I² = 26% | | | | | | |
| Test for overall effect: Z = 1.89 (P = 0.06) | | | | | | |
| **Total (95% CI)** | | **3243** | | **12942** | **100.0%** | **0.73 [0.45, 1.18]** |
| Total events | 194 | | 926 | | | |
| Heterogeneity: Tau² = 0.10; Chi² = 5.14, df = 3 (P = 0.16); I² = 42% | | | | | | |
| Test for overall effect: Z = 1.28 (P = 0.20) | | | | | | |
| Test for subgroup differences: Chi² = 2.87, df = 1 (P = 0.09), I² = 65.1% | | | | | | |

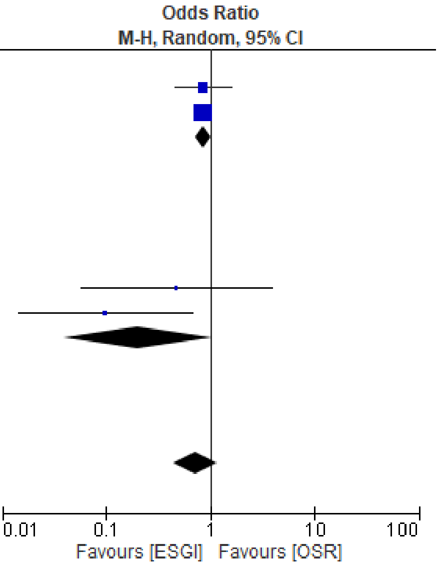


peer review only

*Figure 4: Forest plot of adjusted short-term all-cause mortality*

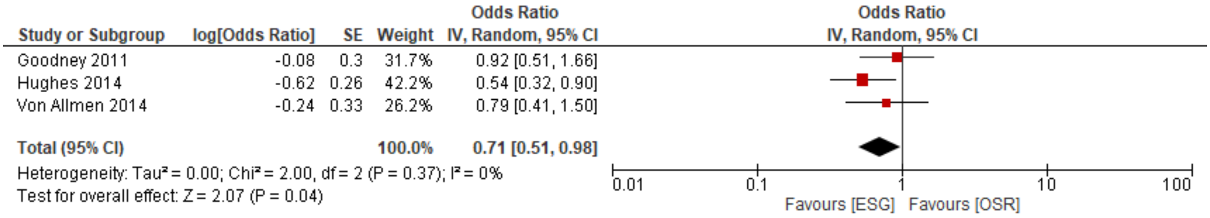


peer review only

**Appendix 1: Search Strategy**

**Medline/Central/HTA**

1. (TEVAR or EVAR).ti,ab.
2. (endovascular adj1 (stent* or repair* or treat* or surg* or (aneurysm? adj1 repair*))).ti,ab.
3. (open adj2 (surg* or repair*)).ti,ab.
4. endoprosthe*.mp.
5. *Aortic Aneurysm, Thoracic/su [Surgery]
6. *Aortic Thoracic/su [Surgery]
7. Thoracic Surgical Procedures/
8. or/1-7
9. exp Aortic Aneurysm, Thoracic/
10. (thorac* adj2 aneurysm*).ti,ab.
11. (descending adj1 aort* adj1 aneurysm*).ti,ab
12. TAA*.ti,ab.
13. (arch adj3 aneurysm*).ti.ab.
14. or/9-13
15. morbid*.mp.
16. mortalit*.mp.
17. outcome?.mp.
18. complication*.mp.
19. (rupture? or endoleak?).mp.
20. device migration.mp.
21. (ae or co or de).fs

For peer review only

22. (safe or safety or side effect* or undesirable effect* or treatment emergent or tolerability or toxicity or adrs or (adverse adj2 (effect or effects or reaction or reactions or event or events to outcome or outcomes))).ti,ab.
23. ((technical or surg*) adj2 (advance* or improve*)).mp.
24. (quality adj2 life).mp.
25. Mortality/
26. Mortbidity/
27. Postoperative complications/
28. Intraoperative complications/
29. Aortic Aneurysm, Thoracic/co
30. Conversion to Open Surgery/
31. “Quality of Life”
32. or/15-31
33. 8 and 14 and 32
34. (“clinical trial” or “clinical trial, phase I” or “clinical trial, phase ii” or clinical trial phase iii or clinical trial, phase iv or controlled clinical trial or “multicentre study” or “randomized controlled trial”).pt. or double-blind method/ or clinical trials, phase iii as topic/ or clinical trials, phase iv as topic/ or controlled clinical trials as topic/ or randomized controlled trials as topic/ or early termination of clincal trials as topic/ or multicenter studies as topic/ or ((randomi?ed adj7 trial*) or (controlled adj3 trial*) or (clinical adj2 trial*) or ((single or doubl* or tripl* or treb*) and (blind* or mask*))).ti,ab,kw. or (“4 arm” or “four arm”).ti,ab,kw.
35. cohort studies/ or longitudinal studies/ or follow-up studies/ or prospective studies/ or retrospective studies/ or cohort.ti,ab. or longitudinal.ti,ab. or prospective.ti,ab. or retrospective.ti,ab. or quasi-experiment.ti,ab.

For peer review only

36. Clinical Trial.pt. or random*.tw. or Clinical Trials/ or clinical trial.tw. or Follow-up Studies/ or prospectiv*.tw. or control*.tw.
37. or/34-36
38. 33 and 37

EMBASE

1. (TEVAR or EVAR).ti,ab.
2. (endovascular adj1 (stent* or repair* or treat* or surg* or (aneurysm? adj1 repair*))).ti,ab.
3. (open adj2 (surg* or repair*)).ti,ab.
4. endoprosthe*.mp.
5. Thoracic Aorta Aneruysm/su [Surgery]
6. Thoracoabdominal aorta aneurysm/su [Surgery]
7. Thoracic Aorta/su [Surgery]
8. exp Thoracic aorta surgery/
9. or/1-8
10. exp Thoracic Aorta Aneurysm/
11. exp Thoracoabdominal aorta aneurysm/
12. Thoracic Aorta/
13. (thorac* adj2 aneurysm*).ti,ab.
14. (descending adj1 aort* adj1 aneurysm*).ti,ab.
15. TAA*.ti,ab.
16. (arch adj3 aneurysm*).ti,ab.
17. or/10-16
18. (outcome? or mortalit*).mp.
19. complication*.mp.
20. ((technical or surg*) adj2 (advance* or improve*)).mp.

For peer review only

21. (quality adj2 life).mp.
22. Mortality/
23. Postoperative complication/
24. peroperative complication/
25. Thoracic Aorta Aneurysm/co
26. “Quality of Life”/
27. or/18-26
28. (Randomised Controlled Trial or Pragmatic Clinical Trial).pt or exp Randomized Controlled Trials as Topic/ or “Randomized Controlled Trial (topic)”/ or Randomized Controlled Trial/ or Randomization/ or Random Allocation/ or Double-Blind Method/ or Double Blind Procedure/ or Double-Blind Studies/ or Single-Blind Method/ or Single Blind Procedure/ or Single-Blind Studies/ or Placebos/ or Placebo/ or (random* or sham or placebo*).ti,ab,hw,kf,kw. or ((singl* or doubl*) adj (blind* or dumm* or mask*)).ti,ab,hw,,kw. or ((tripl* or trebl*) adj (blind* or dumm* or mask*)).ti,ab,hw,,kw.
29. random:.tw. or placebo:.mp. or double-blind:.mp.
30. controlled clinical trial/ or clinical trial/ or exp controlled study/ or randomized controlled trial/ or exp quasi experimental study/
31. or/28-30
32. 9 and 17 and 27 and 31
33. limit 32 to (human and yr=”1994-Current”)
34. exp cohort analysis/ or exp longitudinal study/ or exp prospective study/ or exp follow up/ or cohort*.tw.
35. limit 34 to (human and yr=”1994-Current”)
36. 9 and 17 and 27 and 35
37. 36 not 33
38. 33 or 37

For peer review only

**Appendix 2: Data extraction table**

| Study | **Von Allmen et al 2014** | **Goodney et al 2011** (2 analyses*) | **Gore TAG** | **Hughes et al 2014** | **Piffaretti et al 2007** |
|---|---|---|---|---|---|
| **METHODS** | | | | | |
| **Study Design** | Comparative cohort | Comparative cohort | Comparative cohort | comparative cohort | Comparative cohort |
| **Method of matching** | None | 1. None<br>2. propensity matched | ESG patients judged eligible for OSR | None | OSR patients judged eligible for ESG |
| **Inclusion Criteria** | Hospital diagnosis code 171.2 (TAA without mention of rupture) from ICD-10 score linked to | Patients diagnosed with a thoracic aneurysm according to ICD9 diagnosis code: | Fusiform descending thoracic aortic aneurysm at least twice the size of the normal adjacent aorta or saccular | Nationwide inpatient Sample database of patients >18 years old who had | Elective, Chronic fusiform or saccular aneurysm of the descending thoracic aorta, OSR patients |

For peer review only

| | | | | | |
|---|---|---|---|---|---|
| | operations code according to OPCS-4, linked with all-cause mortality from ONS and ICD-10, only OPCS-4 codes: L18.2, L19.2, L20.2, L21.2 | Intact thoracic aneurysms 441.2<br>Patients had undergone an open or TEVAR procedure according to ICD9 procedure codes:<br>Open 38.35, 38.45<br>TEVAR 39.73, 39.79 | aneurysm, Life expectancy >2 years<br>Surgical candidate<br>Male or infertile female >21 years old<br>Descending aorta must be clampable distal to the left carotid artery, and distal anastomosis must be performed proximal to the celiac axis<br>Inner aortic diameter of 23-37mm adjacent to aneurysm<br>Lack of significant thrombus of calcification in landing zones | undergone an open or endovascular repair of non-ruptured thoracic aortic aneurysm. Patients selected using ICD-9 procedures codes 39.73 and 38.45. | would have been a candidates for ESG due to morphological criteria, Selected on surgeon's opinion of lower risk of ESG |

For peer review only

| | | | | | |
|---|---|---|---|---|---|
| | | | Minimum of 2cm of normal thoracic aorta proximal and distal to aneurysm<br>Aortic taper of no more than 4mm or ability to treat with more than one graft | | |
| **Exclusion Criteria** | Dissection, Abdominal aneurysms, Non-identified area aneurysm, Ascending aneurysm, Suprarenal aneurysm, Non- | Patients with ICD 9 diagnosis codes indicating ascending thoracic aneurysms or cardiopulmonary bypass occurring with circulatory arrest. Thoracoabdominal aneurysm, thoracic aortic | Mycotic aneurysm, Hemodynamically unstable ruptured aneurysm , Major operation (other than planned subclavian to carotid transposition or bypass) within 30 days, MI or CVA within 6 weeks, Creatinine >2.0mg/dL, Connective tissue | NA | Traumatic, Mycotic thoracic aneurysms, Dissections, Aneurysms of the ascending aorta, Aneurysms of the aortic arch, Aortic ulcers |

For peer review only

| | | | | | |
|---|---|---|---|---|---|
| | aortic procedures, age <50yrs | dissection, other aortic pathology. IC9 procedure codes indicating debranching or other procedures to extend endovascular landing zones (39.24, 39.25, | disorder, Acute or chronic aortic dissection, Planned occlusion of carotid or celiac arteries, Documented drug abuse within 6 months, Participation in another investigational device or drug study within 1 year | | |
| **Definition of the short-term** | 30 days post-procedure | latest occurring of discharge or 30 days post-intervention | latest occurring of discharge or 30 days post-intervention | post-procedure hospital stay | 30-days post-intervention |
| **PARTICIPANTS** | | | | | |
| **Country** | UK | USA | USA | USA | Italy |

For peer review only

| | | | | | |
|---|---|---|---|---|---|
| **Setting** | Multi-centre – Hospital episode statistics | Multi-centre – Medicare data | Multi-centre | Multi-centre | Single-centre |
| **Time frame** | 2006 to 2011 | 1. 1998-2007<br>2. 2003-2007 | ESG 1999- 2001, OSR pre 1999 (n=44) & 1999-2001 (n=50) | 1998-2007 | ESG 2000-2007,<br>OSR 1996-2000 |
| **Recruitment** | Total 618<br>ESG 354<br>OSR 264 | 1. Total 13,998 (ESG 2433, OSR 11,565)<br>2. Total 1100 (ESG 550, OSR 550) | Total 234<br>ESG 140<br>OSR 94 | Total 8967<br>ESG 712<br>OSR 8255 | Total 28<br>ESG 17<br>OSR 11 |
| **Age summary** | median (IQR)<br>ESG 73(66,78), OSR 71 (63-76) | mean (95% C.I.)<br>1. ESG 75.9 (75.6, 76.1), OSR 73.8 (73.7, 73.9)<br>2.<br>ESG 71.1 (70.8, 71.44),<br>OSR 70.7 (70.7, 71.1) | mean(SD)<br>ESG 75.9 (10.4), OSR 68.2 (10.2) | median (IQR)<br>ESG 72 (63- 78),<br>OSR 63 (52, 72) | mean (SD)<br>ESG 66 (10),<br>OSR 61 (13) |

For peer review only

| | | | | | |
|---|---|---|---|---|---|
| **Proportion male** | ESG 232 (65.5%), OSR 137 (51.9%) | % (95% C.I.): 1. ESG 58.7 (56.7, 60.7), OSR 55.4 (54.8, 56.3) 2. ESG 64.0 (59.9, 68.0), OSR 68.1% (64.2, 72.0) | ESG 90(57%), OSR 48 (51%) | 5415 (61.3%) ESG, 437 (65.6%) OSR | ESG 14 (82%), OSR 8 (73%) |
| **Black race** | NR | % (95% C.I.): 1. ESG 7.5 (6.4, 8.5), OSR 3.8 (3.4, 4.5) (p=0.001) 2. ESG 2.7 (1.4, 4.1), OSR 2.7 (1.4, 4.0) (p=0.99) | NR | NR | NR |
| **Charlson comorbidity score** | NR | % (95% C.I.): 1. ESG 1.46 (1.38, 1.54), OSR 0.83 (0.80, 0.86) (p=0.00001) | NR | NR | NR |

For peer review only

| | | | | | |
|---|---|---|---|---|---|
| | | 2. ESG 0.5 (0.41, 0.60), OSR 0.44 (0.35, 0.53) (p=0.34) | | | |
| **Diabetes** | NR | % (95% C.I.): 1. ESG 8.00 (6.7, 8.8), OSR 4.10 (3.7, 4.5) (p=0.001) 2. ESG 4.90 (3.0, 6.7), OSR 2.90 (1.5, 4.3) (p=0.09) | NR | 684 (13.2%) ESG, 94 (8.3%) OSR (p<0.001) | NR |
| **Myocardial infarction** | NR | % (95% C.I.): 1. ESG 7.00 (6.00, 8.1), OSR 5.10 (4.6, 5.4) (p=0.001) 2. ESG 2.50 (1.2, 3.8), OSR 3.40 (1.9, 4.9) (p=0.38) | -NR | NR | NR |
| **Congestive heart failure** | NR | % (95% C.I.): | ESG 13 (9%), OSR 9 (10%) (p=1.00) | NR | NR |

For peer review only

| | | | | | |
|---|---|---|---|---|---|
| | | 1. ESG 10.85% (9.6%-12.0%), OSR 10.60% (10.1%- 11.2%) (p=0.16)<br>2. ESG 3.00 (1.6, 4.5), OSR 4.70 (2.9, 6.5) (p=0.16) | | | |
| **Cerebrovascular disease** | NR | % (95% C.I.):<br>1. ESG 5.09% (4.2%-5.9%), OSR 3.20% (2.9%- 3.5%) (p=0.65)<br>2. ESG 2.00 (0.5, 2.6), OSR 2.00 (0.8, 3.1) (p=0.65) | NR | NR | 4 (23%) ESG, 1 (9%) OSR (p=NS) |
| **COPD** | NR | % (95% C.I.):<br>1. ESG 22.04% (20.3%-23.6%), OSR 10.65% (10.0%-11.2%) (p=0.47) | ESG 56 (40%), OSR 36 (38%) (p=0.89) | 247 (34.7%) ESG, 1483 (17.9%) (P<0.001) | 10 (59%) ESG, 6 (54%) OSR (p=NS) |

For peer review only

| | | | | | |
|---|---|---|---|---|---|
| | | 2. ESG 4.00 (2.3, 5.6), OSR 4.90 (3.0, 6.7) (p=0.47) | | | |
| **Chronic renal failure** | NR | % (95% C.I.):<br>1. ESG 4.9% (4.0%- 5.7%), OSR 0.88% (0.07%- 0.10%) (p=0.70)<br>2. ESG 0.01 (0, 1.4), OSR 0.50 (0, 1.1) (p=0.70) | NR | 90 (12.6%) ESG, 126 (1.5%) OSR (p<0.001) | 4 (23%) ESG, 1 (9%) OSR (p=NS) |
| **History of malignancy** | NR | % (95% C.I.):<br>1. ESG 5.71% (4.7%-6.6%), OSR 2.91% (2.6%- 3.2%) (p=0.82)<br>2. ESG 1.80 (0.6, 2.9), OSR 1.60 (0.5, 2.6) (p=0.82) | NR | NR | NR |
| **History of smoking (current or past)** | NR | NR | ESG 117 (84%), OSR 77 (82%) (p=0.86) | NR | NR |

For peer review only

| | | | | | |
|---|---|---|---|---|---|
| **BMI (kg/m2)** | NR | NR | mean (SD): ESG 26.4 (4.7), OSR 26.9 (5) (p=0.44) | NR | NR |
| **Coronary artery disease** | NR | NR | ESG 69 (49%), OSR 34 (36%) (p=0.60) | NR | NR |
| **Stroke** | NR | NR | ESG 14 (10%), OSR 9 (9%) (p=1.00) | NR | NR |
| **Peripheral arterial disease** | NR | NR | ESG 22 (16%), OSR 10 (11%) (p=0.33) | NR | NR |
| **Symptomatic aneurysm** | NR | NR | ESG 30 (21%), OSR 36 (38%) (p=0.007) | NR | NR |
| **Other concomitant aneurysm:** | NR | NR | ESG 39 (28%), OSR 26 (28%) (p=1.00) | NR | NR |
| **Renal dialysis** | NR | NR | ESG 2 (1%), OSR 0 (0%) (p=0.52) | NR | NR |

For peer review only

| | | | | | |
|---|---|---|---|---|---|
| **Paraplegia** | NR | NR | ESG 1 (1%), OSR 0 (0%) (p=1.00) | NR | NR |
| **Cancer** | NR | NR | ESG 27 (19%), OSR 12 (13%) (p=0.21) | NR | NR |
| **Prior major vascular surgery** | NR | NR | ESG 63 (45%), OSR 52 (55%) (p=0.14) | NR | NR |
| **Cardiac comorbidities** | NR | NR | NR | 265 (37.2%) ESG, 3535 (42.8%) (p<0.001) | NR |
| **Hypertension** | NR | NR | NR | NR | 16 (94%) ESG, 10 (91%) OSR (p=NS) |
| **Peripheral obstructive disease** | NR | NR | NR | NR | 5 (29%) ESG, 2 (18%) OSR (p=NS) |
| **Ischemic heart disease** | NR | NR | NR | NR | 6 (35%) ESG, 5 (45%) OSR (p=NS) |

For peer review only

| | | | | | |
|---|---|---|---|---|---|
| **Cardiac Surgery** | NR | NR | NR | NR | (18%) ESG, 0 (0%) OSR (p=NS) |
| **SHORT- TERM OUTCOMES** | | | | | |
| **All-cause mortality** | ESG 21 (5.9%) OSR 4 (1.5%) Adjusted OR: 0.79 | 1. ESG 6.1% (95%CI 5.1%, 7.0%) OSR 7.1% (95%CI 6.7%, 7.6%) 2. ESG 4.2% (95% C.I. 2.5%-5.8%) OSR 4.5% (95% C.I. 2.8%-6.2%) | ESG 3 (2.1%) OSR 11 (11.7%) Adjusted OR: 0.54 | ESG 25 OSR 379 | ESG 1 (6%) OSR 2 (20%) |
| **Re-intervention** | ESG 21 (5.9%) OSR 4 (1.5%) Adjusted OR: 2.97 (1.09, 8.10) | NR | NR | NR | NR |

For peer review only

| | | | | | |
|---|---|---|---|---|---|
| **Paraplegia/ Paraperisis** | NR | NR | ESG 4 (3%)<br>OSR 13 (14%) | NR | NR |
| **Cerebral Vascular Accident** | NR | NR | ESG 5 (4%)<br>OSR 5 (4%) | NR | ESG 2 (9%)<br>OSR 1 (12%) |
| **Neurological-unspecified type** | NR | NR | NR | ESG 20 (2.8%)<br>OSR 273 (3.3%)<br>Adjusted OR: 0.38 | NR |
| **Respiratory failure** | NR | NR | ESG 5 (4%)<br>OSR 19 (20%) | NR | NR |
| **Pneumonia** | NR | NR | NR | NR | ESG 2 (12%)<br>OSR 3 (27%) |
| **Pulmonary-unspecified type** | NR | NR | NR | ESG 17 (2.4%)<br>OSR 462 (5.6%)<br>Adjusted OR: 0.48 | NR |
| **Myocardial Infarction** | NR | NR | ESG 0 (0%)<br>OSR 1(1%) | NR | ESG 0 (0%)<br>OSR 1 (9%) |

For peer review only

| | | | | | |
|---|---|---|---|---|---|
| **Cardiac-unspecified type** | NR | NR | NR | ESG 28 (2.9%)<br>OSR 1252 (15.2%)<br>Adjusted OR: 0.24 | NR |
| **Peripheral vascular disease** | NR | NR | ESG 20 (14%)<br>OSR 4 (4%) | NR | NR |
| **Renal failure** | NR | NR | ESG 2 (1%)<br>OSR 12 (13%) | NR | NR |
| **Wound infection/dehiscence** | NR | NR | ESG 5 (4%)<br>OSR 10 (11%) | NR | NR |
| **GI complication** | NR | NR | ESG 3 (2%)<br>OSR 6 (6%) | NR | NR |
| **Post-implant syndrome** | NR | NR | NR | NR | ESG 4(18%)<br>OSR 0(0%) |
| **LONG-TERM OUTCOMES** | | | | | |

For peer review only

| | | | | | |
|---|---|---|---|---|---|
| All-cause survival | 1 year adjusted OR: 1.1 95%CI [0.70-1.73], p=0.667<br>5 year adjusted HR: 1.45 95%CI [1.08-1.94], p=0.013 | NR | NR | NR | NR |
| **aortic-related survival** | 5 year adjusted HR: 1.27 95%CI [0.81-1.98], p=0.298 | NR | NR | NR | NR |
| **survival from aortic-related re-intervention** | 5 year adjusted HR: 1.70 95%CI [1.11-2.60], p=0.014 | NR | NR | NR | NR |
| * 1. Unadjusted cohort, 2. Propensity matched cohort | | | | | |

For peer review only

**Appendix 3: Risk of Bias assessment**

*Risk of Bias Results using the ROBINS-I risk of bias tool*

| Domain – source of bias | Goodney et al 2011 (2 analyses*) | Gore TAG | Hughes et al 2014 | Piffaretti et al 2007 | Von Allmen et al 2014 |
|---|---|---|---|---|---|
| **Confounding** | 1. Severe<br>2. Moderate | Severe | Severe | Severe | Severe |
| **Selection of participants into the study** | 1. Low<br>2. Low | Low | Low | Low | Low |
| **Classification of interventions** | 1. Low<br>2. Low | Low | Low | Low | Low |
| **Deviations from intended interventions** | 1. Low<br>2. Low | Low | Low | Low | Low |
| **Missing data** | 1. Low<br>2. Low | Low | Low | Low | Low |
| **Measurement of outcomes** | 1. Low<br>2. Low | Low | Low | Low | Low |
| **Bias in selection of the reported result** | 1. Moderate<br>2. Moderate | Moderate | Moderate | Moderate | Moderate |
| **Overall** | 1. Severe<br>2. Moderate | Severe | Severe | Severe | Severe |

* 1. Unadjusted cohort, 2. Propensity matched cohort

For peer review only

| Section/topic | # | Checklist item | Reported on page # |
|---|---|---|---|
| **TITLE** | | | |
| Title | 1 | Identify the report as a systematic review, meta-analysis, or both. | 1 |
| **ABSTRACT** | | | |
| Structured summary | 2 | Provide a structured summary including, as applicable: background; objectives; data sources; study eligibility criteria, participants, and interventions; study appraisal and synthesis methods; results; limitations; conclusions and implications of key findings; systematic review registration number. | 2 |
| **INTRODUCTION** | | | |
| Rationale | 3 | Describe the rationale for the review in the context of what is already known. | 4 |
| Objectives | 4 | Provide an explicit statement of questions being addressed with reference to participants, interventions, comparisons, outcomes, and study design (PICOS). | 5 |
| **METHODS** | | | |
| Protocol and registration | 5 | Indicate if a review protocol exists, if and where it can be accessed (e.g., Web address), and, if available, provide registration information including registration number. | 6 |
| Eligibility criteria | 6 | Specify study characteristics (e.g., PICOS, length of follow-up) and report characteristics (e.g., years considered, language, publication status) used as criteria for eligibility, giving rationale. | 6 |
| Information sources | 7 | Describe all information sources (e.g., databases with dates of coverage, contact with study authors to identify additional studies) in the search and date last searched. | 7 |
| Search | 8 | Present full electronic search strategy for at least one database, including any limits used, such that it could be repeated. | 7 |
| Study selection | 9 | State the process for selecting studies (i.e., screening, eligibility, included in systematic review, and, if applicable, included in the meta-analysis). | 8 |
| Data collection process | 10 | Describe method of data extraction from reports (e.g., piloted forms, independently, in duplicate) and any processes for obtaining and confirming data from investigators. | 8 |

For peer review only

| | | | |
|---|---|---|---|
| Data items | 11 | List and define all variables for which data were sought (e.g., PICOS, funding sources) and any assumptions and simplifications made. | 8 |
| Risk of bias in individual studies | 12 | Describe methods used for assessing risk of bias of individual studies (including specification of whether this was done at the study or outcome level), and how this information is to be used in any data synthesis. | 8 |
| Summary measures | 13 | State the principal summary measures (e.g., risk ratio, difference in means). | 9 |
| Synthesis of results | 14 | Describe the methods of handling data and combining results of studies, if done, including measures of consistency (e.g., $I^2$) for each meta-analysis. | 9 |



| **Section/topic** | **#** | **Checklist item** | **Reported on page #** |
|---|---|---|---|
| Risk of bias across studies | 15 | Specify any assessment of risk of bias that may affect the cumulative evidence (e.g., publication bias, selective reporting within studies). | 9 |
| Additional analyses | 16 | Describe methods of additional analyses (e.g., sensitivity or subgroup analyses, meta-regression), if done, indicating which were pre-specified. | 9 |
| **RESULTS** | | | |
| Study selection | 17 | Give numbers of studies screened, assessed for eligibility, and included in the review, with reasons for exclusions at each stage, ideally with a flow diagram. | 9 |
| Study characteristics | 18 | For each study, present characteristics for which data were extracted (e.g., study size, PICOS, follow-up period) and provide the citations. | Table 1, appendix 2 |
| Risk of bias within studies | 19 | Present data on risk of bias of each study and, if available, any outcome level assessment (see item 12). | 14, appendix 3 |
| Results of individual studies | 20 | For all outcomes considered (benefits or harms), present, for each study: (a) simple summary data for each intervention group (b) effect estimates and confidence intervals, ideally with a forest plot. | 14-19 |
| Synthesis of results | 21 | Present results of each meta-analysis done, including confidence intervals and measures of consistency. | 14-19 |
| Risk of bias across studies | 22 | Present results of any assessment of risk of bias across studies (see Item 15). | 14-19 |
| Additional analysis | 23 | Give results of additional analyses, if done (e.g., sensitivity or subgroup analyses, meta-regression [see Item 16]). | NA |

For peer review only

| DISCUSSION | | | |
|---|---|---|---|
| Summary of evidence | 24 | Summarize the main findings including the strength of evidence for each main outcome; consider their relevance to key groups (e.g., healthcare providers, users, and policy makers). | 19-21 |
| Limitations | 25 | Discuss limitations at study and outcome level (e.g., risk of bias), and at review-level (e.g., incomplete retrieval of identified research, reporting bias). | 21 |
| Conclusions | 26 | Provide a general interpretation of the results in the context of other evidence, and implications for future research. | 22 |
| **FUNDING** | | | |
| Funding | 27 | Describe sources of funding for the systematic review and other support (e.g., supply of data); role of funders for the systematic review. | 22 |

*From:* Moher D, Liberati A, Tetzlaff J, Altman DG, The PRISMA Group (2009). Preferred Reporting Items for Systematic Reviews and Meta-Analyses: The PRISMA Statement. PLoS Med 6(7): e1000097. doi:10.1371/journal.pmed1000097

For more information, visit: **www.prisma-statement.org**.



For peer review only

For peer review only - http://bmjopen.bmj.com/site/about/guidelines.xhtml
